# Rapid identification of chemical profiles *in vitro* and *in vivo* of Huan Shao Dan and potential anti-aging metabolites by high-resolution mass spectrometry, sequential metabolism, and deep learning model

**DOI:** 10.3389/fphar.2024.1432592

**Published:** 2024-10-15

**Authors:** Xueyan Li, Fulu Pan, Lin Wang, Jing Zhang, Xinyu Wang, Dongying Qi, Xiaoyu Chai, Qianqian Wang, Zirong Yi, Yuming Ma, Yanli Pan, Yang Liu, Guopeng Wang

**Affiliations:** ^1^ School of Chinese Materia Medica, Beijing University of Chinese Medicine, Beijing, China; ^2^ Institute of Information on Traditional Chinese Medicine, China Academy of Chinese Medical Sciences, Beijing, China; ^3^ Zhongcai Health (Beijing) Biological Technology Development Co., Ltd., Beijing, China

**Keywords:** Huan Shao Dan, sequential metabolism, UPLC-Q Exactive-Orbitrap HRMS, deep learning model, anti-aging metabolites

## Abstract

**Background:**

Aging is marked by the gradual deterioration of cells, tissues, and organs and is a major risk factor for many chronic diseases. Considering the complex mechanisms of aging, traditional Chinese medicine (TCM) could offer distinct advantages. However, due to the complexity and variability of metabolites in TCM, the comprehensive screening of metabolites associated with pharmacology remains a significant issue.

**Methods:**

A reliable and integrated identification method based on UPLC-Q Exactive-Orbitrap HRMS was established to identify the chemical profiles of Huan Shao Dan (HSD). Then, based on the theory of sequential metabolism, the metabolic sites of HSD *in vivo* were further investigated. Finally, a deep learning model and a bioactivity assessment assay were applied to screen potential anti-aging metabolites.

**Results:**

This study identified 366 metabolites in HSD. Based on the results of sequential metabolism, 135 metabolites were then absorbed into plasma. A total of 178 peaks were identified from the sample after incubation with artificial gastric juice. In addition, 102 and 91 peaks were identified from the fecal and urine samples, respectively. Finally, based on the results of the deep learning model and bioactivity assay, ginsenoside Rg1, Rg2, and Rc, pseudoginsenoside F11, and jionoside B1 were selected as potential anti-aging metabolites.

**Conclusion:**

This study provides a valuable reference for future research on the material basis of HSD by describing the chemical profiles both *in vivo* and *in vitro*. Moreover, the proposed screening approach may serve as a rapid tool for identifying potential anti-aging metabolites in TCM.

## 1 Introduction

Abundant natural resources can significantly expedite drug research and development. Research indicates that over 50% of drugs marketed in the past three decades originated from natural sources (Bent, 2008; Li and Vederas, 2009; Newman and Cragg, 2012). Traditional Chinese medicine (TCM), a crucial source of natural resources, offers distinct advantages in treating chronic diseases (Ma et al., 2017; Xue et al., 2013). Although there is consensus on the pharmacodynamic effects of TCM, the comprehensive screening of metabolites associated with pharmacological activities and further elucidation of their material base remain significant issues (Liao et al., 2018). Modern pharmacology believes that in order to achieve desired therapeutic effect, the majority of drugs need to be transported to the tissues or targets through blood circulation and sustain a therapeutic blood concentration (Bach et al., 2019). As such, compared with traditional extraction–separation screening approaches, research on comprehensive and efficient absorbed metabolites is urgently needed. After oral administration, drugs are mainly metabolized sequentially by gastric juice, the intestinal cavity, and the liver, are absorbed from the small intestine, and are excreted through urine and feces. Hence, for most oral agents, particularly multi-component drugs, attention should be directed to understanding the spatial sequence of drug entry into the organism’s digestive tract, termed the “sequential metabolism theory” (Luo et al., 2021; Luo et al. 2013; Luo et al. 2016; Zhang et al., 2016).

The metabolites in TCM formulas are diverse and complex, making it challenging to experimentally confirm the target of each metabolite. Employing statistical and machine learning models for drug-target affinity (DTA) prediction could expedite the matching of each metabolite to its interacting target (Ozturk et al., 2018). Machine learning models have been utilized in numerous studies to integrate training data derived from biological screens and publicly available databases (Lavecchia, 2019). Chemical activities and pharmacological properties can be predicted using these models, which often incorporate neural network architectures (Askr et al., 2023). Additionally, they aid in the discovery of molecular binding targets and aging biomarkers, as well as in the design of molecules that meet specific criteria pertaining to biological activity and physicochemical properties. For instance, Wong et al. (2023) employed deep learning methods to rapidly discover senolytic small molecules from a very large database. Despite the successful use of machine learning methodologies, further development, testing, and application of these approaches in the area of senolytics are still necessary. It is crucial to establish appropriate conceptual frameworks, generate well-controlled training data, select appropriate model architectures, and experimentally validate the model predictions in these applications. These factors play a significant role in assessing the predictive accuracy of the models and demonstrating the efficacy of machine learning in the discovery of chemical compounds (Aittokallio, 2022).

Huan Shao Dan (HSD), a classical TCM prescription, has been recorded in Jiyang of *materia medica* published in the Ming dynasty of China, including *Polygonum multiflorum* Thunb., *Achyranthes bidentata* Bl., *Rehmannia glutinosa* Libosch., *Cistanche deserticola* Y.C.Ma, *Phellodendron chinense* Schneid., *Psoralea corylifolia* L., *Plantago asiatica* L., *Platycladus orientalis* (L.) Franco, *Dioscorea opposita* Thunb., *Angelica sinensis* (Oliv.) Diels, *Cuscuta australis* R.Br., *Panax ginseng* C. A. Mey., and *Schisandra chinensis* (Turcz.) Baill (Wu, 1995). Previous pharmacological experiments have indicated that HSD and its modified formula possess antioxidant properties, alleviate depressive symptoms, and potentially exhibit anti-aging effects (Cheng et al., 2012; Hu et al., 2012). However, there have been few systematic studies on the chemical and metabolic profile of HSD, greatly hindering its in-depth investigation.

**FIGURE 1:**
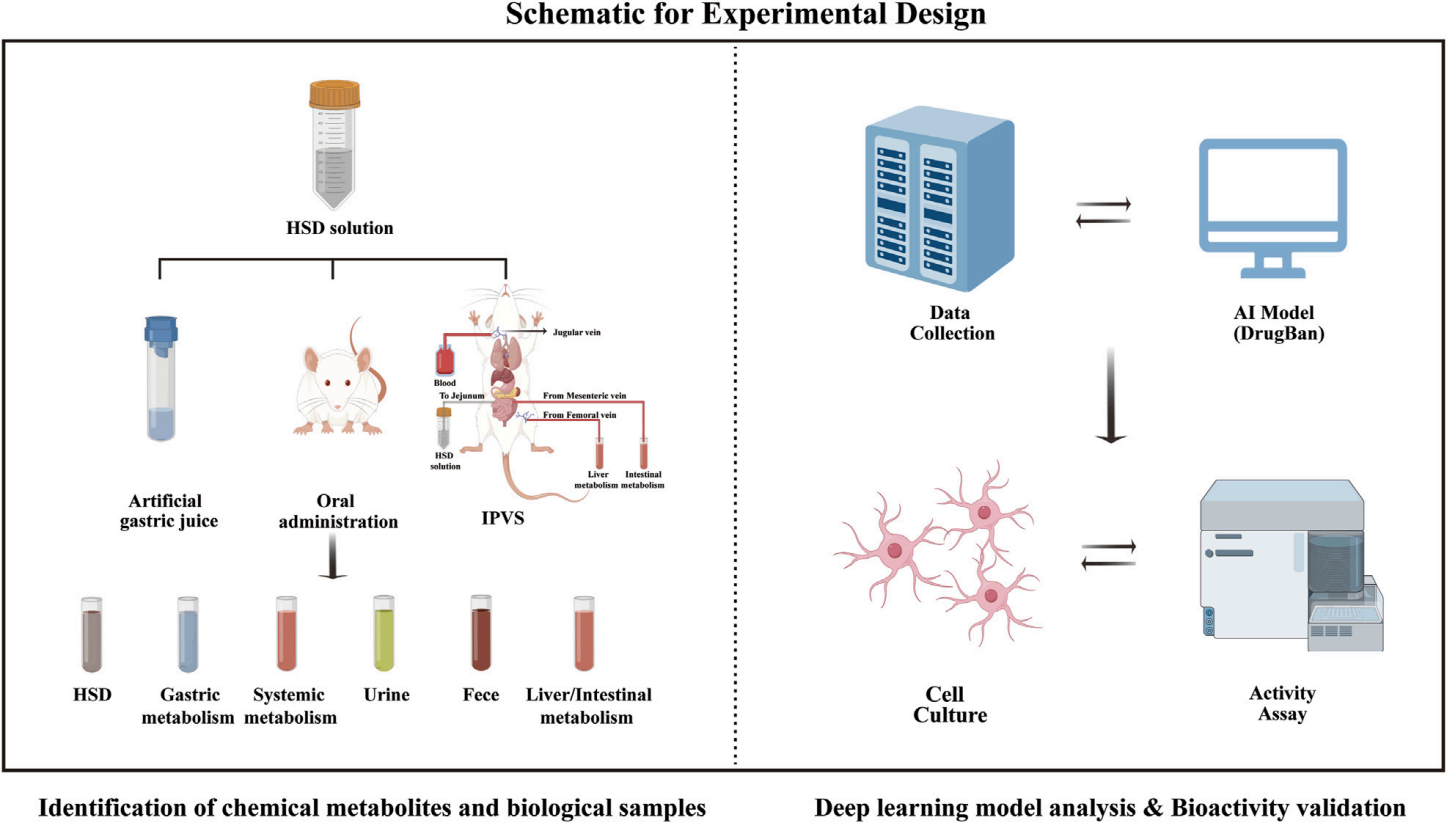
Schematic representation of the experimental design.

Therefore, this study employed a comprehensive identification approach based on UPLC-Q Exactive-Orbitrap HRMS and a deep learning model to identify chemical profiles *in vivo* and *in vitro*, as along with potential anti-aging metabolites. The schematic for the experimental design is shown in Figure 1. First, the chemical metabolites of the 13 botanical drugs comprising HSD were identified using UPLC-Q Exactive-Orbitrap HRMS. Then, based on sequential metabolism theory, metabolites from various sample types were identified. Finally, a deep learning model was employed to screen potential anti-aging metabolites, which were then verified through bioactivity assessment assays. It is noteworthy that this is the first report describing the chemical profiles of HSD both *in vivo* and *in vitro*, providing a valuable reference about its material basis for future research.

## 2 Methods

### 2.1 Reagents

Every TCM material used was purchased from Tongrentang Co., Ltd. (Beijing, China) and authenticated by Prof. Jingjuan Wang. Detailed information on reagents and reference standards are described in the Supplementary Material.

### 2.2 UPLC-MS for metabolite analysis and biological samples

Sample analysis was performed on a Vanquish Horizon UPLC system with a Q Exactive Hybrid Quadrupole-Orbitrap high-resolution mass spectrometer (Thermo Fisher Scientific, United States). Chromatographic separation was conducted on Waters ACQUITY UPLC BEH Shield RP C18 column (100 mm × 2.1 mm, 1.7 µm) maintained at 35°C with a flow rate of 0.3 mL/min. Mobile phase A was 0.1% aqueous formic acid (v/v), and mobile phase B was acetonitrile. The gradient was set as follows: 0–1 min, 95% A; 1–3 min, 95%–90% A; 3–15 min, 90%–70% A; 15–32 min, 70%–10% A; 32–34 min, 10–95% A; 34–36 min, 95% A.

Mass spectrometric detection with H-ESI was performed with the following settings: spray voltage of 3.8 kV (positive) and 3.2 kV (negative); sheath gas and auxiliary gas flow rate, 35 and 15; auxiliary gas heater temperature, 300°C; capillary temperature, 350°C; mass range, *m/z* 100–1,500. The dd-MS2 were obtained at resolutions of 17,500 and 70,000 for full mass. The collision energy values were 20%, 40%, and 60%.

### 2.3 Identification of metabolites in HSD

#### 2.3.1 Sample preparation for chemical analysis

The reference standards were mixed and dissolved in methanol. An accurately weighed 100 g sample of HSD powder was ultrasonically extracted with 1,000 mL (1:10, w/v) ethanol (50% v/v) for 1 h. The extracted solution was then filtered, and the precipitate was extracted with another 1,000 mL of ethanol (50% v/v) for 1 h. After filtration, the supernatants were combined and concentrated to 0.25 g crude drug per milliliter. After dilution to 0.05 g/mL, the sample was filtered through a 0.22 μm membrane for LC-MS analysis.

### 2.4 Animal experiments based on sequential metabolism theory

Adult male SD rats (250–300 g) were obtained from SPF Biotechnology Co., Ltd. (Beijing, China). The animals were adaptively raised for 7 days and fasted overnight before the experiment.

#### 2.4.1 Stability in artificial gastric juice

The experiment was conducted using artificial gastric juice according to the Chinese Pharmacopoeia. The specific steps were as follows. In total, 16.4 mL of diluted hydrochloric acid was added to 800 mL of water, followed by 10 g of pepsin. After thorough mixing, water was added to a final volume of 1,000 mL. HSD extracts were added to artificial gastric juice at a ratio of 1:50 and incubated in a 37°C shaking water bath for 2 h, followed by 0.1 M NaOH. LC-MS analysis was carried out after centrifugation at 10,000 rpm for 10 min, followed by filtration through a 0.22 μm membrane.

#### 2.4.2 *In situ* intestinal perfusion with venous sampling (IPVS) surgical procedures

IPVS procedures were performed following Yang et al. (2021) with minor adjustments. Prior to the IPVS, several rats were randomly selected after 12 h of fasting. Blood was collected from the abdominal aorta and kept in a 37°C water bath until administration to the recipient rat. After being anesthetized, the rats were positioned supine on the operating table and their body temperature was maintained using an infrared lamp. The jugular vein was carefully dissected, an intravenous needle was inserted, and one end was connected to a blood reservoir via a peristaltic pump for transfusion. The abdominal cavity was carefully opened along the midline of the rat’s abdomen. A segment of the jejunum (approximately 10 cm) was selected as the test intestine, and the blood vessels beyond this segment were ligated. The intestinal contents were slowly flushed out with 37°C saline until the effluent became clear. A syringe pump was connected to one end of the jejunal segment, and the HSD decoction was instilled at a flow rate of 0.2 mL/min for 2 h. Subsequently, an intravenous needle was inserted into the mesenteric vein (intestinal metabolism) or femoral vein (hepatic metabolism) with ligation of the hepatic portal vein. The blood was pumped at a flow rate of 0.3 mL/min and collected for 2 h.

#### 2.4.3 Oral drug administration

SD rats were randomly divided into five groups of blank and HSD-treated rats at different administration times, each group containing six rats. They were fasted for 12 h before the experiment. The administration dose for SD rats was primarily based on the clinical usage dose, which was calculated as the equivalent dose between humans and rats according to body surface area. The HSD-treated groups were given HSD solution (0.54 g/kg/d, half of the clinical dose) intragastrically at 10 mL/kg, and the blank group was given saline instead, once a day, for 1 week.

In this study, we selected four time points (0.5, 1,1.5, and 2 h) for blood sampling, primarily based on the pharmacokinetic properties of TCM. According to the literature review and preliminary experimental explorations by our research team, we found that collecting blood within 2 h could encompass a wider range of metabolites. As such, after 0.5, 1, 1.5, and 2 h, blood samples were collected from the abdominal aorta of rats after they were anesthetized. Meanwhile, another six rats were also divided into two groups and treated with saline or HSD solution. Urine and feces were collected from 0 to 72 h.

#### 2.4.4 Biological sample pretreatment

Plasma samples: centrifugation at 4,000 rpm at 4°C for 15 min to obtain 20 mL plasma was followed by precipitation with three times the volume of acetonitrile, then centrifugation at 12,000 rpm for 20 min. The supernatant was dried in nitrogen at 50°C and redissolved in 1 mL of acetonitrile.

Urine samples: urine was concentrated to 25 mL and mixed with an equal volume of acetonitrile, followed by centrifugation at 12,000 rpm for 20 min at 4°C. The supernatant was dried under nitrogen at 50°C, and the residue was redissolved in 5 mL of acetonitrile.

Fecal samples: feces were dried and then crushed into a crude powder. The powder (5.0 g) was ultrasonically extracted twice, each time for 30 min with 50 mL of acetonitrile. After centrifugation at 12,000 rpm for 20 min, the supernatant was concentrated and redissolved in 1 mL of acetonitrile.

### 2.5 Deep learning model analysis

#### 2.5.1 Data collection and preparation

A PubChem dataset containing compounds with experimentally confirmed senolytic activity was used for this study as the training dataset. A curation process was performed to eliminate duplicates, inorganic materials, and mixtures after presenting them by SMILES. Briefly, we conducted a search using the keywords “anti-aging” and “senolytic” and subsequently downloaded the relevant activity data, including compound information and activity classifications of aging targets. Target proteins “UniProt ID,” small molecule “Substance ID,” and activity categories were extracted. In PubChem, the activity categories were divided into four categories: “Inactive,” “Active,” “Inclusive,” and “Unspecified.” We classified the data with the activity category “Active” as positive samples (active inhibitors) and labeled them “1”, while the remaining activity categories were treated as negative samples (non-active inhibitors) and labeled “0”. We obtained the SMILES of active small molecules from the PubChem website using “Substance ID” and the protein sequences from the UniProt database using UniProt ID (https://www.uniprot.org/). Therefore, a dataset of 5,945 protein-drug samples (5,174 negative; 771 positive) was acquired. The data were classified into a training set (4,133 negative; 623 positive), a validation set (521 negative; 73 positive), and a test set (520 negative; 75 positive).

#### 2.5.2 Establishing a deep learning model

**FIGURE 2:**
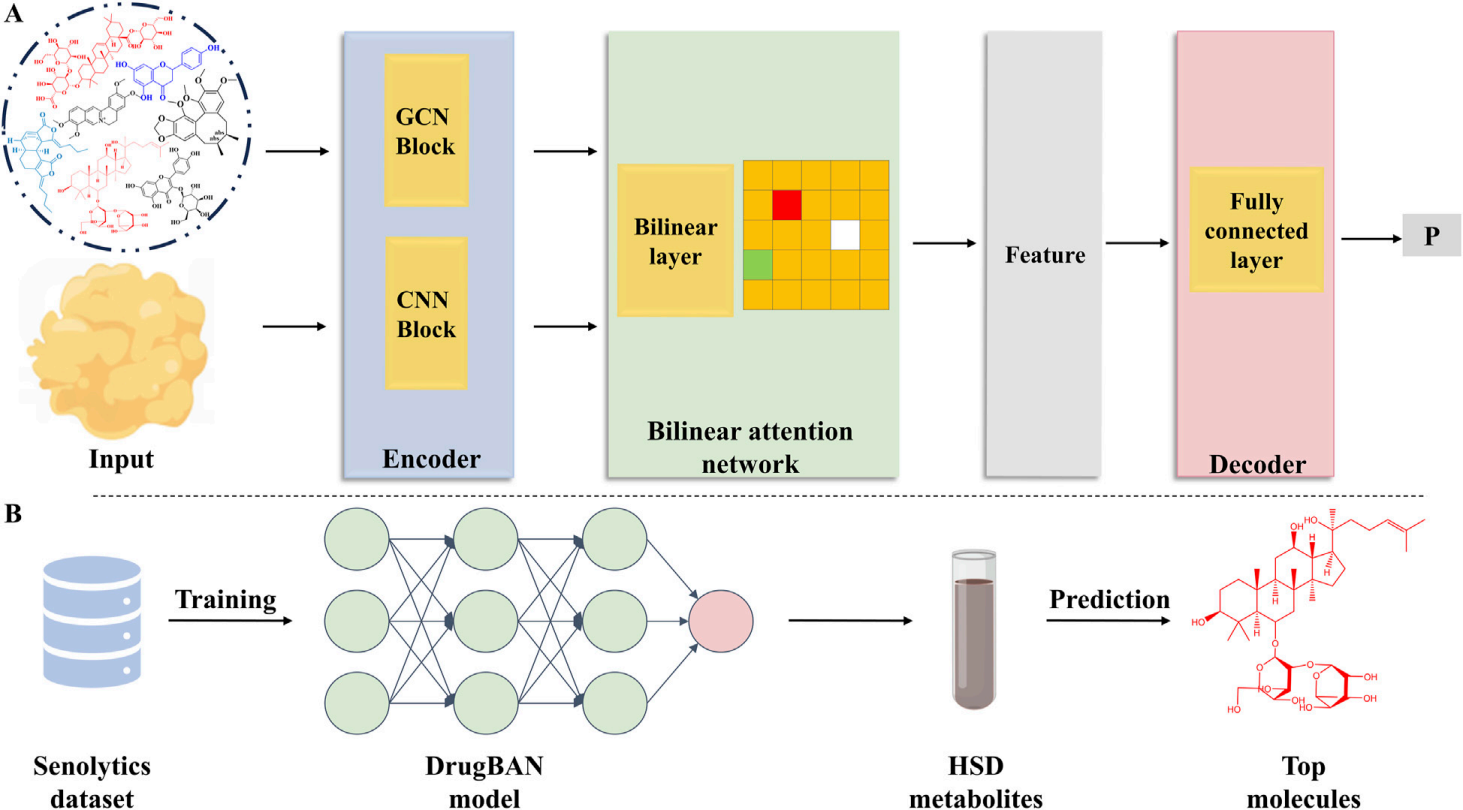
Deep learning model and the prediction process. **(A)** DrugBAN model. **(B)** Deep learning model training and prediction process.

Based on DrugBAN—a bilinear attention network (BAN) framework with adversarial domain adaptation designed for learning pairwise drug-target interactions—this study used deep learning to learn pairwise interactions (Liu et al., 2023) (Figure 2A).

##### 2.5.2.1 Model input

Protein sequences and SMILES representations of compounds were used in this study.

##### 2.5.2.2 Drug encoder

In the context of the drug encoder, each SMILES string was transformed into a 2D molecular graph, denoted as 𝒢𝒢. To represent node-specific information within 𝒢𝒢, an atom node was initiated based on its chemical attributes using the DGL-LifeSci package. Atoms were represented by 74-dimensional integer vectors, comprising eight key characteristics: aromaticity indication, total hydrogen atom count, formal charge, atom type, implicit hydrogen atoms count, atom degree, number of radical electrons, and atom hybridization. To maintain consistency with the maximum allowable length of the protein sequence, we imposed a predefined limit on the number of nodes, denoted as Θ_d_. For molecules with fewer nodes, virtual nodes were introduced and zero-padded as needed. Consequently, the node feature matrix for each graph was denoted as M_d_ ∈ ℝ^Θd×74^. Furthermore, we employed a linear transformation to establish X_d_, represented as Xd = W_0_M^⊤^_d_, resulting in a real-valued dense matrix X_d_ ∈ ℝ^Θd×Dd^, which serves as the input feature.

To effectively learn graph representations, we utilized a three-layer GCN block. Graph convolutional networks (GCNs) generalize convolutional operators to irregular domains. Our approach involved updating the atom feature vectors by aggregating information from their corresponding sets of neighboring atoms. Substructural details of the molecule are inherently captured by this propagation mechanism. For the subsequent exploration of local interactions with protein fragments, we retained the drug representation at the node level.

##### 2.5.2.3 Protein encoder

There are three successive 1D convolutional layers in a protein encoder that convert protein sequences into latent features. In this matrix, each row describes a subsequence of the protein. Drawing inspiration from word embedding principles, we initially initialized all amino acids using a trainable embedding matrix, denoted as Ep ∈ ℝ^23×Dp^. By performing a lookup operation on Ep, each protein sequence 𝒫𝒫 could be assigned a matching feature matrix—Xp ∈ ℝ^Θp×Dp^. Θ_p_ is the maximum allowable length for a protein sequence. In line with prior research, protein sequences exceeding the maximum allowed length were truncated, while those falling short were padded with zeros to achieve alignment.

##### 2.5.2.4 Bilinear attention and decoder

To capture pairwise interactions, a bilinear attention network module was applied. In this model, pairwise attention weights were captured by a bilinear interaction map, followed by a bilinear pooling layer that extracted joint drug–target representations. Finally, a fully connected classification layer obtained the scores.

#### 2.5.3 Model optimization and evaluation

For each compound–protein pair, a binary activity value of 0 (possesses no anti-aging activity) or 1 (has anti-aging activity) was conducted. The main metrics used to assess model efficiency were the area under the receiver operating characteristic curve (AUROC) and the area under the precision-recall curve (AUPRC). The datasets were split randomly into 80–10–10 training–validation–testing splits for 100 epochs. Following Bai et al. (2023), we used binary cross entropy as the loss function by default. Scikit-learn was used to generate precision-recall curves for compound–protein pairs by comparing the prediction score to the activity value. Moreover, sensitivity, accuracy, and specificity were performed at the threshold associated with the optimal F1 score.

#### 2.5.4 Model prediction

As a final step, we trained the model using all the data. The model was subsequently implemented to forecast the metabolites within the HSD, along with their respective scores in relation to aging protein targets (Figure 2B). Among the numerous targets of aging, we selected the recognized targets of AMP-activated protein kinase (AMPK) and Sirt-6 (Table 1).

**TABLE 1:** Selected aging protein information.

Uniport ID	Protein	Organism
Q8N6T7	NAD-dependent protein deacylase sirtuin-6	*Homo sapiens*
Q13131	5′-AMP-activated protein kinase catalytic subunit alpha-1	
Q9Y478	5′-AMP-activated protein kinase subunit beta-1	

## 2.6 Bioactivity validation

### 2.6.1 Cell culture and viability assay

PC12 cells were cultured in high glucose DMEM under a 37°C, 5% CO_2_ atmosphere. To evaluate the cytotoxic effect of candidate metabolites on PC12 cells, five candidate metabolites at different doses were cultured with cells for 24 h. CCK-8 was used to determine cell viability.

### 2.6.2 PC12 senescence model

Senescent PC12 cells were induced by D-gal. To determine the optimal concentration of D-gal for aging induction, PC12 cells were treated with different D-gal concentrations for 6, 12, 24, and 48 h. Then, to assess the protective effect against D-gal-induced PC12 cells, five candidate metabolites were pre-treated with PC12 cells induced by D-gal for 24 h, and their viability was determined by CCK-8.

### 2.6.3 LDH, MDA, SOD, CAT, and NO measurements

The PC12 cells were treated as described above. The content of lactate dehydrogenase (LDH), malondialdehyde (MDA), superoxide dismutase (SOD), catalase (CAT), and nitric oxide (NO) were determined by commercial assay kits following the manufacturer’s protocol (Supplementary Material).

### 2.6.4 Cellular ROS concentration measurement

Reactive oxygen species (ROS) concentrations were measured with the ROS Assay Kit. Cells were seeded in 6-well plates (1 × 10^6^ cells/well) and treated with 10 μM of five candidate metabolites (ginsenoside Rg1, Rg2, and Rc, pseudoginsenoside F11, and jionoside B1) for 24 h. D-gal was then used to induce senescent cells for 24 h. The level of ROS was detected using a DCFH-DA fluorescent probe and visualized by a fluorescence microscope (Olympus U-HGLGPS, Japan).

### 2.6.5 SA-β-gal staining

Senescent PC12 cells were subjected to SA-β-gal staining. Senescent cells were observed using a fluorescence microscope (Olympus IX73, Japan). The positive rate of the senescent cells was calculated using ImageJ software.

## 3 Results

### 3.1 Identification of chemical metabolites in HSD

**FIGURE 3:**
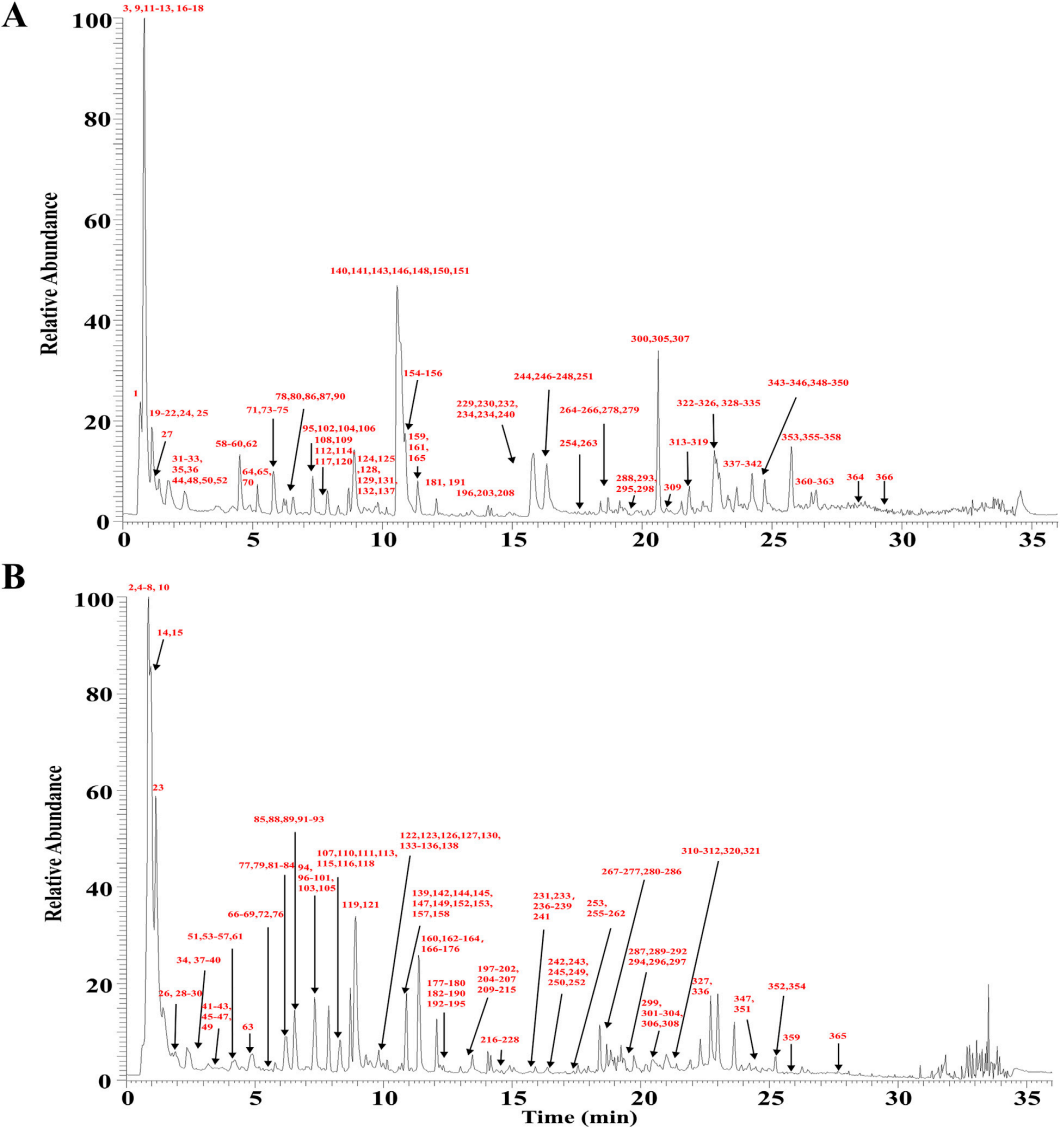
TIC of HSD from UPLC-Q Exactive-Orbitrap HRMS. **(A)** Positive ion mode. **(B)** Negative ion mode.

Based on CNKI, OTCML, Web of Science, and TCMSP, a database of HSD was established. The total ion chromatogram (TIC) in positive and negative ion modes is shown in Figure 3. After further repeated correction, 366 metabolites were identified or tentatively characterized in HSD, mainly including anthraquinones, flavonoids, alkaloids, terpenoids, organic acids, phenylethanoid glycosides, amino acids, and steroids. Among these, metabolite identification was conducted by comparison of retention time and exact mass of reference standards when standards were available. The chemical structures of other metabolites were mainly proposed by comparison with MS/MS data, accurate mass, fragmentation pathways, and relevant literature. Detailed information is summarized in Supplementary Tables S1, S2.

#### 3.1.1 Anthraquinones in HSD

In this study, 11 anthraquinones were found in HSD. To comprehensively investigate the MS/MS fragmentation pattern of anthraquinones, two reference standards—aloe-emodin (Peak 351) and emodin (Peak 352) —were unambiguously identified. Peak 352 produced precursor ions at *m/z* 269.0456 (C_15_H_10_O_5_) and generated product ions at *m/z* 241.0507 (M-H-CO)^−^, 225.0559 (M-H-CO_2_)^−^, 210.0320 (M-H-CO_2_-CH_3_)^−^, and 197.0612 (M-H-CO-CO_2_)^−^ (Supplementary Figure 1). The [M-H]^−^ ion of Peak 256 at *m/z* 283.0613 with the molecular formula C_16_H_12_O_5_ yielded the ion at *m/z* 268.0376 (M-H-CH_3_)^−^, 240.0249 (M-H-CH_3_-CO)^−^, and 212.0477 (M-H-CH_3_-2CO)^−^, which was presumed to be physcion. As such, fragment ions of [M-H-15]^−^, [M-H-44]^−^, and [M−H-28]^−^ due to the loss of CH_3_, CO_2_, and CO could be considered characteristic fragmentation behavior in the spectra of anthraquinones.

#### 3.1.2 Flavonoids in HSD

Flavonoids, a class of important polyphenolic metabolites from TCM, consist of two benzene rings (A and B) linked by three carbon atoms. In this study, 67 flavonoids were detected from HSD, including flavones, isoflavone, flavanone, flavonol, flavanonol, flavanol, and others. According to the mass spectra of the standards, the main fragment patterns of the flavonoids were as follows. First, the main MS/MS behavior of flavonoids is the breakage of the glycosidic bond and C-ring generated by RDA cleavage. In addition, the characteristic neutral loss mainly contained H_2_O, CO_2_, CO, and CH_3_ (Xu et al., 2020).

##### 3.1.2.1 Flavones in HSD

We identified 14 flavones in HSD. Four reference standards—apigenin (Peak 302), luteolin (Peak 268), cosmosiin (Peak 197), and wogonin (Peak 316) —were first ascertained. For example, Peak 197 identified [M-H]^−^ ions at 431.0988 (C_21_H_20_O_10_) and produced ions at *m/z* 269.0457 by the neutral loss of Glc. A series of ions at *m/z* 151.0040 [M-H-C_8_H_6_O]^−^, *m/z* 117.0345 [M-H-C_7_H_4_O_4_]^−^, and *m/z* 107.0135 [M-H-C_9_H_6_O_3_]^−^ were generated by RDA cleavage in the negative ion spectrum. Peak 197 was thus accurately identified as cosmosiin. Its MS^2^ mass spectra and fragmentation pathways are depicted in Supplementary Figure 2. Peak 209 displayed an [M-H]^−^ ion at *m/z* 269.0455 (C_15_H_10_O_5_). Moreover, the fragment ions at *m/z* 241.0508, *m/z* 225.0559, and *m/z* 197.0607 were generated by successive and simultaneous losses of CO and CO_2_. Based on the MS/MS data recorded by Zou et al. (2015), Peak 209 was tentatively identified as baicalein. Other detected flavones are summarized in Supplementary Table S1.

##### 3.1.2.2 Isoflavones in HSD

Nine isoflavones were identified in HSD. Peaks 125, 247, and 248 were determined to be daidzin, daidzein, and calycosin by comparison with standards. Peak 247 displayed an [M + H]^+^ ion at *m/z* 255.0652 (C_15_H_10_O_4_) and its MS^2^ spectra showed representative ions at 237.0549 (M + H-H_2_O)^+^, 227.0703 (M + H-CO)^+^, 199.0756 (M + H-2CO)^+^, and 137.0233 (M + H-C_8_H_6_O)^+^, which was considered to be daidzein. Consequently, Peaks 297, 300, 331, 340, 342, and 361 were tentatively identified as genistein, psoralenol, neobavaisoflavone, erythrinin A, corylin, and corylifol A, respectively (Chen et al., 2012; Luan et al., 2018; Wang et al., 2017).

##### 3.1.2.3 Flavanones in HSD

Ten flavanones were identified in HSD. Peaks 243 and 282 were determined to be eriodictyol and naringenin by comparing them with reference standards. Peak 282 gave a precursor ion at *m/z* 271.0613 (C_15_H_12_O_5_) in the negative mode. The fragment ion at *m/z* 151.0037(M-H-C_8_H_8_O)^−^, 119.0502 (M-H-C_7_H_4_O_4_)^−^ was observed by the RDA cleavage of the C-ring and *m/z* 177.0193 (M-H-C_6_H_6_O)^−^, 107.0139 (M-H-C_9_H_8_O_3_)^−^, so the structure of this Peak 282 was considered to be naringenin (Supplementary Figure 3).

##### 3.1.2.4 Flavonols in HSD

We identified 22 flavonols in HSD. Six reference standards—hyperoside (Peak 180), isoquercetin (Peak 181), rutin (Peak 190), astragalin (Peak 203), quercitrin (Peak 215), quercetin (Peak 271), and kaempferol (Peak 307) —were first unambiguously identified by comparison with the references. Peak 190 displayed a quasi-molecular ion at *m/z* 609.1469 (C_27_H_30_O_16_) in the negative model. MS^2^ spectra showed representative ions at *m/z* 300.0273 (M-H-Glc-Rha)^−^, 271.0250 (M-H-Glc-Rha-H-CO)^−^, 257.0457 (M-H-Glc-Rha-CO_2_)^−^, and 151.0035 (C_7_H_3_O_4_)^−^ resulting from RDA cleavage. Peak 307 displayed deprotonated ions at *m/z* 287.0550 (C_15_H_10_O_6_) and produced ions at *m/z* 269.0441 and 259.0602 by the neutral loss of H_2_O and CO. A series of representative ions at *m/z* 153.0184 and *m/z* 133.0287 were generated by RDA fragmentation, identifying Peak 307 as kaempferol. Similarly, based on similar fragmentation pathways, the structures of other peaks were identified.

##### 3.1.2.5 Flavanols in HSD

Five flavanols were identified in HSD. Peaks 77, 79, and 98 were identified as cianidanol, procyanidin B1, and epicatechin by comparison with the standards. For instance, Peaks 77 and 98 were considered to be isomers since they gave the same (M–H)^−^ ions at *m/z* 289.0720 (C_15_H_14_O_6_) and *m/z* 289.0719 (C_15_H_14_O_6_). In addition, they displayed similar fragment ions at *m/z* 245.08 (M-H-CO_2_)^−^, *m/z* 203.07 (M-H-C_3_H_2_O_3_)^−^, *m/z* 123.05 (M-H-C_8_H_6_O_4_)^−^, and 109.03 (M-H-C_9_H_8_O_4_^)−^ in the MS^2^ spectra. Based on the retention time and MS/MS behavior of the standards, they were considered to be cianidanol and epicatechin. Similarly, based on similar fragmentation pathways, Peaks 113 and 172 were tentatively identified as acetyl-epicatechin-O-glucoside and epicatechin-O-gallate.

#### 3.1.3 Alkaloids in HSD

In this study, alkaloids were tentatively identified, including tetrahydroprotoberberine, protoberberine, aporphine, benzylisoquinoline, indole, quinoline, and other alkaloids. By comparing retention time and MS/MS behavior with reference standards, Peaks 3, 58, 64, 128, 132, 150, 151, and 155 were identified as betaine, phellodendrine, magnoflorine, columbamine, jateorhizine, epiberberine, coptisine, and berberine.

Phellodendrine, the main tetrahydroprotoberberine alkaloid in HSD, showed the quasi-molecular ion [M + H]^+^ at *m/z* 342.1699 (C_20_H_24_NO_4_) in positive ion mode, and the main fragment ions were observed at *m/z* 192.1021 (M-C_9_H_10_O_2_)^+^ and *m/z* 177.0785 (M-C_9_H_10_O_2_-CH_3_)^+^ by retro Diels–Alder (RDA) fragmentation of the C-ring (Supplementary Figure 4). It displayed the (M + H)^+^ ions at *m/z* 356.1858 (C_21_H_25_NO_4_), and the main fragment ions were 341.1617 [M + H-CH_3_]^+^, 192.1021 [M + H-C_9_H_10_O_2_]^+^, 177.0785 [M + H-C_9_H_10_O_2_-CH_3_]^+^, and 165.0914 [M + H-C_11_H_13_NO_2_]^+^. Based on MS/MS fragmentation patterns and the literature, Peak 104 was tentatively identified as yuanhunine. As such, tetrahydroprotoberberine alkaloids possess a characteristic fragmentation behavior with RDA cleavage and successive loss of substituent groups from the parent nucleus, such as CH_3_ and OCH_3_.

In addition, the MS/MS behavior of protoberberine alkaloids was characterized by the simultaneous or successive loss of substituent groups like CO, CH_4,_ and CH_3_. For instance, Peaks 150 and 155 exhibited similar (M + H)^+^ ions at *m/z* 336.1230 (C_20_H_18_NO_4_) and 336.1229 (C_20_H_18_NO_4_). Additionally, they shared the same fragment ions at *m/z* 320.09 (M-CH_4_)^+^, 306.08 (M-2CH_3_)^+^, 292.10 (M-CH_4_-CO)^+^, and 278.08 (M-2CH_3_-CO)^+^. Based on the retention time and fragmentation patterns of the reference standards, they were accurately identified as epiberberine and berberine. MS^2^ mass spectra and the fragmentation pathways of berberine are depicted in Supplementary Figure 5. Peak 146 gave an [M + H]^+^ ion at *m/z* 322.1075 with a molecular formula of C_19_H_15_NO_4_. The predominant fragment ions appeared at 307.0841 (M + H-CH_3_)^+^, 292.0593 (M + H-2CH_3_)^+^, 279.0891 (M + H-CH_3_-CO)^+^, 264.0665 (M + H-2CH_3_-CO)^+^, and 251.0947 (M + H-CH_3_-CO-CO)^+^, consisting of the fragment patterns of protoberberine alkaloids. Thus, it was tentatively identified as berberrubine.

For aporphine alkaloids, the primary fragmentation pattern was the successive loss of (CH_3_)_2_NH or CH_3_NH_2_. Magnoflorine was taken as an example, giving the quasi-molecular ion [M + H]^+^ at *m/z* 342.1700 (C_20_H_24_NO_4_) and fragment ions at 297.1123 (M-(CH_3_)_2_NH)^+^, 282.0882 (M-(CH_3_)_2_NH-CH_3_)^+^, 265.0859 (M-(CH_3_)_2_NH-CH_3_OH)^+^, and 237.0906 (M-(CH_3_)_2_NH-CH_3_OH-CO)^+^ in positive ion mode. Their MS^2^ mass spectra and the fragmentation pathways are depicted in Supplementary Figure 6. Similarly, Peaks 74 and 78 were tentatively identified as cassythidine and dauricine based on fragment patterns.

As documented in the literature, the main fragment patterns of the benzylisoquinoline alkaloids were as follows. Benzylisoquinoline alkaloids, with isoquinoline or tetrahydroisoquinoline as the parent nucleus, are a class of alkaloids with a benzyl group attached to the 1-position of the nucleus. Therefore, they are susceptible to displaying α-cleavage. Moreover, the loss of neutral fragments, such as CH_3_OH, OCH_3,_ and (CH_3_)_2_NH, are also the main fragment patterns. Peak 48 gave (M)^+^ ions at *m/z* 314.1751 (C_19_H_24_NO_3_) and (M-C_12_H_16_NO_2_)^+^ ions at *m/z* 107.0496 by α-cleavage. The fragment ions at *m/z* 269.1173 (M-(CH_3_)_2_NH)^+^ and 237.0909 (M-(CH_3_)_2_NH-CH_3_OH)^+^ were further obtained, indicating that Peak 48 was presumed to be magnocurarine. Accordingly, other peaks were tentatively identified as higenamine, lotusine, sanjoinine K, N-methylhigenamine7-glucopyranoside, oblongine, 3,4-dihydro-1-[(4-hydroxyphenyl)-methyl]-7-methoxy-2-methyl-6-isoquinolinol, and tembetarine (Sun et al., 2016; Xian et al., 2014; Yang et al., 2021).

#### 3.1.4 Terpenoids in HSD

A total of 57 terpenoids were detected, containing 13 iridoids, one sesquiterpenoid, 39 triterpenoids, and their derivatives. In order to comprehensively investigate the MS/MS fragmentation pattern of iridoids, two reference standards—rehmannioside D (Peak 26) and geniposidic acid (Peak 34) —were first characterized. The MS^2^ mass spectra and the fragmentation pathways of geniposidic acid are depicted in Supplementary Figure 7. The primary fragmentation pattern of iridoids was the loss of glycosidic units from the parent nucleus, such as glucose (Glc) and rhamonose (Rha). Then, the residual aglycone ions tended to easily lose CO_2_ or H_2_O because of hydroxyl and carboxyl groups. For instance, Peak 34 provided the intense deprotonated ion at *m/z* 373.1141 (C_16_H_22_O_10_), and generated product ions 211.0612 (M-H-Glc)^−^ and 167.0713 (M-H-Glc-CO_2_)^−^ resulting from decarboxylation, 149.0607 (M-H-Glc-CO_2_-H_2_O)^−^ from the condensation reaction of hydroxyl and protons, and 123.0451 (M-H-Glc-CO_2_-H_2_O-C_2_H_2_)^−^. Fragment ions of (M-H-CO_2_)^−^ and (M-H-H_2_O)^−^ could be considered as the characteristic ions of iridoids. Therefore, other peaks were tentatively identified as iridoids based on the above proposed fragmentation pathway (Li et al., 2017; Song et al., 2016; Wang et al., 2013; Zhu et al., 2014).

Triterpenoids were identified in HSD. Taking Peaks 249 and 252 as examples, both provided a precursor ion at *m/z* 485.1823 (C_26_H_30_O_9_) and 485.1820(C_26_H_30_O_9_) and generated the same product ions at *m/z* 345.17 (M-H-C_6_H_4_O_4_)^−^, 317.18 (M-H-C_7_H_4_O_5_)^−^, 205.09 (M-H-C_14_H_16_O_6_)^−^, 161.10 (M-H-C_15_H_16_O_8_]^−^, and 129.06 (M-H-C_20_H_20_O_6_)^−^. Based on MS/MS fragmentation patterns and the literature, they were tentatively identified as rutaevine and its isomer. Peaks 254, 293, and 319 were tentatively identified as oleanolic acid, limonin, and obacunone from the literature.

We identified 34 triterpenoid saponins in HSD. The majority of these metabolites are found in ginsenosides. Therefore, to investigate the MS/MS fragmentation pattern of triterpenoid saponins, three reference standards of ginsenoside Rb1 (Peak 270), ginsenoside Re (Peak 218), and ginsenoside Rg1 (Peak 216) were precisely identified. Peak 218 gave an [M-H]^−^ ion at 945.5422 (C_48_H_82_O_18_) and produced fragment ions at *m/z* 783.4907 (M-H-Glc)^-^, 637.4339 (M-H-Glc-Rha)^−^, and 475.3798 (M-H-2Glc-Rha)^−^, which was consistent with the fragment patterns of the reference standards and was identified as ginsenoside Re (Supplementary Figure 8). Peak 216 observed an [M + COOH]^−^ adduct ion at *m/z* 845.4920 (C_43_H_73_O_16_) and an [M-H]^−^ ion at *m/z* 799.4860 (C_42_H_72_O_14_). Predominant fragment ions at the MS^2^ spectrum were 637.4330 (M-H-Glc)^−^, 475.3802 (M-H-2Glc)^−^, and 391.2874 (M-H-2Glc-C_6_H_12_)^−^, respectively. Consequently, Peak 216 was identified as ginsenoside Rg1 by comparison with a standard.

Peak 296 produced an [M-H]^−^ ion at 925.4816 (C_47_H_74_O_18_). The characteristic fragment ions were 763.4269 (M-H-Glc)^−^, 701.4291 (M-H-Glc-H_2_O-CO_2_)^−^, 595.3568 (M-H-Glc-H_2_O-Ara-H_2_O)^−^, 551.3733 (M-H-Glc-H_2_O-Ara-H_2_O-CO_2_)^−^, and 455.3524 (M-H-Glc-Ara-Glua)^−^, suggesting that it was chikusetsusaponin IV. Other triterpenoid saponins exhibited similar fragment patterns; detailed information is summarized in Supplementary Tables S1, S2 (Jinbiao et al., 2022; Li et al., 2010; Lin et al., 2021; Tang et al., 2015; Wang et al., 2013).

#### 3.1.5 Coumarins and lignans in HSD

In this study, 27 lignans were identified in HSD in positive ion mode. To comprehensively explore MS/MS characteristic dissociation rules of lignans, six reference standards—gomisin J (Peak 324), schisandrin B (Peak 363), schisandrol B (Peak 317), schizandrin A (Peak 355), schisandrol A (Peak 305), and angeloyl gomisin H (Peak 334) —were used. First, because of the different substitutions on the biphenyl ring, the fragment ions [M-H-15]^−^, [M-H-18]^−^, [M-H-30]^−^, and [M-H-31]^−^ corresponding to the loss of CH_3_, H_2_O, CH_2_O, and OCH_3_ could be identified as characteristic ions of lignans. Moreover, the loss of neutral fragments such as C_4_H_8_ and C_3_H_6_ generated by biphenyl ring opening was also observed in positive ion mode. In this mode, several main fragment ions at *m/z* 417.2273 (C_24_H_32_O_6_), 402.2037 (M + H-CH_3_)^+^, 386.2092 (M + H-CH_3_-O)^+^, 347.1488 (M + H-CH_3_-C_4_H_7_)^+^, 316.1307 (M + H-CH_3_-O-C_4_H_8_-CH_2_)^+^, and 301.1072 (M + H-CH_3_-O-C_4_H_8_-CH_2_-CH_3_)^+^ were obtained in the MS^2^ spectrum of Peak 355. By comparing the retention time and fragment patterns with those of the standards, Peak 355 was precisely identified as schizandrin A (Supplementary Figure 9). Moreover, MS^2^ mass spectra and the fragmentation pathways of schisandrol A are depicted in Supplementary Figure 10. Similarly, via the above fragment rules, other peaks were identified (Wang et al., 2020; Yang et al., 2017; Zou et al., 2015).

In addition, ten coumarins were identified in HSD. The characteristic fragmentation pattern of the coumarins was the consecutive loss of CO and CO_2_ due to the presence of a lactone. Moreover, it is also noteworthy that coumarins displayed intense molecular ion peaks in the mass spectra. For instance, Peaks 240 and 246 were assigned as isomers since they gave the same (M + H)^+^ ions at *m/z* 187.0390 with the molecular formula C_11_H_6_O_3_. Both then displayed similar fragment ions at *m/z* 159.04 (M-H-CO)^−^, 143.05 (M-H-CO_2_)^−^, 131.05 (M-H-2CO)^−^, and 115.05 (M-H-CO-CO_2_)^−^. Therefore, by comparing the retention time and fragment ions of the standards, they were exactly determined to be angelicin and psoralen (Supplementary Figure 11). Based on similar mass spectra profiles, other coumarins are tentatively identified and summarized in Supplementary Tables S1 and S2.

#### 3.1.6 Organic acids in HSD

In this study, 58 organic acids were detected, including aliphatic organic acids, phenolic acid derivatives, quinic acid, and derivatives. In order to explore the fragmentation pathway of quinic acid and derivatives, four reference standards—1,5-dicaffeoylquinic acid (Peak 204), chlorogenic acid (Peak 85), neochlorogenic acid (Peak 53), and cryptochlorogenic acid (Peak 82) —were unambiguously identified. Taking chlorogenic acid as an example, Peaks 53, 82, and 88 were isomers and gave an [M-H]^-^ ion at *m/z* 353.0881, 353.0880, and 353.0880 with a molecular formula of C_16_H_18_O_9_. The MS/MS spectrum also showed the same ion at *m/z* 191.06 (M-H-caffeoyl)^−^, 179.04 (M-H-C_7_H_10_O_5_)^−^, 173.05 (M-H-C_9_H_8_O_4_)^−^, 135.05 (M-H-C_7_H_10_O_5_-CO_2_)^−^, and 93.03 (M-H-C_9_H_8_O_4_-2H_2_O-CO_2_)^−^. Therefore, three isomers were sequentially detected by comparison with standards. MS^2^ mass spectra and the fragmentation pathways of chlorogenic acid are depicted in Supplementary Figure 12. According to the mass spectra of the above standards, the main fragment patterns of quinic acid and its derivatives are as follows. First, the fragment ions were mainly generated through the breakage of ester and glycosidic bonds. Moreover, fragment ions such as (M-H-C_10_H_8_O_3_), (M-H-C_9_H_6_O_3_), (M-H-C_6_H_10_O_5_), (M-H-H_2_O), and (M-H-CO_2_) could be regarded as the characteristic ions. Consequently, the remaining peaks were tentatively identified using Sun et al. (2016), Wang et al. (2021), and Zhang et al. (2018).

As for the phenolic acid derivatives, their characteristic fragmentation involves successive losses of neutral groups such as CO_2_, H_2_O, CH_3_, and Glc. Peaks 68, 28, 94, and 142 were unequivocally characterized as phthalic, gallic, caffeic, and ferulic acids by contrast with reference standards. Peak 28, for example, displayed a quasi-molecular ion at *m/z* 169.0142 (C_7_H_6_O_5_) and representative ions at *m/z* 125.0244(M-H-CO_2_)^-^, 97.0295(M-H-C_3_H_4_O_3_)^−^, and 69.0347(M-H-CO_2_-2CO)^−^. Peak 142 gave an [M-H]^−^ ion at *m/z* 193.0505 with a molecular formula C_10_H_10_O_4_, and predominant ions appeared at *m/z* 178.0271 (M-H-CH_3_)^−^, 149.0608 (M-H-CO_2_)^−^, and 134.0374 (M-H-CO_2_-CH_3_)^−^, which were deemed to be ferulic acid. Detailed information on other metabolites is summarized in Supplementary Tables S1 and S2.

For aliphatic organic acids like phenolic acid, (M-H-CO_2_)^−^, (M-H-COOH)^−^, and (M-H-H_2_O)^−^ were identified as characteristic ions. Peak 171 has a precursor ion at *m/z* 187.0975 (C_9_H_16_O_4_) and generated product ions *m/z* 169.0870 (M-H-H_2_O)^-^, 143.1077 (M-H-CO_2_)^−^, and 125.0972 (M-H-H_2_O-COOH)^−^. Based on exact molecular masses and MS/MS fragmentation patterns in the literature, Peak 171 was tentatively identified as azelaic acid. Similarly, Peaks 263, 327, 279, and 320 were tentatively identified as palmitic acid, decanoic acid, 10,12-octadecanedioic acid, and 9-Hpode (Xie et al., 2023).

#### 3.1.7 Phenylethanoid glycosides in HSD

This study detected 36 phenylethanoid glycosides in negative ion mode. Peak 116, for example, yielded [M-H]^−^ at *m/z* 785.2524 (C_35_H_46_O_20_), and predominant ions appeared at *m/z* 623.2195 (M-H-caffeoyl)^−^, 477.1642 (M-H-caffeoyl-Rha)^−^, and 315.1101 (M-H-caffeoyl-Rha-Glc)^−^. By comparing their chromatographic retention times and fragmentation patterns with those of the standards, Peak 116 was exactly identified as echinacoside, a representative metabolite of phenylethanoid glycosides (Supplementary Figure 13). By analyzing the structures of phenylethanoid glycosides and the literature, fragment ions of [M-H-146]^−^, [M-H-162]^−^ and [M−H-18]^−^ due to the loss of C_6_H_10_O_4_ (rhamnose residue), C_9_H_6_O_3_ or C_6_H_10_O_5_ (glucose residue, caffeoyl group), and H_2_O could be regarded as diagnostic cracking pathways in the spectra of phenylethanoid glycosides. A similar fragment pattern was observed in Peaks 152 and 158, which gave [M-H]^−^ at *m/z* 827.2631(C_37_H_48_O_21_) and 623.1994 (C_29_H_36_O_15_), respectively. In the MS/MS spectrum, Peak 162 showed the ion at *m/z* 665.2318 (M-H-caffeoyl)^−^, 623.2224 (M-H-caffeoyl-C_2_H_2_O)^−^, and 477.1624 (M-H-caffeoyl-C_2_H_2_O-Glc)^−^, and Peak 168 showed the ion at *m/z* 461.1646 (M-H-caffeoyl)^−^, 315.1086 (M-H-caffeoyl-Rha)^−^, 179.0348 (Caffeoyl-H)^−^, 161.0244 (Caffeoyl-H-H_2_0)^−^, and 135.0452 (M-H-caffeoyl-Rha-Glc-H_2_O)^−^. According to the cracking patterns obtained, Peaks 152 and 158 were identified as tubuloside A and acteoside. Detailed information is summarized in Supplementary Tables S1 and S2 (Han et al., 2012; Li et al., 2015; Song et al., 2016).

#### 3.1.8 Amino acids in HSD

Eight Amino acids were identified in HSD in positive ion mode. Phenylalanine (Peak 27) was first identified to explore the fragmentation pathway. By comparison with MS/MS fragmentation of the standards, the characteristic fragmentation was successive losses of COOH and NH_3_. Peak 27 gave an [M + H]^+^ ion at *m/z* 166.0864 (C_9_H_11_NO_2_) and further yielded the ion at *m/z* 149.0594 [M+H-NH_3_]^+^, 120.0810 [M+H-COOH]^+^, 103.0546[M+H-NH_3_-COOH]^+^ suggesting that it should be phenylalanine. Its fragmentation pathways are depicted in Supplementary Figure 14. Similarly, other peaks were tentatively identified as arginine (Peak 1), adenine (Peak 9), proline (Peak 16), L-pyroglutamic acid (Peak 20), leucine (Peak 21), tyrosine (Peak 22), and tryptophan (Peak 33).

#### 3.1.9 Steroids in HSD

Seven steroids were identified in HSD. Peak 141 exhibited a quasi-molecular ion [M + H]^+^ at *m/z* 481.3162 (C_27_H_44_O_7_) and produced ions at 463.3061 (M + H-H_2_0)^+^, 371.2219 (M + H-C_4_H_14_O_3_)^+^, 319.1910 (M + H-C_8_H_18_O_3_)^+^, and 301.1805 (M + H-C_8_H_18_O_3_-H_2_O)^+^, which were considered to be β-ecdysterone by comparing retention times and MS/MS spectra with those of the reference standards. Similarly, other peaks were tentatively identified as polypodine B (Peak 115), stachysterone C (Peak 140), 25R-achyranthes bidentata (Peak 149), 25S-achyranthes bidentata (Peak 153), 25R achyranthes dioscin (Peak 255), and cyasterone (Peak 298) (Ma et al., 2021; Yang et al., 2021).

#### 3.1.10 Other metabolites in HSD

Metabolites other than those mentioned above were also detected, such as phthalides, nucleosides, and stilbenes. There were three nucleosides identified in HSD. For example, Peak 19 was unambiguously identified as adenosine by comparison with a reference standard, giving precursor ions [M + H]^+^ at *m/z* 268.1039 (C_10_H_13_N_5_O_4_) and formed ions at *m/z* 136.0619 [M + H-C_5_H_8_O_4_]^+^ and 119.0356 [M + H-C_5_H_8_O_4_-NH_3_]^+^. Peak 166 (tetrahydroxystilbene glucoside) displayed a precursor ion [M-H]^−^ at *m/z* 405.1194 (C_20_H_22_O_9_) and yielded the ion at *m/z* 243.0663 (M-H-Glc)^−^, 225.0559 (M-H-Glc-H_2_O)^−^, 215.0714 (M-H-Glc-CO)^−^, 137.0244 (M-H-Glc-C_7_H_5_O)^−^, and 93.0346 (M-H-Glc-C_7_H_5_O-CO)^−^.

For the phthalides, two reference standards—ligustilide (Peak 333) and levistilide A (Peak 362) —were identified. Levistilide A produced a deprotonated molecular ion at *m/z* 381.2060 (C_24_H_28_O_4_) in the positive ion mode and diagnostic ions at *m/z* 191.1068 (M + H-C_12_H_14_O_2_)^+^, 173.0962 (M + H-C_12_H_14_O_2_-H_2_O)^+^, 149.0598 (M + H-C_12_H_14_O_2_-C_3_H_6_)^+^, and 135.0441 (M + H-C_12_H_14_O_2_-C_4_H_8_)^+^(Supplementary Figure 15). Due to the ring-opening reaction, the fragment ions at *m/z* [M + H-H_2_O]^+^ and [M + H-CO]^+^ could be regarded as characteristic. Based on the similar fragmentation pathways, other peaks were identified as senkyunolide J (Peak 95), 4,7-dihydroxy-3-butylphthalide (Peak 143), senkyunolide F (Peak 161), butylidene phthalide (Peak 232), senkyunolide (Peak 235), butylphthalide (Peak 266), senkyunolide A (Peak 309), and senkyunolide P (peak 353). Detailed information on other peaks is summarized in Supplementary Tables S1 and S2 (Liu et al., 2009; Wang et al., 2023; Zhang et al., 2014; Zhu et al., 2014).

### 3.2 Sequential metabolism experiments of HSD

#### 3.2.1 Stability in artificial gastric juice

Artificial gastric juice stability is a crucial determinant of the subsequent potency and efficacy of the composition since metabolites with high lipophilicity or low solubility could be degraded by metabolic enzymes in the stomach after oral administration. The stability results were obtained by comparing the MS/MS spectra before and after incubation. Notably, 178 peaks were identified from the sample after incubation, indicating that HSD was found to be quite stable upon digestion of gastric juice (Supplementary Table S3). It is also an essential prerequisite for pharmacodynamic studies.

#### 3.2.2 Identification of absorbed metabolites

Metabolites are mostly absorbed, metabolized in the small intestine and liver, and excreted in the urine and feces after passing through the stomach. In this study, in order to obtain a comprehensive dynamic process of HSD, different but complementary surgical methods were performed to collect plasma samples (intestinal metabolism, liver metabolism, and systemic metabolism), urine samples, and fecal samples. The results showed that 135 metabolites were absorbed into plasma, indicating that they could be potential functional metabolites. In addition, 102 peaks were from the fecal sample and 91 were from the urine sample. Detailed information is summarized in Supplementary Table S3.

### 3.3 Deep learning model analysis

#### 3.3.1 Validation of the deep learning model

**FIGURE 4:**
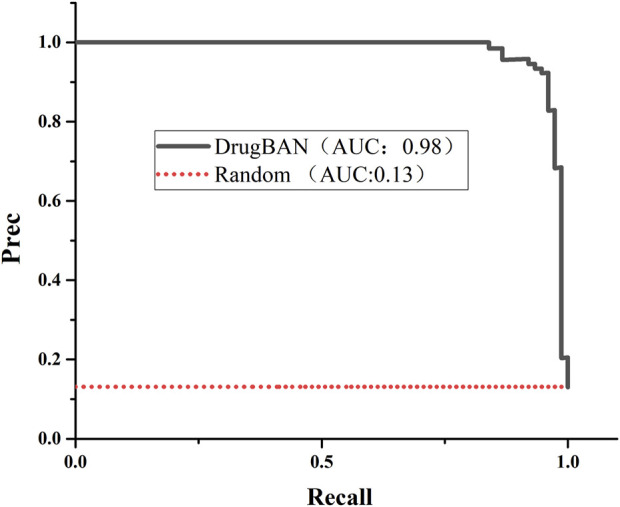
Precision–recall curve of the trained DrugBAN and the initial random model.

We used the drugBAN model to predict senolytic activities; the code is available on GitHub (Liu et al., 2023). We trained the model using our collected senolytic dataset by training and testing on 80–20 splits. A value of 0.9772 was found for auPRC, which measures the model’s ability to correctly identify a senolytic compound. As a result, the model could identify senolytic metabolites in our training set more accurately than a random initial model (auPRC of 0.1313). Figure 4 shows the precision-recall curve. Different benchmarks of model performance, such as AUROC and F1 score, indicated better performance for the DrugBAN model (Table 2).

**TABLE 2:** Model performance.

Metric	Trained model	Random initial model
AUROC	0.9901	0.5450
AUPRC	0.9772	0.1313
F1 score	0.9729	—
Sensitivity	0.9865	—
Specificity	0.9600	—
Accuracy	0.9832	—
Threshold	0.2706	—

#### 3.3.2 Prediction of deep learning model

Our model performed well, so we retrained it using our entire dataset to predict the senolytic activities of metabolites in HSD. We selected several different aging protein targets to pair with each metabolite in HSD and predicted the interaction score. The metabolites exhibited a range of prediction scores, from 0.08 to 1, indicating that our model is capable of discriminating between actives and inactives. Detailed information on the top 40 is summarized in Supplementary Table S4.

### 3.4 Bioactivity validation

#### 3.4.1 Candidate metabolites improve the viability of D-gal-induced PC12 cells

**FIGURE 5:**
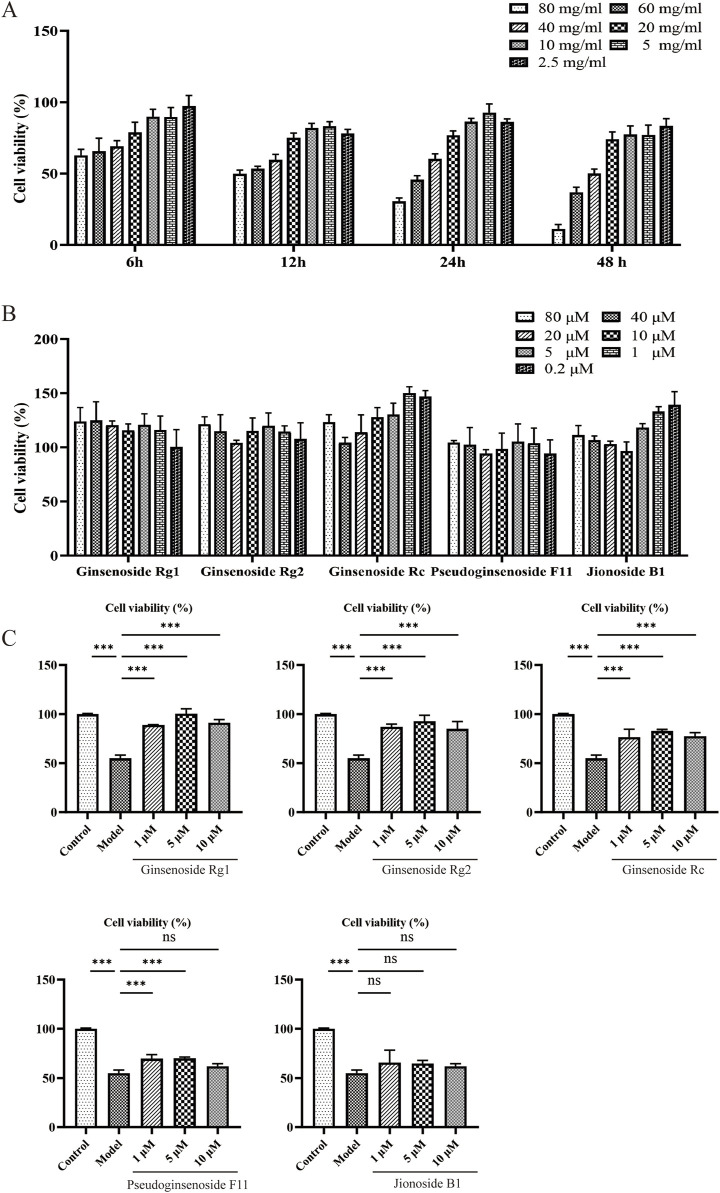
Effects of candidates on D-gal-induced PC12 cell senescence. **(A)** Impact of D-gal treatments on PC12 cells for 6, 12, 24, and 48 h. **(B)** Cell viability of PC12 cells treated with candidates. **(C)** Cell viability of senescence PC12 cells treated with candidates.

We used the CCK8 method to assess the cytotoxicity and protective effects of five metabolites on senescent PC12 cells. In Figure 5B, no significant cytotoxicity was recorded at various concentrations. Proliferative effects on PC12 cells in a dose-dependent manner were observed, especially for ginsenoside Rc and jionoside B1.

High levels of D-gal could generate oxidative stress by producing ROS and accelerate cell aging. A model of aging based on D-gal is a widely used anti-aging study; we used cells induced by D-gal to study the protective effect of metabolites. As shown in Figure 5A, 40, 60, and 80 mg/mL of D-gal significantly reduced cell viability compared with the control group in a dose-dependent manner. Of note, 60 mg/mL of D-gal decreased cell viability by 45.81% within 24 h (p < 0.01). This concentration was chosen for further investigation. As shown in Figure 5C, compared with the model group, the candidate metabolites could protect cells from D-gal-induced decrease in cell viability. Jionoside B1, although the degree is not statistically significant, also showed some extent of protective effect (approximately 62.07–65.72%).

#### 3.4.2 Impact of candidate metabolites on D-gal-induced oxidative stress in PC12 cells

**FIGURE 6:**
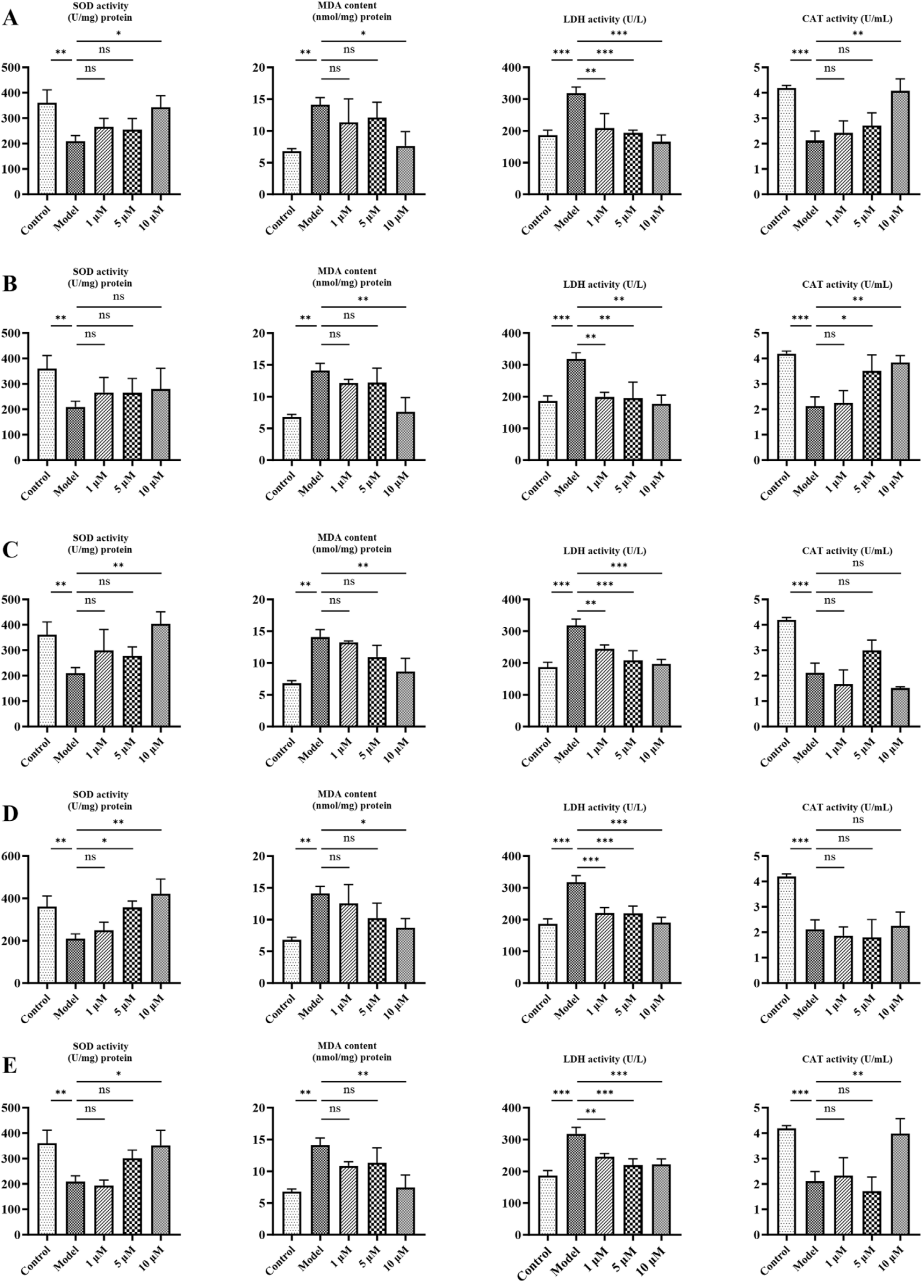
Effects of candidates on oxidative stress indicators (SOD, LDH, CAT, and MDA) in PC12 cells. **(A)** Ginsenoside Rg1. **(B)** Ginsenoside Rg2. **(C)** Ginsenoside Rc. **(D)** Pseudoginsenoside F11. **(E)** Jionoside B1. Data are presented as mean ± SD from each group. *p < 0.05, **p < 0.01, and ***p < 0.001 were contrasted with control and model groups.

It is well-accepted that aging is associated with oxidative stress—a pivotal mechanism that drives cell senescence. Therefore, the markers of oxidative stress—LDH, MDA, SOD, and CAT—were determined in the aging cell model. As presented in Figure 6, the model group increased the production of MDA and LDH and decreased the activities of SOD and CAT compared with the control group (p < 0.01). However, LDH and MDA levels were dose-dependently reduced after treatment with metabolites (p < 0.05). The antioxidant activity of SOD and CAT was also increased compared to the model (p < 0.05). Overall, these results indicate that metabolites significantly alleviate oxidative stress in cellular aging models.

#### 3.4.3 ROS measurement and SA-β-gal staining results

**FIGURE 7:**
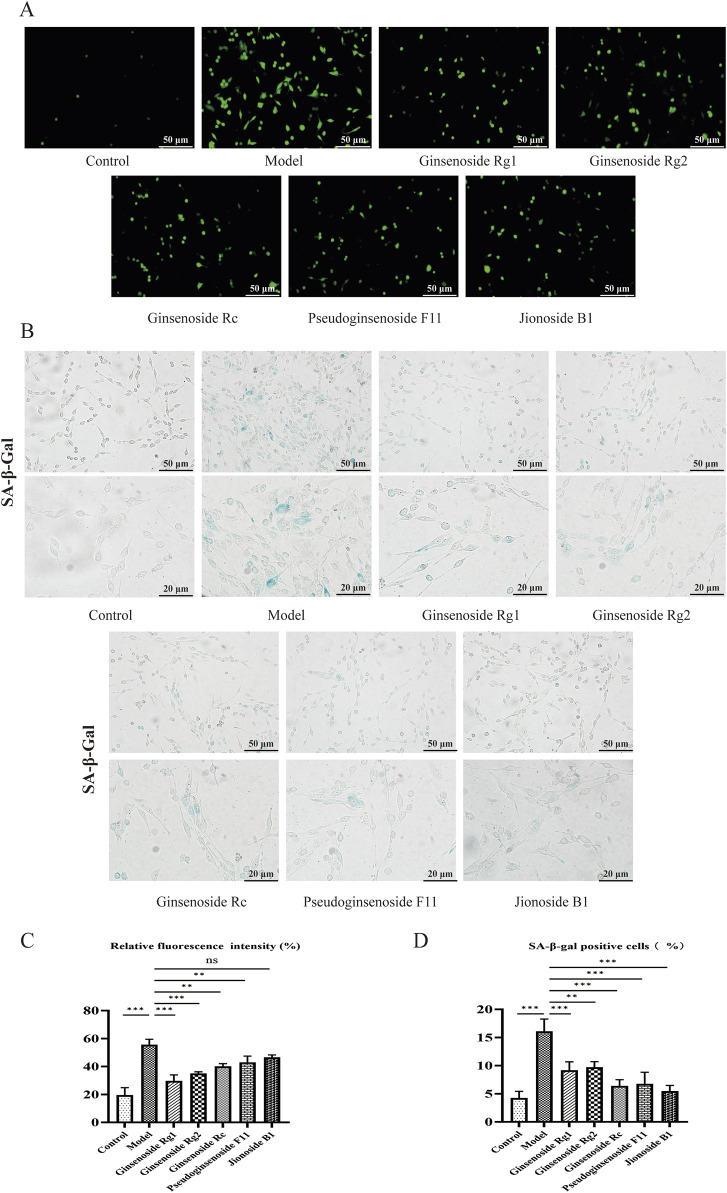
Effects of candidates on the ROS level and cell senescence in PC12 cells. **(A)** Representative photographs of ROS levels in PC12 cells, Scale bar = 50 μm. **(B)** Typical photographs of SA-β-gal-stained PC12 cells. Scale bar = 50, 20 μm. **(C)** Mean fluorescence intensity of the ROS level. **(D)** Ratio of SA-β-gal-stained cells. Data are presented as mean ± SD from each group. *p < 0.05, **p < 0.01, and ***p < 0.001 were contrasted with control and model groups.

Given that increased ROS levels could induce senescence, we performed a ROS assay to investigate the effects of metabolites on the level of cellular ROS. As shown in Figures 7A and C, compared with the control group the model group showed a 2.72-fold increase in ROS content in senescent PC12 cells (p < 0.001). Notably, pretreatment with 10 μM of metabolites could considerably decrease ROS (p < 0.01), with the exception of jionoside B1.

A biomarker of cellular senescence is the upregulation of SA-β-gal activity. SA-β-gal staining demonstrated that pretreatment with metabolites could significantly decrease the proportion of SA-β-gal-positive cells. As demonstrated in Figures 7B and D, 60 mg/mL of D-gal significantly induced senescence and increased the rate of SA-β-gal-stained cells (p< 0.001), while administration of D-gal markedly decreased the SA-β-gal staining rate by 5.48–9.72% (p < 0.01).

## 4 Discussion

Aging, a natural and complex life process, is primarily marked by the gradual and inevitable deterioration of cells, tissues, and organs, leading to impaired function and heightened risk of mortality. The United Nations has predicted that by 2050, one in six people worldwide will be 65 years of age or older (Cai et al., 2022). Aging constitutes a significant risk factor for various diseases, including metabolic, cardiovascular, and neurodegenerative conditions (Kennerdell et al., 2018). Both the public and academic community have shown significant interest in aging.

Although the molecular mechanisms underlying aging remain a mystery, the free radical theory of aging (FRTA) has long been one of the most influential theories for elucidating the mechanisms underlying the aging process. According to FRTA, the accumulation of oxidative damage caused by excessive ROS, byproducts of normal aerobic metabolism, is among the primary contributors to aging (Bratic and Larsson, 2013; Harman et al., 1956). In summary, an imbalance between the generation and elimination of ROS leads to severe oxidative damage in various cell types, accelerating the aging process. As a common nutrient and reducing sugar, D-galactose is frequently used to establish aging models *in vitro* and *in vivo*. Normally, D-gal can be metabolized into glucose by the liver. However, at higher concentrations, it is converted to aldose and hydroperoxide by galactose oxidase, leading to the generation of ROS, changes in cell osmotic pressure, cell swelling, and membrane lipid damage (signs of aging) (Azman and Zakaria, 2019). Additionally, D-gal also could induce protein glycation reactions, ultimately forming advanced glycation end products (AGEs), the accumulation of which is a common feature of many age-related neurodegenerative diseases. Thus, PC12 senescent cells induced by D-gal were selected as a model for an activity assay.

AMPK is a highly conserved intracellular energy sensor present in all eukaryotic organisms, and it plays a pivotal role in cell senescence, oxidative stress, and inflammatory response. Several studies have demonstrated that activation or overexpression of AMPK can extend the lifespan of *C. elegans*, nematodes, and aging mice. Notably, AMPK has been found to activate peroxisome proliferator-activated receptor-gamma coactivator, resulting in the expression of scavenging enzymes (SOD, CAT) and an extended lifespan (González et al., 2020). Similarly, Sirt6, a NAD + -dependent histone deacetylase, had similar protective effects in oxidative stress and regulating longevity (López-Otín et al., 2013). Consequently, a deep learning model was employed to screen five candidates based on their interaction with AMPK and Sirt6.

SOD and CAT are pivotal antioxidant enzymes that maintain a normal antioxidant–oxidant balance in the defense system, which could convert superoxide to water and oxygen (Olson et al., 2018). MDA is a common product of unsaturated lipid degradation caused by ROS and is considered a marker of aging (Zhang et al., 2022). In this study, PC12 cells induced by D-gal exhibited decreased SOD and CAT activity, along with increased MDA levels. Nevertheless, treatment with candidate metabolites resulted in significant antioxidant activity. The SA-β-gal staining experiment showed that the number of positive cells was significantly lower than in the model group. The intracellular ROS level also exhibited similar results. In addition, we measured the release of LDH and NO content. The activity of LDH was also decreased after incubation with metabolites, indicating the protective effect on intact membranes. However, in the NO assay, although there was a slight downward trend was observed between the model and treatment groups, it was not statistically significant (Supplementary Figure 18).

We also conducted metabolic sites based on sequential metabolism experiments. In this study, we collected and tested six samples of HSD both *in vitro* and *in vivo*, including gastric (metabolized by artificial gastric juice), intestinal (metabolized by intestinal enzymes), liver (metabolized by intestinal enzymes and liver), systemic sample (oral route of administration), urine, and fecal samples. Compared to identifying metabolites solely based on oral administration (i.e., a systemic sample), accurate identification of metabolic sites could provide a comprehensive understanding of the metabolic profile, thus avoiding omission of metabolites due to low oral bioavailability or content. At the same time, it could serve to optimize dosage form and prodrug design.

The result of the *in vitro* stability experiment showed that all metabolites were essentially stable and resistant to digestion in the artificial gastric juice except for ginsenoside Rg2. However, only ginsenoside Rg1 and Rc were detected in liver samples due to the liver metabolism. No metabolites were detected in the systemic sample, which may be attributable to the influence of intestinal microflora or relatively low content. Interestingly, all metabolites were detectable in the intestinal sample. This suggests that the five candidates could be absorbed by small intestinal epithelial cells into the bloodstream. Typically, multiple metabolites have been characterized in many cases as undetectable, as they were completely metabolized. However, limited detection capacity also plays a significant role. Sequential metabolism experiments allowed us to mitigate false negative results. Consequently, five candidates were selected for the efficacy study. In this study, HSD was studied in detail for the first time, especially concerning metabolic sites. Therefore, under the guidance of the 4R rules, we conducted a single-dose investigation, as this study aligns with the characteristics of the early-stage exploratory study. The early exploratory research phase is characterized by initial exploration and understanding of new fields. This study provided a preliminary assessment of the material basis of HSD, which could offer guidance for subsequent in-depth research. More importantly, by following these rules, the use of single-dose investigations also allowed us to reduce the number of animals through an efficient experimental design, demonstrating our commitment to ethical responsibility, methodological precision, and effective research.

The complexity and variability of metabolites are hallmarks of TCM (Li et al., 2016). Therefore, an efficient screening strategy is useful to find metabolites from TCM. In the present study, a deep learning model was employed to autonomously select five candidate metabolites. Several other metabolites, including achyranthoside D and C and ginsenoside F2, were also present. However, these were not chosen for further study due to their pharmacokinetic profiles, partly influenced by extensive metabolism *in vivo*. Nevertheless, innovative design, synthesis of prodrugs, and drug delivery systems offer promising avenues for the discovery of bioactive small molecules. Given that TCM is a multi-component system, it is also of interest to explore the synergistic effects of its metabolites, particularly in addressing aging, which is influenced by complex mechanisms (Sun et al., 2019).

In this study, we employed a deep learning model to predict potential anti-aging metabolites in HSD for the first time. Our deep learning analysis yielded valuable biological insights by accurately predicting the anti-aging activity of metabolites identified in our research. Specifically, the model’s ability to accurately classify metabolites based on their anti-aging properties highlighted its potential for identifying drug candidates. By leveraging the patterns and features learned from annotated data in PubChem, our study demonstrated a promising approach to prioritizing metabolites with therapeutic potential. However, we acknowledge the limitations of our work, particularly the inherent weaknesses of current deep-learning methodologies. These limitations include the requirement for large and diverse datasets to ensure robust model training, potential biases in dataset selection, and the interpretability of complex models. Furthermore, while our approach shows promise, additional validation and refinement will be necessary to effectively translate these findings into clinical applications.

## 5 Conclusion

In this study, an integrated method involving a deep learning model and sequential metabolism was used to describe the chemical profiles of HSD both *in vitro* and *in vivo* and to screen potential anti-aging metabolites, providing valuable references for the material basis of HSD research. Additionally, the results of a bioactivity assay also demonstrated that this integrated method could be an effective tool for screening anti-aging metabolites. First, 366 metabolites were identified or tentatively characterized in HSD. Then, based on the results of IPVS and oral drug administration, we identified 135 metabolites absorbed in plasma and the absorption process of HSD. A deep learning model and bioactivity assessment assay were used to screen potential anti-aging metabolites. Ginsenoside Rg1, Rg2, and Rc, pseudoginsenoside F11, and jionoside B1 were selected as potential anti-aging metabolites. This proposed approach could serve as a powerful tool for screening potential anti-aging metabolites of botanical drugs.

## Data Availability

The original contributions presented in the study are included in the article/Supplementary Material, further inquiries can be directed to the corresponding authors.

## Abbreviations

AMPK, AMP-activated protein kinase; AUPRC, area under the receiver operating characteristic curve; AUROC, area under the precision–recall curve; BAN, bilinear attention network; CAT, catalase; DTA, drug–target affinity; FRTA, free radical theory of aging; GCN, graph convolutional network; HSD, Huan Shao Dan; IPVS, *in situ* intestinal perfusion with venous sampling; LDH, lactate dehydrogenase; MDA, malondialdehyde; NO, nitric oxide; OTCML, Orbitrap Traditional Chinese Medicine Library; ROS, reactive oxygen species; SOD, superoxide dismutase; TIC, total ion chromatogram; TCM, traditional Chinese medicine.
