# Supplementary material for: Rapid identification of chemical profiles in vitro and in vivo of Huan Shao Dan and potential anti-aging metabolites by high-resolution mass spectrometry, sequential metabolism, and deep learning model
Source: Front Pharmacol. 2024 Oct 15;15:1432592. doi: 10.3389/fphar.2024.1432592 (PMC11518704; doi:10.3389/fphar.2024.1432592)
Supplement: Supplementary file 1 [file DataSheet1.docx]

Supplementary Material

# Supplementary Data

| **TCM materials** | **Batch number** |
| --- | --- |
| *Polygonum multiflorum* Thunb.  [Polygonaceae; Polygoni multiflori radix] | 000181291 |
| *Achyranthes bidentata* Bl.  [Amaranthaceae; Achyranthis bidentatae radix] | 211216001 |
| *Rehmannia glutinosa* Libosch.  [Scrophulariaceae; Rehmanniae radix] | 210831001 |
| *Cistanche deserticola* Y.C.Ma  [Orobanchaceae; Cistanches herba] | 21010801 |
| *Phellodendron chinense* Schneid.  [Rutaceae; Phellodendri chinensis cortex] | 21052602 |
| *Psoralea corylifolia* L.  [Leguminosae; Psoraleae fructus] | 19101903 |
| *Plantago asiatica* L.  [Plantaginaceae; Plantaginis semen] | 21031802 |
| *Platycladus orientalis* (L.) Franco  [Cupressaceae; Platycladi semen] | 20111301 |
| *Dioscorea opposita* Thunb.  [Dioscoreaceae; Dioscoreae rhizoma] | 21071701 |
| *Angelica sinensis* (Oliv.) Diels  [Apiaceae; Angelicae sinensis radix] | 21082601 |
| *Cuscuta australis* R.Br.  [Convolvulaceae; Cuscutae semen] | 210368 |
| *Panax ginseng* C. A. Mey.  [Araliaceae; Ginseng radix et rhizoma] | 20210912 |
| *Schisandra chinensis* (Turcz.) Baill  [Magnoliaceae; Schisandrae chinensis fructus] | 21051504 |

| **Reference standards** | **Purity** | **Batch number** | **Manufacturer** |
| --- | --- | --- | --- |
| Phthalic acid | ≥98% | Y14J9Y18890 | Shanghai Yuanye Pharmaceutical Technology Co., Ltd. (Shanghai, China) |
| Phenylalanine | ≥98% | J04J7R8481 |  |
| Eriodictyol | ≥98% | Y19S9H70757 |  |
| Naringenin | ≥98% | YJ0603HA13 |  |
| Neochlorogenic acid | ≥98% | Y25S9H66035 |  |
| Genistein | ≥98% | H30A9Z69019 |  |
| Kaempferol | ≥98% | C26J8Y38642 |  |
| Astragalin | ≥98% | Y19M8H36474 |  |
| Magnoflorine | ≥98% | R21M9F61834 |  |
| Rutin | ≥98% | Y22S6S3719 |  |
| Hyperoside | ≥98% | Y04A9X62302 |  |
| Isoquercetin | ≥98% | P28S8F45095 |  |
| Epiberberine | ≥98% | W11A9Z58574 |  |
| Berberine | ≥98% | Y31J9H67024 |  |
| Columbamine | ≥98% | W21J9Z66216 |  |
| Schizandrin A | ≥98% | Z23J7B16583 |  |
| Acteoside | ≥98% | Y03F9H54484 |  |
| Ginsenoside Re | ≥98% | P27N6F6512 |  |
| Schisantherin A | ≥98%, | D22011308 | Nanjing Dilger Medical Technology Co., Ltd. (Nanjing, China) |
| Angeloyl gomisin H | ≥98%, | D23010409 |  |

| **Reference standards** | **Purity** | **Batch number** | **Manufacturer** |
| --- | --- | --- | --- |
| Angelicin | 99.99% | MUST-21062520 | Chengdu Must Bio-Technology Co., Ltd. (Chengdu, China) |
| Cryptochlorogenic acid | 99.07% | MUST-16022403 |  |
| Levistilide A | ≥98% | MUST-14082810 |  |
| Daidzein | ≥98% | MUST-12020708 |  |
| Daidzin | ≥98% | MUST-11121201 |  |
| Calycosin | ≥98% | MUST-14051511 |  |
| 1,5-Dicaffeoylquinic Acid | 99.29% | MUST-16040105 |  |
| Gallic acid | 90.80% | 110831-201605 | National Institutes for Food and Drug Control (Beijing, China) |
| Caffeic acid | 99.70% | 110885-201703 |  |
| Adenosine | 99.70% | 110879-201703 |  |
| Ferulic acid | 99.00% | 1110773-201614 |  |
| Chlorogenic acid | 96.80% | 110753-201817 |  |
| Aloe-emodin | 98.30% | 110795-201710 |  |
| Apigenin | 99.20% | 111901-201603 |  |
| Emodin | 98.70% | 110756-201512 |  |
| Luteolin | 99.60% | 111520-201605 |  |
| Cianidanol | 99.20% | 110877-201604 |  |
| Epicatechin | 99.70% | 110878-201703 |  |
| Quercetin | 99.10% | 100081-201610 |  |
| Coptisine | 95.10% | 112026-201601 |  |
| Quercitrin | 90.60% | 111538-201606 |  |
| Jateorhizine | 89.50% | 10733-201609 |  |
| Betaine | 99.20% | 110894-201604 |  |
| Ginsenoside Rg1 | 93.40% | 110703-201128 |  |
| Ginsenoside Rb1 | 95.90% | 110704-201223 |  |
| Guanosine | 93.60% | 111977-201501 |  |
| Gomisin J | ＞95% | PS1854 | Chengdu PurechemStandard Co., Ltd. (Chengdu, China) |
| Rehmannioside D | ＞95% | PS011176 |  |
| Cosmosiin | ≥98% | - |  |
| Ligustilide | ≥98% | C13408546 |  |
| Plantamajoside | ≥98% | C12223919 |  |
| Catalpol | ≥98% | 811F022 |  |
| Wogonin | ≥98% | 11040501 |  |
| Procyanidin B1 | >95% | PRF7102743 |  |
| Phellodendrine | ＞98% | 210517 |  |
| Geniposidic acid | ≥98% | DSTDJ004001 | Chengdu Desite Biotech Co., Ltd.  (Chengdu, China) |
| β-ecdysterone | ≥98% | DSTDT002702 |  |
| Echinacoside | ≥98% | DSTDS003801 |  |
| Tetrahydroxystilbene Glucoside | ≥98% | 5574 | Shanghai Standard Biotech Co., Ltd. (Shanghai, China) |
| Schisandrin B | ≥98% | 10388 |  |
| Schisandrol B | ≥98% | 9165 |  |
| Schisandrol A | ≥98% | 2329 |  |
| Psoralen | ≥98% | 10376 |  |

| **Reagents** | **Batch number** | **Manufacturer** |
| --- | --- | --- |
| DMEM basic (1X) | 8123130 | (Thermo Fisher Scientific, USA) |
| Acetonitrile | 172421 |  |
| Formic acid | 205178 |  |
| Fetal Bovine Serum | A0823D | Meilunbio Biological Co.  (Dalian, China) |
| D-gal | C14971408 | Shanghai Macklin Biochemical Technology Co., Ltd  (Shanghai, China) |
| SA-β-Gal Staining Kit | 112323231219 | Biyuntian Biological Co. (Shanghai, China) |
| ROS assay kits | 011124240523 |  |
| NO assay kits | 112223240430 |  |
| LDH assay kits | 20240530 | Nanjing Jiancheng  Institute of Biological Engineering (Nanjing, China) |
| SOD assay kits | 20240330 |  |
| MDA assay kits | 20221118 |  |
| CAT assay kits | 20220920 |  |
| 0.9% Normal saline | 2401181901 | Shijiazhuang No.4 Pharmaceutical Co., Ltd.  (Shijiazhuang, China) |
| CCK-8 | 20231213 | New Cell molecular biotech Co., Ltd. (Suzhou, China) |

**Manufacturer’s protocol of LDH**

| Reagents | Blank | Standard | Sample | Sample-blank |
| --- | --- | --- | --- | --- |
| Deionized water (μL) | 20 | 4 | - | 4 |
| 0.2 μM/mL sodium pyruvate (μL) | - | 16 | - | - |
| Sample (μL) | - | - | 16 | 16 |
| Reagent 1 (μL) | 20 | 20 | 20 | 20 |
| Reagent 2 (μL) | - | - | 4 | - |
| Incubation at 37 ℃ for 15 minutes | | | | |
| Reagent 3 (μL) | 20 | 20 | 20 | 20 |
| Incubation at 37 ℃ for 10 minutes | | | | |
| Reagent 4 (μL) | 200 | 200 | 200 | 200 |
| Place at room temperature for 5 minutes. The absorbance value is measured at wavelength of 440 nm | | | | |

**Manufacturer’s protocol of MDA**

| Reagents | Blank | Standard | Sample | Sample-blank |
| --- | --- | --- | --- | --- |
| Absolute ethyl alcohol (mL) | 0.1 | - | - | - |
| 10 nM/mL Tetrathoxypropane (mL) | - | 0.1 | - | - |
| Sample (mL) | - | - | 0.1 | 0.1 |
| Reagent 1 (mL) | 0.1 | 0.1 | 0.1 | 0.1 |
| Reagent 2 (mL) | 3 | 3 | 3 | 3 |
| Reagent 3 (mL) | 1 | 1 | 1 | 1 |
| 50% Acetic Acid (mL) | - | - | - | 1 |
| Boil at 95 ℃ in a water bath for 40 minutes. After centrifuging at 4000 rpm for 10 minutes, the absorbance value of supernatant is measured at wavelength of 532 nm. | | | | |

**Manufacturer’s protocol of SOD**

| Reagents | Blank | Standard | Sample | Sample-blank |
| --- | --- | --- | --- | --- |
| Sample (μL) | - | - | 20 | 20 |
| Deionized water (μL) | 20 | 20 | - | - |
| Reagent 1 (μL) | 20 | - | 20 | - |
| Reagent 2 (μL) | - | 20 | - | 20 |
| Reagent 3 (μL) | 200 | 200 | 200 | 200 |
| Incubation at 37 ℃ for 20 minutes. The absorbance value is measured at wavelength of 450 nm | | | | |

**Manufacturer’s protocol of CAT**

| Reagents | Sample | Sample-blank |
| --- | --- | --- |
| Sample (mL) | 0.1 | - |
| Reagent 1 (mL) | 1.0 | 1.0 |
| Reagent 2 (mL) | 0.1 | 0.1 |
| Incubation at 37 ℃ for 1 minute | | |
| Reagent 3 (mL) | 1.0 | 1.0 |
| Reagent 4 (μL) | 0.1 | 0.1 |
| Sample (mL) | - | 0.1 |
| The absorbance value is measured at wavelength of 405 nm | | |

**Manufacturer’s protocol of NO**

| Reagents | Sample | Sample-blank |
| --- | --- | --- |
| Sample (μL) | 50 | - |
| Matrix (μL) | - | 50 |
| Griess Reagent I (μL) | 50 | 50 |
| Griess Reagent II (μL) | 50 | 50 |
| The absorbance value is measured at wavelength of 540 nm | | |

# Supplementary Figures and Tables

## Supplementary Figures

**

**

**Supplementary Figure 1.** MS^2^ mass spectra and possible fragment pathways of Emodin from HSD

**

**

**Supplementary Figure 2.** MS^2^ mass spectra and possible fragment pathways of Cosmosiin from HSD

**

**

**Supplementary Figure 3.** MS^2^ mass spectra and possible fragment pathways of Naringenin from HSD

**

**

**Supplementary Figure 4.** MS^2^ mass spectra and possible fragment pathways of Phellodendrine from HSD

**

**

**Supplementary Figure 5.** MS^2^ mass spectra and possible fragment pathways of Berberine from HSD

**

**

**Supplementary Figure 6.** MS^2^ mass spectra and possible fragment pathways of Magnoflorine from HSD

**

**

**Supplementary Figure 7.** MS^2^ mass spectra and possible fragment pathways of Geniposidic acid from HSD

**

**

**Supplementary Figure 8.** MS^2^ mass spectra and possible fragment pathways of Ginsenoside Re from HSD

**

**

**Supplementary Figure 9.** MS^2^ mass spectra and possible fragment pathways of Schizandrin A from HSD




**Supplementary Figure 10.** MS^2^ mass spectra and possible fragment pathways of Schisandrol A from HSD

**

**

**Supplementary Figure 11.** MS^2^ mass spectra and possible fragment pathways of Psoralen from HSD

**

**

**Supplementary Figure 12.** MS^2^ mass spectra and possible fragment pathways of Chlorogenic acid from HSD

**

**

**Supplementary Figure 13.** MS^2^ mass spectra and possible fragment pathways of Echinacoside from HSD

**

**

**Supplementary Figure 14.** MS^2^ mass spectra and possible fragment pathways of Phenylalanine from HSD


**Supplementary Figure 15.** MS^2^ mass spectra and possible fragment pathways of Levistilide A from HSD


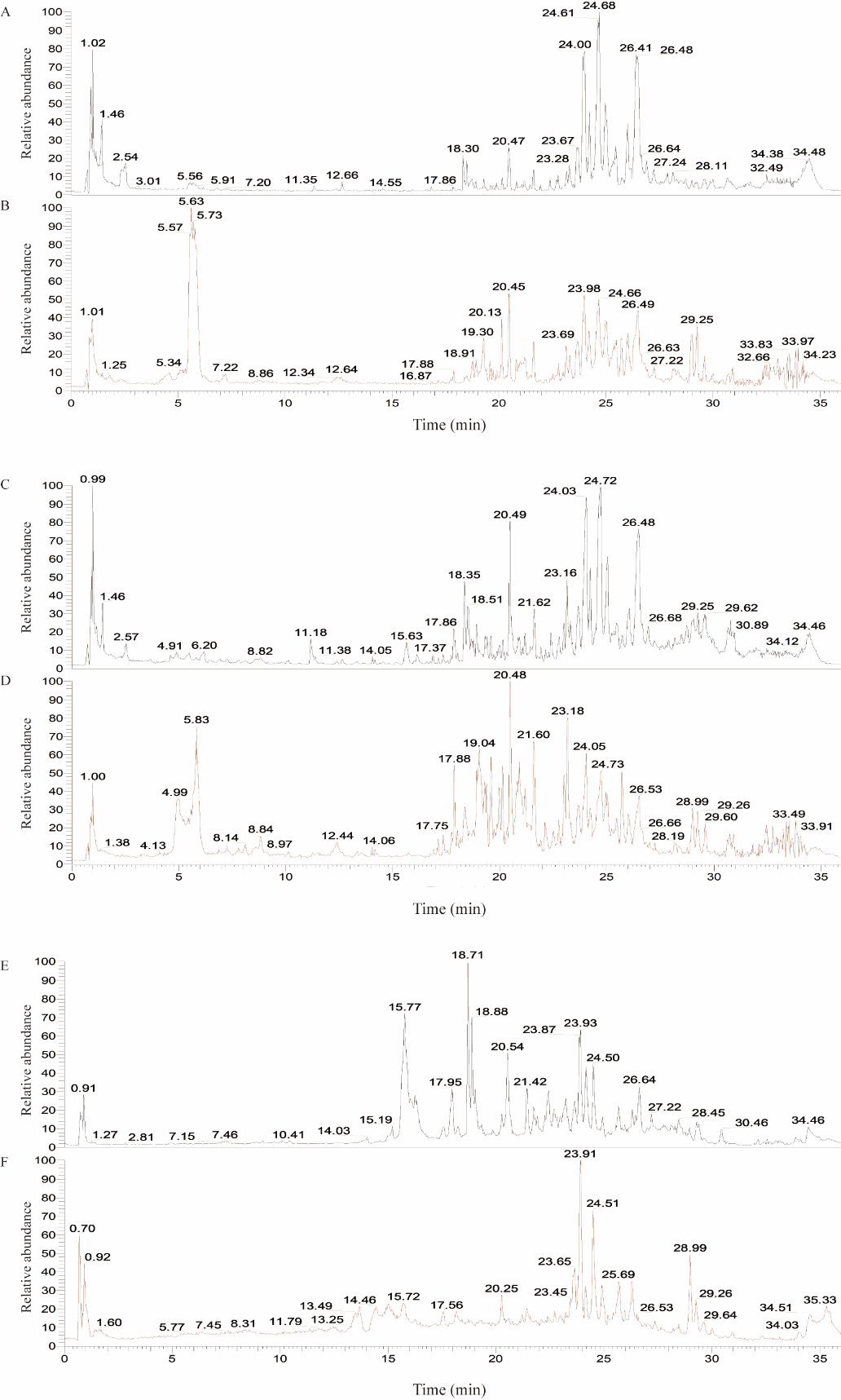


**Supplementary Figure 16.** Total ion chromatogram (TIC) of biological samples from UPLC-Q Exactive-Orbitrap HRMS. Intestinal sample in positive ion mode (A) and negative ion (B). Liver sample Intestinal sample in positive ion mode (C) and negative ion (D). Systemic sample in positive ion mode (E) and negative ion (F).


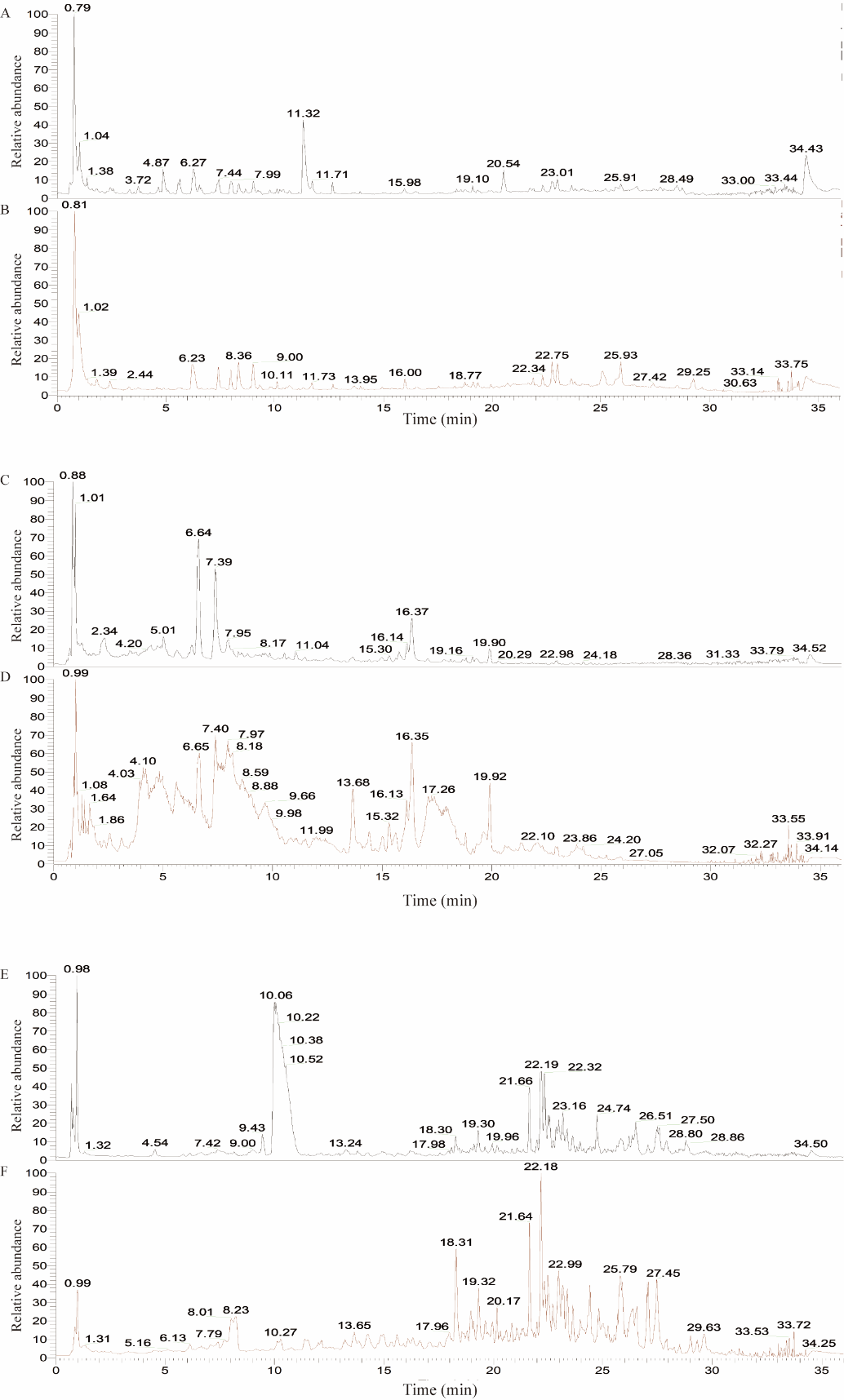


**Supplementary Figure 17.** Total ion chromatogram (TIC) of biological samples from UPLC-Q Exactive-Orbitrap HRMS. Gastric sample in positive ion mode (A) and negative ion (B). Urine sample Intestinal sample in positive ion mode (C) and negative ion (D). Feces sample in positive ion mode (E) and negative ion (F).

**
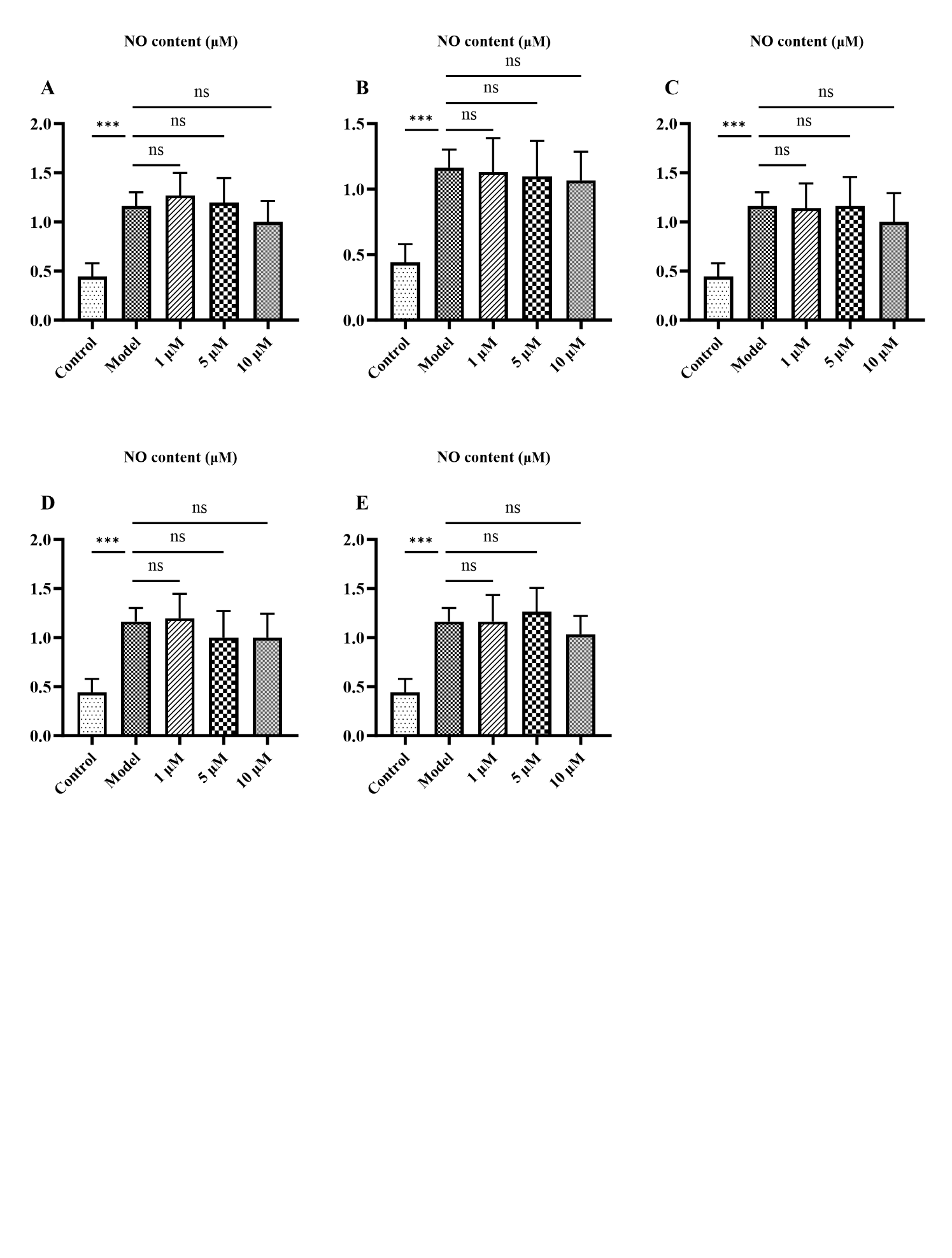
**

**Supplementary Figure 18.** Effects of candidate compounds on NO level in PC12 cells. (A) Ginsenoside Rg1. (B) Ginsenoside Rg2. (C) Ginsenoside Rc. (D) Pseudoginsenoside F11. (E) Jionoside B1. Data were presented as mean ± SD from each group. *p < 0.05, **p < 0.01 and ***p < 0.001 were contrasted to control and model group.

## Supplementary Tables

**Supplementary Table S1. Identification of chemical compounds of HSD by UPLC-Q Exactive-Orbitrap HRMS in positive ion mode**

| Peak No. | tR  (min) | Proposed compounds | Predicted mass  (Da) | Molecular formula | Error  (ppm) | Observed mass  (Da) | Major fragments |
| --- | --- | --- | --- | --- | --- | --- | --- |
| 1 | 0.63 | Arginine | 175.1190 | C_6_H_14_N_4_O_2_ | 0.273 | 175.1190 | 158.0294[M+H-OH]^+^, 130.0975[M+H-COOH]^+^, 116.0708[M+H-CH_5_N_3_]^+^, 70.0658[M+H-C_2_H_7_N_3_O_2_]^+^ |
| 3^*^ | 0.79 | Betaine | 118.0863 | C_5_H_11_NO_2_ | 2.581 | 118.0866 | 59.0738[M+H-C_2_H_3_O_2_]^+^, 58.0660[M+H-C_2_H_4_O_2_]^+^ |
| 9 | 0.85 | Adenine | 136.0618 | C_5_H_5_N_5_ | -0.234 | 136.0617 | 119.0355[M+H-NH_3_]^+^ |
| 11^*^ | 0.88 | Guanosine | 284.0989 | C_10_H_13_N_5_O_5_ | -0.898 | 284.0987 | 284.0987[M+H]^+^ |
| 12 | 0.91 | Achyranthine | 130.0863 | C_6_H_11_NO_2_ | 0.729 | 130.0864 | 84.0814[M+H-COOH]^+^ |
| 13 | 0.91 | Phenylacetaldehyde | 121.0648 | C_8_H_8_O | 1.888 | 121.0650 | 103.0547[M+H-H_2_O]^+^, 93.0705[M+H-CO]^+^ |
| 16 | 0.94 | Proline | 116.0706 | C_5_H_9_NO_2_ | 2.799 | 116.0709 | 98.0605[M+H-H_2_O]^+^, 70.0659[M+H-COOH]^+^ |
| 17 | 0.97 | Nicotinic acid | 124.0393 | C_6_H_5_NO_2_ | 1.492 | 124.0395 | 80.0502[M+H-CO_2_]^+^ |
| 18 | 1.1 | Candicine | 180.1383 | C_11_H_18_NO | 0.273 | 180.1383 | 121.0651[M-N(CH_3_)_3_]^+^, 93.0704[M-N(CH_3_)_3_-OH]^+^, 77.0393[M-N(CH_3_)_3_-OH-CH_4_]^+^ |
| 19^*^ | 1.13 | Adenosine | 268.1040 | C_10_H_13_N_5_O_4_ | -0.337 | 268.1039 | 136.0619[M+H-C_5_H_8_O_4_]^+^, 119.0356[M+H-C_5_H_8_O_4_-NH_3_]^+^ |
| 20 | 1.13 | L-Pyroglutamic acid | 130.0499 | C_5_H_7_NO_3_ | 1.233 | 130.0500 | 84.0450[M+H-COOH]^+^ |
| 21 | 1.13 | Leucine | 132.1019 | C_6_H_13_NO_2_ | 0.793 | 132.1020 | 86.0970[M+H-COOH]^+^, 69.0707[M+H-COOH-NH_3_]^+^ |
| 22 | 1.13 | Tyrosine | 182.0812 | C_9_H_11_NO_3_ | 0.221 | 182.0812 | 165.0547[M+H-NH_3_]^+^, 136.0758[M+H-COOH]^+^ |
| 24 | 1.19 | Guanine | 152.0567 | C_5_H_5_N_5_O | 0.616 | 152.0568 | 135.0302[M+H-NH_3_]^+^, 110.0354[M+H-CH_2_N_2_] |
| 25^*^ | 1.25 | Catalpol | 363.1286 | C_15_H_22_O_10_ | -6.150 | 363.1263 | 363.1263[M+H]^+^ |
| 27^*^ | 1.38 | Phenylalanine | 166.0863 | C_9_H_11_NO_2_ | 0.752 | 166.0864 | 149.0594[M+H-NH_3_]^+^, 120.0810[M+H-COOH]^+^, 103.0546[M+H-NH_3_-COOH]^+^ |
| 31 | 1.85 | Maltol or isomer | 127.0390 | C_6_H_6_O_3_ | 1.412 | 127.0392 | 109.0288[M+H-H_2_O]^+^, 81.0341[M+H-H_2_O-CO]^+^, 53.0394[M+H-H_2_O-2CO]^+^ |
| 32 | 2.05 | 5-hydroxymethylfurfural | 127.0390 | C_6_H_6_O_3_ | 1.191 | 127.0392 | 109.0288[M+H-H_2_O]^+^, 97.0291[M+H-CHO]^+^, 81.0341[M+H-H2O-CO]^+^ |
| 33 | 2.33 | Tryptophan | 205.0972 | C_11_H_12_N_2_O_2_ | 0.808 | 205.0973 | 188.0708[M+H-NH_3_]^+^, 159.0918[M+H-COOH]^+^ |
| 35 | 2.52 | Higenamine | 272.1281 | C_16_H_17_NO_3_ | -0.147 | 272.1281 | 255.1017[M+H-NH_3_]^+^, 237.0912[M+H-NH_3_-H_2_O]^+^, 209.0963[M+H-NH_3_-H_2_O-CO]^+^ |
| 36 | 2.55 | N-caffeoyltryptophan | 205.0972 | C_20_H_18_N_2_O_5_ | 0.418 | 205.0972 | 188.0707[M+H-NH_3_]^+^ |
| 44 | 3.27 | Lotusine | 314.1751 | C_19_H_24_NO_3_ | -0.160 | 314.1750 | 269.1171[M-(CH_3_)_2_NH]^+^, 237.0915[M-(CH_3_)_2_NH-CH_3_OH]^+^, 107.0496[M-C_12_H_16_NO_2_]^+^ |
| 48 | 3.47 | Magnocurarine | 314.1751 | C_19_H_24_NO_3_ | -0.064 | 314.1751 | 269.1173[M-(CH_3_)_2_NH]^+^, 237.0909[M-(CH_3_)_2_NH-CH_3_OH]^+^, 107.0496[M-C_12_H_16_NO_2_]^+^ |
| 50 | 3.71 | 3,4-Dihydro-1-[(4-hydroxyphenyl)methyl]-7-methoxy-2-methyl-6-isoquinolinol. | 298.1443 | C_18_H_20_NO_3_ | 0.000 | 298.1438 | 283.1204[M-CH_3_]^+^, 255.1256[M-CH_3_-CO]^+^ |
| 52 | 4.12 | 7-hydroxycoumarin | 163.0390 | C_9_H_6_O_3_ | 0.180 | 163.0390 | 145.0281[M+H-H_2_O]^+^, 135.0442[M+H-CO]^+^, 117.0338[M+H-CO-H_2_O]^+^ |
| 58^*^ | 4.52 | Phellodendrine | 342.1700 | C_20_H_24_NO_4_ | -0.277 | 342.1699 | 192.1021[M-C_9_H_10_O_2_]^+^, 177.0785[M-C_9_H_10_O_2_-CH_3_]^+^ |
| 59 | 4.55 | Phellodendrine derivative | 356.1498 | C_20_H_22_NO_5_ | -0.559 | 356.1491 | 206.0815[M-C_9_H_10_O_2_]^+^, 191.0583[M-C_9_H_10_O_2_-CH_3_]^+^ |
| 60 | 4.59 | Abscisic acid | 265.1434 | C_15_H_20_O_4_ | -0.036 | 265.1434 | 247.1329[M+H-H_2_O]^+^ |
| 62 | 4.72 | Sanjoinine K | 286.1438 | C_17_H_19_NO_3_ | 0.000 | 286.1438 | 269.1173[M+H-NH_3_]^+^, 254.0931[M+H-NH_3_-CH_3_]^+^, 237.0910[M+H-NH_3_-CH_3_-OH]^+^ |
| 64^*^ | 5.12 | Magnoflorine | 342.1705 | C_20_H_24_NO_4_ | 0.162 | 342.1700 | 297.1123[M-(CH_3_)_2_NH]^+^, 282.0882[M-(CH_3_)_2_NH-CH_3_]^+^, 265.0859[M-(CH_3_)_2_NH-CH_3_OH]^+^, 237.0906[M-(CH_3_)_2_NH-CH_3_OH-CO]^+^ |
| 65 | 5.26 | N-methylhigenamine7-glucopyranoside | 448.1966 | C_23_H_29_NO_8_ | 0.216 | 448.1967 | 286.1438[M+H-Glc]^+^, 255.1017[M+H-Glc-CH_3_NH_2_]^+^, 107.0496[C_7_H_7_O]^+^ |
| 70 | 5.6 | Laudanosine | 358.2013 | C_21_H_27_NO_4_ | 0.014 | 358.2013 | 343.1778[M+H-CH_3_]^+^, 327.1829[M+H-OCH_3_]^+^, 313.1436[M+H-CH_3_-OCH_2_]^+^ |
| 71 | 5.71 | Plantagoguanidinic acid A | 226.1550 | C_11_H_19_N_3_O_2_ | 0.869 | 226.1552 | 208.1446[M+H-H_2_O]^+^, 84.0563[M+H-C_8_H_14_O_2_]^+^ |
| 73 | 5.74 | Plantagoguanidinic acid A or isomer | 226.1550 | C_11_H_19_N_3_O_2_ | 0.781 | 226.1552 | 208.1446[M+H-H_2_O]^+^, 84.0563[M+H-C_8_H_14_O_2_]^+^ |
| 74 | 5.77 | Cassythidine | 312.1230 | C_18_H_17_NO_4_ | 0.242 | 312.1231 | 297.0996[M+H-CH_3_]^+^, 240.1014[M+H-C_3_H_4_O_2_]^+^, 177.0786[M+H-C_8_H_7_O_2_]^+^ |
| 75 | 5.81 | Oblongine | 314.1751 | C_19_H_24_NO_3_ | -0.064 | 314.1751 | 299.1507[M-CH_3_]^+^, 269.1174[M-(CH_3_)_2_NH]^+^, 237.0911[M-(CH_3_)_2_NH-CH_3_OH]^+^, 107.0496[M-C_12_H_16_NO_2_]^+^ |
| 78 | 6.22 | Dauricine | 356.1862 | C_21_H_26_NO_4_ | 0.352 | 356.1858 | 311.1279[M-(CH_3_)_2_NH]^+^, 296.1042[M-(CH_3_)_2_NH-CH_3_]^+^, 279.1016[M-(CH_3_)_2_NH-CH_3_OH]^+^, 264.0781[M-(CH_3_)_2_NH-CH_3_-CH_3_OH]^+^ |
| 80 | 6.25 | N‑methyltetrahydrocolumbamine | 356.1856 | C_21_H_26_NO_4_ | 0.352 | 356.1858 | 192.1021[M−(CH_3_)_2_NH−CH_3_OH−CO−C_2_H_4_]^+^, 177.0788[M−(CH_3_)_2_NH−CH_3_OH−CO−C_2_H_4_-CH_3_]^+^ |
| 86 | 6.58 | Dasycarpamin | 304.1543 | C_17_H_21_NO_4_ | -0.015 | 304.1543 | 286.1440[M+H-H_2_O]^+^, 271.1204[M+H-H_2_O-CH_3_]^+^, 232.0970[M+H-H_2_O-C_4_H_6_]^+^, 202.0864[M+H-C_4_H_8_O-CH_2_O]^+^ |
| 87 | 6.62 | Phellodendrine isomer | 342.1705 | C_20_H_24_NO_4_ | -0.014 | 342.1700 | 192.1021[M-C_9_H_10_O_2_]^+^, 177.0786[M-C_9_H_10_O_2_-CH_3_]^+^ |
| 90 | 6.75 | Tembetarine | 344.1862 | C_20_H_26_NO_4_ | 0.189 | 344.1857 | 299.1279[M-(CH_3_)_2_NH]^+^, 206.1188[M-C_8_H_10_O_2_]^+^, 175.0754[M-C_9_H_15_NO_2_]^+^, 137.0598[M-C_12_H_17_NO_2_]^+^ |
| 95 | 7.06 | Senkyunolide J or isomer | 227.1278 | C_12_H_18_O_4_ | 0.372 | 227.1279 | 209.1175[M+H-H_2_O]^+^, 191.1068[M+H-2H_2_O]^+^ |
| 102 | 7.53 | Tetrahydropalmatine | 356.1856 | C_21_H_25_NO_4_ | 0.267 | 356.1857 | 341.1617[M+H-CH_3_]^+^,192.1021[M+H-C_10_H_12_O_2_]^+^, 165.0914[M+H-C_11_H_13_O_2_N]^+^ |
| 104 | 7.56 | Yuanhunine or isomer | 356.1856 | C_21_H_25_NO_4_ | 0.436 | 356.1858 | 341.1617[M+H-CH_3_]^+^, 192.1021[M+H-C_9_H_10_O_2_]^+^, 177.0785[M+H-C_9_H_10_O_2_-CH_3_]^+^, 165.0914[M+H-C_11_H_13_NO_2_]^+^ |
| 106 | 7.91 | Hydroxyberberine | 352.1179 | C_20_H_18_NO_5_ | -0.338 | 352.1178 | 337.0944[M-CH_3_]^+^, 336.0866[M-CH_4_]^+^,334.1072[M-H_2_O]^+^, 322.0710[M-2CH_3_]^+^, 308.0917[M-CH_4_-CO]^+^ |
| 108 | 8.11 | Berberastine isomer | 352.1179 | C_20_H_18_NO_5_ | -0.068 | 352.1179 | 337.0947[M-CH_3_]^+^, 336.0867[M-CH_4_]^+^, 322.0709[M-2CH_3_]^+^, 308.0919[M-CH_4_-CO]^+^, 294.0762[M-2CH_3_-CO]^+^ |
| 109 | 8.25 | Demethyleneberberine | 324.1236 | C_19_H_18_NO_4_ | 0.325 | 324.1231 | 309.0997[M-CH_3_]^+^,308.0918[M-CH_4_]^+^, 294.0763[M-2CH_3_]^+^, 280.0969[M-CH_4_-CO]^+^, 266.0812[M-2CH_3_-CO]^+^ |
| 112 | 8.35 | Noroxyhydrastinine | 192.0655 | C_10_H_9_NO_3_ | 0.470 | 192.0656 | 174.0550[M+H-H_2_O]^+^, 164.0339[M+H-C_2_H_4_]^+^ |
| 114 | 8.53 | Demethyleneberberine isomer | 324.1236 | C_19_H_18_NO_4_ | 0.325 | 324.1231 | 309.0997[M-CH_3_]^+^,308.0914[M-CH_4_]^+^,294.0762[M-2CH_3_]^+^, 280.0973[M-CH_4_-CO]^+^, 266.0815[M-2CH_3_-CO]^+^ |
| 117 | 8.75 | Apioside or isomer | 565.1552 | C_26_H_28_O_14_ | -0.056 | 565.1552 | 547.1448[M+H-H_2_O]^+^ |
| 120 | 8.89 | Cinnamic acid | 149.0597 | C_9_H_8_O_2_ | 1.167 | 149.0599 | 149.0599[M+H]^+^ |
| 124 | 9.05 | Isoplatydesmine or isomer | 260.1281 | C_15_H_17_NO_3_ | 0.077 | 260.1281 | 242.1177[M+H-H_2_O]^+^, 227.0940[M+H-H_2_O-CH_3_]^+^,188.0707[M+H-C_4_H_8_O]^+^ |
| 125^*^ | 9.05 | Daidzin | 417.1180 | C_21_H_20_O_9_ | -0.141 | 417.1180 | 255.0654[M+H-Glc]^+^, 227.0703[M+H-Glc-CO], 137.0234[M+H-Glc-C_8_H_6_O]^+^ |
| 128^*^ | 9.49 | Columbamine | 338.1387 | C_20_H_20_NO_4_ | -0.398 | 338.1386 | 323.1153[M-CH_3_]^+^, 322.1075[M-CH_4_]^+^, 308.0919[M-2CH_3_]^+^, 294.1123[M-CH_4_-CO]^+^, 280.0968[M-2CH_3_-CO]^+^ |
| 129 | 9.52 | N-methyl canadine | 354.1705 | C_21_H_24_NO_4_ | -0.183 | 354.1699 | 190.0864[M-C_10_H_12_O_2_]^+^ |
| 131 | 9.69 | Thalifendine or isomer | 322.1074 | C_19_H_15_NO_4_ | 0.110 | 322.1074 | 307.0840[M+H-CH_3_]^+^, 279.0889[M+H-CH_3_-CO]^+^ |
| 132^*^ | 9.76 | Jateorhizine | 338.1392 | C_20_H_20_NO_4_ | -0.309 | 338.1386 | 323.1152[M-CH_3_]^+^, 322.1075[M-CH_4_]^+^, 308.0919[M-2CH_3_]^+^, 294.1123[M-CH_4_-CO]^+^, 280.0968[M-2CH_3_-CO]^+^ |
| 137 | 9.92 | Anisic acid | 153.0546 | C_8_H_8_O_3_ | 1.237 | 153.0548 | 153.0548[M+H]^+^ |
| 140 | 10.13 | Stachysterone C | 463.3054 | C_27_H_42_O_6_ | 0.074 | 463.3055 | 301.1799[M+H-C_8_H_18_O_3_]^+^ |
| 141^*^ | 10.16 | β-ecdysterone | 481.3160 | C_27_H_44_O_7_ | 0.498 | 481.3162 | 463.3061[M+H-H_2_O]^+^, 371.2219[M+H-C_4_H_14_O_3_]^+^, 319.1910[M+H-C_8_H_18_O_3_]^+^, 301.1805[M+H-C_8_H_18_O_3_-H_2_O]^+^ |
| 143 | 10.26 | 4,7-Dihydroxy-3-butylphthalide or isomer | 223.0965 | C_12_H_14_O_4_ | 0.738 | 223.0967 | 223.0967[M+H]^+^ |
| 146 | 10.3 | Berberrubine | 322.1074 | C_19_H_15_NO_4_ | 0.297 | 322.1075 | 307.0841[M+H-CH_3_]^+^, 292.0593[M+H-2CH_3_]^+^, 279.0891[M+H-CH_3_-CO]^+^, 264.0665[M+H-2CH_3_-CO]^+^, 251.0947[M+H-CH_3_-2CO]^+^ |
| 148 | 10.51 | Dehydrocorybulbine | 352.1549 | C_21_H_22_NO_4_ | -0.013 | 352.1543 | 336.1230[M-CH_4_]^+^, 322.1070[M-2CH_3_]^+^,308.1260[M-CH_4_-CO]^+^, 294.1124[M-2CH_3_-CO]^+^ |
| 150^*^ | 10.54 | Epiberberine | 336.1230 | C_20_H_18_NO_4_ | -0.222 | 336.1230 | 320.0919[M-CH_4_]^+^, 306.0760[M-2CH_3_]^+^, 292.0968[M-CH_4_-CO]^+^, 278.0809[M-2CH_3_-CO]^+^ |
| 151^*^ | 10.57 | Coptisine | 320.0917 | C_19_H_14_NO_4_ | -5.918 | 320.0898 | 292.0965[M-CO]^+^, 277.0773[M-CO-CH_3_]^+^, 262.0859[M-2CO-2H]^+^ |
| 154 | 10.88 | Palmatine | 352.1549 | C_21_H_22_NO_4_ | -0.638 | 352.1541 | 337.1308[M-CH_3_]^+^,336.1229[M-CH_4_]^+^, 322.1073[M-2CH_3_]^+^,308.1281[M-CH_4_-CO]^+^, 294.1124[M-2CH_3_-CO]^+^ |
| 155^*^ | 10.91 | Berberine | 336.1236 | C_20_H_18_NO_4_ | -0.519 | 336.1229 | 321.09958[M-CH_3_]^+^, 320.0919[M-CH_4_]^+^, 306.0763[M-2CH_3_]^+^, 292.0969[M-CH_4_-CO]^+^, 278.0814[M-2CH_3_-CO]^+^ |
| 156 | 10.91 | Isoacteoside isomer | 625.2127 | C_29_H_36_O_15_ | 0.901 | 625.2133 | 163.0391[M+H-Rha-Glc-C_8_H_10_O_3_]^+^ |
| 159 | 11.01 | (*Z*)-4',6-dihydroxyaurone_6_-glucoside or isomer | 417.1180 | C_21_H_20_O_9_ | -0.788 | 417.1177 | 255.0664[M+H-Glc]^+^, 213.0559[M+H-Glc-C_2_H_2_O]^+^, |
| 161 | 11.06 | Senkyunolide F or isomer | 207.1016 | C_12_H_14_O_3_ | 3.569 | 207.1023 | 179.1069[M+H-CO]^+^, 161.0962[M+H-CO-H_2_O]^+^, 123.0444[M+H-CO-C_4_H_8_]^+^ |
| 165 | 11.25 | Phellamurin | 519.1861 | C_26_H_30_O_11_ | 0.370 | 519.1863 | 357.1332[M+H-Glc]^+^, 339.1229[M+H-Glc-H_2_O]^+^ |
| 181^*^ | 12.2 | Isoquercetin | 465.1028 | C_21_H_20_O_12_ | 0.597 | 465.1030 | 303.0500[M+H-Glc]^+^, 274.0478[M+H-Glc-CHO]^+^, 153.0183[M+H-C_8_H_6_O_3_]^+^ |
| 191 | 12.72 | Oxyberberine | 352.1179 | C_20_H_17_NO_5_ | -0.338 | 352.1178 | 337.0947[M+H-CH_3_]^+^, 322.0715[M+H-2CH_3_]+, 308.0919[M+H-C_2_H_4_O]^+^, 294.0760[M+H-C_3_H_6_O]^+^ |
| 196 | 13.03 | Kaempferol-3-O-β-D-glucoside | 449.1078 | C_21_H_20_O_11_ | 0.294 | 449.1080 | 287.0551[M+H-Glc]^+^, 258.0512[M+H-Glc-CHO]^+^, 153.0186[C_7_H_5_O_4_]^+^, 121.0286[C_7_H_5_O_2_]^+^ |
| 203^*^ | 13.38 | Astragalin | 449.1078 | C_21_H_20_O_11_ | -0.107 | 449.1078 | 287.0551[M+H-Glc]^+^, 258.0524[M+H-Glc-CHO]^+^, 153.0185[M+H-Glc-C_8_H_6_O_2_]^+^, |
| 208 | 13.51 | Isorhamnetin isomer | 317.0656 | C_16_H_12_O_7_ | -0.092 | 317.0656 | 302.0421[M+H-CH_3_]^+^, 273.0393[M+H-CH_3_-CHO]^+^, 257.0443[M+H-CH_3_-CHO_2_]^+^ |
| 229 | 14.9 | Tomentosanol D or isomer | 357.1333 | C_20_H_20_O_6_ | 0.014 | 357.1333 | 165.0185[M+H-C_12_H_16_O_2_]^+^, 137.0233[M+H-C_12_H_16_O_2_-CO]^+^ |
| 230 | 15 | N-feruloyl-3-methoxytyramine | 344.1492 | C_19_H_21_NO_5_ | 0.031 | 344.1493 | 177.0548[M+H-C_9_H_13_NO_2_]^+^, 145.0285[M+H-C_9_H_13_NO_2_-CH_3_-OH]^+^, 117.0338[M+H-C_9_H_13_NO_2_-CH_3_-OH-CO]^+^ |
| 232 | 15.1 | Butylidene phthalide or isomer | 189.0910 | C_12_H_12_O_2_ | 0.126 | 189.0910 | 171.0805[M+H-H_2_O]^+^,153.0697[M+H-2H_2_O]^+^, 143.0857[M+H-H_2_O-CO]^+^ |
| 234 | 15.28 | Astrapterocarpan | 301.1071 | C_17_H_16_O_5_ | 0.132 | 301.1071 | 283.0968[M+H-H_2_O]^+^, 270.0890[M+H-OCH_3_]^+^ |
| 235 | 15.31 | Senkyunolide | 189.0910 | C_12_H_12_O_2_ | 0.390 | 189.0911 | 171.0806[M+H-H_2_O]^+^,161.0598[M+H-C_2_H_4_]^+^, 147.0441[M+H-C_3_H_6_]^+^ |
| 240^*^ | 15.76 | Psoralen | 187.0390 | C_11_H_6_O_3_ | 0.638 | 187.0391 | 159.0441[M+H-CO]^+^, ,143.0493[M+H-CO_2_]^+^, 131.0494[M+H-2CO]^+^, 115.0546[M+H-CO-CO_2_]^+^ |
| 244 | 16.14 | Hinokinin | 355.1176 | C_20_H_18_O_6_ | -0.182 | 355.1176 | 337.1070[M+H-H_2_O]^+^, 319.0967[M+H-2H_2_O]^+^, 135.0440[M+H-C_12_H_12_O_4_]^+^ |
| 246^*^ | 16.27 | Angelicin | 187.0390 | C_11_H_6_O_3_ | 0.318 | 187.0391 | 159.0443[M+H-CO]^+^, 143.0493[M+H-CO_2_]^+^, 131.0494[M+H-2CO]^+^, 115.0546[M+H-CO-CO_2_]^+^ |
| 247^*^ | 16.47 | Daidzein | 255.0652 | C_15_H_10_O_4_ | -0.099 | 255.0652 | 237.0549[M+H-H_2_O] ^+^, 227.0703[M+H-CO] ^+^, 199.0756 [M+H-2CO] ^+^, 137.0233[M+H-C_8_H_6_O]^+^ |
| 248^*^ | 16.51 | Calycosin | 285.0758 | C_16_H_12_O_5_ | -0.982 | 285.0755 | 270.0523[M+H-CH_3_] ^+^, 253.0496[M+H-CH_3_-OH] ^+^, 225.0548[M+H-CH_3_-OH-CO] ^+^, 197.0597[M+H-CH_3_-OH-CO-CO] ^+^ |
| 251 | 16.92 | Pepper alkaloid | 230.0812 | C_13_H_11_NO_3_ | 0.479 | 230.0813 | 215.0578[M+H-CH_3_]^+^, 200.0343[M+H-2CH_3_]^+^,186.0550[M+H-C_2_H_4_O]^+^, 172.0392[M+H-2CH_3_-CO]^+^ |
| 254 | 17.37 | Oleanolic acid or isomer | 457.3676 | C_30_H_48_O_3_ | -0.223 | 457.3675 | 421.3467[M+H-2H_2_O]^+^, |
| 263 | 17.99 | Palmitic acid | 257.2475 | C_16_H_32_O_2_ | 2.345 | 257.2481 | 257.2481[M+H]^+^ |
| 264 | 18.06 | Dehydrocorybulbine or isomer | 352.1543 | C_21_H_22_NO_4_ | -0.269 | 352.1542 | 336.1224[M-CH_3_]^+^, 308.0911[M-2CH_3_]^+^ |
| 265 | 18.1 | Bavachromanol | 341.1384 | C_20_H_20_O_5_ | -0.323 | 341.1382 | 323.1278[M+H-H_2_O]^+^, 221.0810[M+H-C_8_H_8_O]^+^, 203.0704[M+H-C_8_H_8_O-H_2_O]^+^, 149.0235[M+H-C_8_H_8_O-H_2_O-C_4_H_6_]^+^ |
| 266 | 18.2 | Butylphthalide | 191.1067 | C_12_H_14_O_2_ | -0.242 | 191.1066 | 173.0964[M+H-H_2_O]^+^, 145.1014[M+H-H_2_O-CO]^+^ |
| 278 | 18.71 | Euchrenone a_7_ or isomer | 341.1384 | C_20_H_20_O_5_ | -0.323 | 341.1382 | 323.1274[M+H-H_2_O]^+^, 305.1170[M+H-2H_2_O]^+^, 269.0806[M+H-H_2_O-C_4_H_6_]^+^, 161.0231[M+H-C_11_H_16_O_2_]^+^ |
| 279 | 18.81 | 10,12-Octadecanedioic acid | 277.2162 | C_18_H_28_O_2_ | 0.048 | 277.2162 | 259.2058[M+H-H_2_O]^+^, 241.1941[M+H-2H_2_O]^+^ |
| 288 | 19.27 | Corylidin or isomer | 369.0969 | C_20_H_16_O_7_ | -0.296 | 369.0968 | 297.0394[M+H-C_4_H_8_O]^+^, 269.0455[M+H-C_4_H_8_O-CO]^+^ |
| 293 | 19.68 | Limonin | 471.2013 | C_26_H_30_O_8_ | 0.479 | 471.2016 | 425.1960[M+H-CH_2_O_2_]^+^, 367.1898[M+H-C_3_H_4_O_5_]^+^, 213.0912[M+H-C_12_H_18_O_6_]^+^, 161.0599[C_10_H_9_O_2_]^+^, 133.0650[C_9_H_9_O]^+^ |
| 295 | 19.82 | 3-Hydroxybakuchiol or isomer | 273.1849 | C_18_H_24_O_2_ | -0.683 | 273.1847 | 147.0806[M+H-C_8_H_14_O]^+^, 121.0652[M+H-C_10_H_16_O]^+^, 107.0496[M+H-C_11_H_18_O]^+^ |
| 298 | 19.98 | Cyasterone or isomer | 521.3109 | C_29_H_44_O_8_ | 2.139 | 521.3120 | 521.3120[M+H]^+^ |
| 300 | 20.16 | Psoralenol or isomer | 337.1071 | C_20_H_16_O_5_ | 0.029 | 337.1071 | 319.0965[M+H-H_2_O]^+^, 279.0651[M+H-H_2_O-C_3_H_4_]^+^, 137.0235[C_7_H_5_O_3_]^+^ |
| 305^*^ | 20.53 | Schisandrol A | 433.2221 | C_24_H_32_O_7_ | 0.139 | 433.2221 | 415.2115[M+H-H_2_O]^+^, 400.1882[M+H-H_2_O-CH_3_]^+^, 384.1932[M+H-H_2_O-CH_3_O]^+^，369.1700[M+H-H_2_O-CH_3_O-CH_3_]^+^，353.1747[M+H-H_2_O-2OCH_3_]^+^， 338.1512[M+H-H_2_O-2OCH_3_-CH_3_]^+^ |
| 307^*^ | 20.73 | Kaempferol | 287.0550 | C_15_H_10_O_6_ | -0.085 | 287.0550 | 269.0441[M+H-H_2_O]^+^, 259.0602[M+H-CO]^+^, 258.0525[M+H-CHO]^+^, 153.0184[C_7_H_5_O_4_]^+^, 133.0287[C_8_H_5_O_2_]^+^, 121.0287[C_7_H_5_O_2_]^+^, 165.0184[C_8_H_5_O_4_]^+^ |
| 309 | 21.03 | Senkyunolide A or isomer | 193.1223 | C_12_H_16_O_2_ | 0.848 | 193.1225 | 175.1120[M+H-H_2_O]^+^,147.1170[M+H-H_2_O-CO]^+^,137.0599[M+H-C_4_H_8_]^+^ |
| 313 | 21.41 | Gomisin O or isomer | 417.1908 | C_23_H_28_O_7_ | 0.193 | 417.1909 | 399.1801[M+H-H_2_O]^+^, 385.1636[M+H-CH_3_OH]^+^, 373.1649[M+H-C_2_H_4_O]^+^, 357.1334[M+H-C_3_H_8_O]^+^ |
| 314 | 21.47 | Gomisin D | 531.2225 | C_28_H_34_O_10_ | 0.106 | 531.2225 | 485.2176[M+H-CH_2_O_2_]^+^, 401.1597[M+H-C_6_H_10_O_3_]^+^, 383.1490[M+H-C_6_H_10_O_3_-H_2_O]^+^ |
| 315 | 21.51 | Gomisin R | 401.1595 | C_22_H_24_O_7_ | 0.101 | 401.1595 | 383.1489[M+H-H_2_O]^+^, 341.1021[M+H-H_2_O-C_3_H_6_]^+^ |
| 316^*^ | 21.57 | Wogonin | 285.0758 | C_16_H_12_O_5_ | -1.403 | 285.0754 | 270.0531[M+H-CH_3_]^+^, 257.0783[M+H-CO]^+^ |
| 317^*^ | 21.85 | Schisandrol B | 417.1908 | C_23_H_28_O_7_ | 0.121 | 417.1908 | 399.1801[M+H-H_2_O]^+^, 368.1626[M+H-H_2_O-OCH_3_]^+^, 343.1175[M + H-C_4_H_8_-H_2_O]^+^, 307.0962[M+H-2CH_3_-2OCH_3_-H_2_O]^+^ |
| 318 | 21.88 | Bavachin or isomer | 325.1433 | C_20_H_20_O_4_ | -0.202 | 325.1434 | 269.0808[M+H-C_4_H_8_]^+^, 205.0860[M+H-p-hydroxystyrene]^+^, 167.0341[M+H-C_4_H_8_-H_2_O-C_4_H_4_O_2_]^+^, 149.0235[M+H-C_4_H_8_-p-hydroxystyrene]^+^ |
| 319 | 21.91 | Obacunone | 455.2064 | C_26_H_30_O_7_ | -0.021 | 455.2064 | 409.2007[M+H-CH_2_O_2_]^+^,161.0598[C_9_H_10_O_2_]^+^, 105.0700[C_8_H_9_]^+^ |
| 322 | 22.11 | Panaxydol | 261.1849 | C_17_H_24_O_2_ | -0.370 | 261.1848 | 243.1743[M+H-H_2_O]^+^, 105.0339[M+H-C_10_H_10_O]^+^ |
| 323 | 22.18 | Xambioona or isomer | 389.1747 | C_25_H_24_O_4_ | -0.066 | 389.1747 | 239.0702[M+H-C_10_H_14_O]^+^, 137.0234[M+H-C_18_H_20_O]^+^ |
| 324^*^ | 22.25 | Gomisin J | 389.1959 | C_22_H_28_O_6_ | -0.707 | 389.1956 | 374.1736[M+H-CH_3_]^+^, 357.1698[M+H-CH_3_OH]^+^, 287.0913[M+H-C_6_H_14_O]^+^, 227.0703[M+H-C_8_H_18_O_3_]^+^ |
| 325 | 22.38 | Tigloylgomisin H | 501.2483 | C_28_H_36_O_8_ | -0.069 | 501.2483 | 483.2393[M+H-H_2_O]^+^, 401.1950[M+H-H_2_O-C_5_H_6_O]^+^, 370.1762[M+H-H_2_O-C_5_H_6_O-OCH_3_]^+^, 345.1316[M+H-H_2_O-C_5_H_6_O-C_4_H_8_]^+^ |
| 326 | 22.55 | Schisantherin B | 515.2276 | C_28_H_34_O_9_ | -0.270 | 515.2274 | 385.1645[M+H-C_4_H_7_COOH-CH_2_O]^+^, 367.1540 [M+H-C_4_H_7_COOH-CH_2_O-H_2_O]^+^, 353.1377[M+H-C_4_H_7_COOH-CH_2_O-CH_3_OH]^+^ |
| 328 | 22.58 | Corylifolinin isomer | 325.1433 | C_20_H_20_O_4_ | -0.755 | 325.1432 | 269.0807[M+H-C_4_H_8_]^+^, 251.0700[M+H-C_4_H_8_- H_2_O]^+^, 227.0720[M+H-C_4_H_8_-C_2_H_2_O]^+^, 149.0235[M+H-C_4_H_8_-p-hydroxystyrene]^+^ |
| 329 | 22.71 | Hydroxylonchocarpin isomer | 323.1278 | C_20_H_18_O_4_ | 0.633 | 323.1280 | 267.0652[M+H-C_4_H_8_]^+^, 239.0700[M+H-C_4_H_8_-CO]^+^, 211.0758[M+H-C_4_H_8_-2CO]^+^ |
| 330 | 22.78 | Coryfolin | 325.1433 | C_20_H_20_O_4_ | -0.386 | 325.1434 | 269.0810[M+H-C_4_H_8_]^+^, 205.0860[M+H-p-hydroxystyrene]^+^, 167.0340[M+H-C_4_H_8_-H_2_O-C_4_H_4_O_2_]^+^, 149.0235[M+H-C_4_H_8_-p-hydroxystyrene]^+^ |
| 331 | 22.81 | Neobavaisoflavone isomer | 323.1278 | C_20_H_18_O_4_ | -0.296 | 323.1277 | 267.0652[M+H-C_4_H_8_]^+^, 239.0700[M+H-C_4_H_8_-CO]^+^, 211.0758[M+H-C_4_H_8_-2CO]^+^ |
| 332 | 22.81 | Benzoylgomisin H | 523.2326 | C_30_H_34_O_8_ | -4.634 | 523.2302 | 508.2075[M+H-CH_3_]^+^, 493.1846[M+H-2CH_3_]^+^, 401.1608[M+H-C_7_H_6_O_2_]^+^ |
| 333^*^ | 22.84 | Ligustilide | 191.1067 | C_12_H_14_O_2_ | 0.072 | 191.1067 | 173.0963[M+H-H_2_O]^+^, 163.1120[M+H-CO]^+^, 145.1013[M+H-H_2_O-CO]^+^ |
| 334^*^ | 22.88 | Angeloyl gomisin H | 501.2481 | C_28_H_36_O_8_ | -0.308 | 501.2483 | 483.2393[M+H-H_2_O]^+^, 401.1950[M+H-H_2_O-C_5_H_6_O]^+^, 370.1762[M+H-H_2_O-C_5_H_6_O-OCH_3_]^+^, 345.1316[M+H-H_2_O-C_5_H_6_O-C_4_H_8_]^+^ |
| 335 | 22.91 | Linolenic acid | 279.2319 | C_18_H_30_O_2_ | -0.490 | 279.2317 | 261.2209[M+H-H_2_O]^+^, 95.0861[M+H-C_11_H_20_O_2_]^+^ |
| 337 | 23.01 | Chrysophanol or isomer | 255.0652 | C_15_H_10_O_4_ | 0.881 | 255.0654 | 227.0703[M+H-CO]^+^, 209.0594[M+H-CO-H_2_O]^+^ |
| 338 | 23.07 | Psoralidin | 337.1071 | C_20_H_16_O_5_ | -0.356 | 337.1069 | 309.1113[M+H-CO]^+^, 281.1156[M+H-2CO]^+^ |
| 339 | 23.27 | Schisandrin C | 385.1646 | C_22_H_24_O_6_ | -0.195 | 385.1645 | 355.1541[M+H-CH_2_O]^+^, 315.0869[M+H-C_5_H_10_]^+^, 285.0764[M+H-C_5_H_10_-CH_2_O]^+^ |
| 340 | 23.57 | Erythrinin A isomer | 321.1121 | C_20_H_16_O_4_ | 0.045 | 321.1122 | 306.0884[M+H-CH_3_]^+^, 137.0234[M+H-C_13_H_12_O]^+^ |
| 341^*^ | 23.7 | Schisantherin A | 537.2119 | C_30_H_32_O_9_ | -4.186 | 537.2097 | 437.1574[M+Na-C_7_H_6_O_2_]^+^, 415.1752[M+H-C_7_H_6_O_2_]^+^ 371.1490[M+H-C_7_H_6_O_2_-CH_3_CHO]^+^, 356.1255[M+H-C_7_H_6_O_2_-CH_3_CHO-CH_3_]^+^ |
| 342 | 23.77 | Corylin | 321.1121 | C_20_H_16_O_4_ | -0.329 | 321.1120 | 303.1007[M+H-H_2_O]^+^, 279.0652[M+H-C_3_H_6_]^+^, 251.0706[M+H-C_3_H_6_-CO]^+^, 137.0234[M+H-C_13_H_12_O]^+^ |
| 343 | 24.04 | Alloimperatorin | 271.0965 | C_16_H_14_O_4_ | -1.090 | 271.0962 | 256.0728[M+H-CH_3_]^+^, 228.0775[M+H-CH_3_-CO]^+^,210.0684[M+H-CH_3_-CO-H_2_O]^+^ |
| 344 | 24.14 | Gomisin G | 537.2119 | C_30_H_32_O_9_ | -4.652 | 537.2094 | 437.1571[M+Na-C_7_H_6_O_2_]^+^, 415.1752[M+H-C_7_H_6_O_2_]^+^, 371.1491[M+H-C_7_H_6_O_2_-CH_3_CHO]^+^, 356.1261[M+H-C_7_H_6_O_2_-CH_3_CHO-CH_3_]^+^ |
| 345 | 24.37 | Gomisin K_1_ or isomer | 403.2115 | C_23_H_30_O_6_ | 0.161 | 403.2116 | 388.1883[M+H-CH_3_]^+^, 371.1853[M+H-CH_3_-OH]^+^, 356.1609[M+H-CH_3_-OH-CH_3_]^+^, 340.1670[M+H-CH_3_-OH-CH_3_O]^+^ |
| 346 | 24.54 | Gomisin L_1_ or isomer | 387.1802 | C_22_H_26_O_6_ | 0.065 | 387.1802 | 372.1572[M+H-CH_3_]^+^, 357.1692[M+H-CH_2_O]^+^, 356.1617[M+H-OCH_3_]^+^, 329.1759[M+H-C_2_H_2_O_2_]^+^, 325.1431[M+H-2CH_3_OH]^+^ |
| 348 | 24.64 | Bavachinin A | 339.1591 | C_21_H_22_O_4_ | 0.013 | 339.1591 | 283.0964[M+H-C_4_H_8_]^+^, 271.0965[M+H-C_5_H_8_]^+^, 219.1017[M+H-p-hydroxystyrene]^+^ |
| 349 | 24.81 | Licarin A | 327.1591 | C_20_H_22_O_4_ | 0.013 | 327.1591 | 309.1482[M+H- H_2_O]^+^, 271.0966[M+H-CH_3_-C_3_H_5_]^+^ |
| 350 | 24.84 | 8-geranyloxypsoralen | 339.1591 | C_21_H_22_O_4_ | -0.076 | 339.1591 | 283.0964[M+H-C_4_H_8_]^+^, 271.0966[M+H-C_5_H_8_]^+^, 219.1018[M+H-C_8_H_8_O]^+^ |
| 353 | 25.07 | Senkyunolide P or isomer | 381.2060 | C_24_H_28_O_4_ | 0.037 | 381.2061 | 279.1384[M+H-H_2_O-CO-C_4_H_8_]^+^, 191.1069[M+H-C_12_H_14_O_2_]^+^ |
| 355^*^ | 25.74 | Schizandrin A | 417.2272 | C_24_H_32_O_6_ | 0.299 | 417.2273 | 402.2037[M+H-CH_3_]^+^, 386.2092[M+H-CH_3_-O]^+^, 347.1488[M+H-C_5_H_10_]^+^, 316.1307[M+H-CH_3_-O-C_5_H_10_]^+^, 301.1072[M+H-CH_3_-O-C_5_H_10_-CH_3_]^+^, 285.1120[M+H-CH_3_-O-C_5_H_10_-OCH_3_]^+^ |
| 356 | 25.78 | Gemmazone | 219.1743 | C_15_H_22_O | 0.311 | 219.1744 | 201.1639[M+H-H_2_O]^+^, 191.1793[M+H-CO]^+^, 163.1119[M+H-C_4_H_8_]^+^, 121.1015[M+H-C_6_H_10_O]^+^ |
| 357 | 25.84 | Calarene | 205.1951 | C_15_H_24_ | 0.257 | 205.1951 | 149.1326[M+H-C_4_H_8_]^+^, 135.1170[M+H-C_5_H_10_]^+^ |
| 358 | 25.88 | Corylifolinin | 325.1433 | C_20_H_20_O_4_ | -0.110 | 325.1434 | 269.0809[M+H-C_4_H_8_]^+^, 205.0865[M+H-p-hydroxystyrene]^+^, 167.0341[M+H-C_4_H_8_-H_2_O-C_4_H_4_O_2_]^+^, 149.0235[M+H-C_4_H_8_-p-hydroxystyrene]^+^ |
| 360 | 26.04 | Angelicide or isomer | 381.2060 | C_24_H_28_O_4_ | -0.042 | 381.2060 | 335.2004[M+H-H_2_O-CO]^+^, 191.1068[M+H-C_12_H_14_O_2_]^+^ |
| 361 | 26.41 | Corylifol A | 391.1904 | C_25_H_26_O_4_ | -0.501 | 391.1902 | 321.1119[M+H-C_5_H_10_]^+^, 267.0653[M+H-C_9_H_16_]^+^, 239.0704[M+H-C_9_H_16_-CO]^+^, 211.0757[M+H-C_9_H_16_-2CO]^+^ |
| 362^*^ | 26.44 | Levistilide A | 381.2060 | C_24_H_28_O_4_ | -0.199 | 381.2060 | 191.1068[M+H-C_12_H_14_O_2_]^+^, 173.0962[M+H-C_12_H_14_O_2_-H_2_O]^+^, 149.0598[M+H-C_12_H_14_O_2_-C_3_H_6_]^+^, 135.0441[M+H-C_12_H_14_O_2_-C_4_H_8_]^+^ |
| 363^*^ | 26.78 | Schisandrin B | 401.1959 | C_23_H_28_O_6_ | 0.212 | 401.1960 | 386.1720[M+H-CH_3_]^+^, 370.1774[M+H-CH_3_O]^+^, 331.1173[M+H-CH_3_-C_4_H_7_]^+^, 316.0932[M+H-2CH_3_-C_4_H_7_]^+^, 300.0991[M+H-2CH_3_-C_4_H_7_-O]^+^,285.0758[M+H-2CH_3_-C_4_H_7_-2O]^+^ |
| 364 | 27.29 | Benzoylgomisin O or isomer | 521.2170 | C_30_H_32_O_8_ | -4.133 | 521.2148 | 399.1804[M+H-C_6_H_5_COOH-C_3_H_6_]^+^, 369.1704[M+H-C_6_H_5_COOH-CH_2_O]^+^, 357.1332[M+H-C_6_H_5_COOH-C_3_H_6_]^+^, 343.1177 [M+H-C_6_H_5_COOH-C_4_H_8_]^+^ |
| 366 | 28.74 | 13-docosenamide | 338.3417 | C_22_H_43_NO | -0.566 | 338.3416 | 321.3151[M+H-NH_3_]^+^, 303.3047[M+H-NH_3_O]^+^ |

Note: t_R_: retention time; *: compound identified by compared with the reference standard

**Supplementary Table S2. Identification of chemical compounds of HSD by UPLC-Q Exactive-Orbitrap HRMS in negative ion mode**

| Peak  No. | tR  (min) | Proposed compounds | Predicted mass  (Da) | Molecular formula | Error  (ppm) | Observed mass  (Da) | Major fragments |
| --- | --- | --- | --- | --- | --- | --- | --- |
| 2 | 0.77 | Raffinose | 503.1618 | C_18_H_32_O_16_ | 3.058 | 503.1622 | 341.1092[M-H-Glc]^-^, 179.0562[M-H-2Glc]^-^, 161.0457[Glc-H]^-^ |
| 4 | 0.8 | Palatinose | 341.1089 | C_12_H_22_O_11_ | 3.612 | 341.1091 | 179.0561[M-H-Glc]^-^, 161.0455[M-H-Glc-H_2_O]^-^, 101.0244[M-H-C_8_H_16_O_8_]^-^ |
| 5 | 0.8 | Stachyose | 665.2146 | C_24_H_42_O_21_ | 2.323 | 665.2150 | 503.1632[M-H-Glc]^-^, 485.1508[M-H-Glc-H_2_O]^-^, 341.1089[M-H-2Glc]^-^, 179.0561[M-H-3Glc]^-^ |
| 6 | 0.8 | Mannitol | 181.0718 | C_6_H_14_O_6_ | 6.050 | 181.0718 | 163.0613[M-H-H_2_O]^-^ |
| 7 | 0.83 | Sucrose | 341.1089 | C_12_H_22_O_11_ | 3.173 | 341.1089 | 179.0561[M-H-Glc]^-^,161.0455[M-H-Glc-H_2_O]^-^,119.0349[M-H-Glc-CH_2_OH-CHO]^-^,89.0244[M-H-Glc-O-CH_2_OH-CHOH]^-^ |
| 8 | 0.83 | Gluconic acid | 195.0510 | C_6_H_12_O_7_ | 5.080 | 195.0509 | 177.0405[M-H-H_2_O]^-^, 159.0298[M-H-2H_2_O]^-^ |
| 10 | 0.86 | Quinic acid | 191.0561 | C_7_H_12_O_6_ | 5.786 | 191.0561 | 173.0447[M-H-H_2_O]^-^, 127.0402[M-H-H_2_O-COOH]^-^ |
| 14 | 0.93 | Allantoin | 157.0367 | C_4_H_6_N_4_O_3_ | 6.390 | 157.0366 | 157.0366[M-H]^-^ |
| 15 | 0.93 | D-glucaric acid | 209.0303 | C_6_H_10_O_8_ | 5.245 | 209.0303 | 191.0198[M-H-H_2_O]^-^ |
| 23 | 1.14 | Citric acid | 191.0197 | C_6_H_8_O_7_ | 5.502 | 191.0197 | 173.0090[M-H-H_2_O]^-^, 129.0193[M-H-H_2_O-CO_2_]^-^,111.0088[M-H-H_2_O-COOH-CO]^-^ |
| 26^*^ | 1.3 | Rehmannioside D | 731.2240 | C_27_H_42_O_20_ | 2.600 | 731.2260 | 731.2260[M+COOH]^-^, 685.2216[M-H]^-^ |
| 28^*^ | 1.74 | Gallic acid | 169.0142 | C_7_H_6_O_5_ | 5.977 | 169.0142 | 125.0244[M-H-CO_2_]^-^, 97.0295[M-H-C_3_H_4_O_3_]^-^, 69.0347[M-H-CO_2_-2CO]^-^ |
| 29 | 1.77 | Ajugol | 393.1391 | C_15_H_24_O_9_ | 1.362 | 393.1405 | 347.1349[M-H]^-^, 185.0823[M-H-Glc]^-^, 167.0713[M-H-Glc-H_2_O]^-^ |
| 30 | 1.77 | 3-Hydroxymethyl-2-furfural | 125.0244 | C_6_H_6_O_3_ | 1.029 | 125.0244 | 107.0134[M-H-H_2_O]^-^, 97.0295[M-H-CO]^-^ |
| 34^*^ | 2.47 | Geniposidic acid | 373.1140 | C_16_H_22_O_10_ | 3.181 | 373.1141 | 211.0612[M-H-Glc]^-^,193.0500[M-H-Glc-H_2_O]^-^ ,167.0713[M-H-Glc-CO_2_]^-^,149.0607[M-H-Glc-CO_2_-H_2_O]^-^, 123.0451[M-H-Glc-CO_2_-H_2_O-C_2_H_2_]^-^ |
| 37 | 2.71 | Protocatechuic acid-O-glucoside | 315.0722 | C_13_H_16_O_9_ | 4.512 | 315.0725 | 153.0194[M-H-Glc]^-^, 109.0295[M-H-Glc-CO_2_]^-^ |
| 38 | 2.74 | Aucubin | 391.1235 | C_15_H_22_O_9_ | 3.483 | 391.1249 | 345.1176[M-H]^-^, 183.0667[M-H-Glc]^-^, 165.0553[M-H-Glc-H_2_O]^-^,153.0557[M-H-Glc-CH_2_OH]^-^ |
| 39 | 2.84 | Vanillic acid or isomer | 167.0350 | C_8_H_8_O_4_ | 6.135 | 167.0349 | 152.0115[M-H-CH_3_]^-^, 137.0240[M-H-CH_2_O]^-^, 123.0451[M-H-CO_2_]^-^, 122.0337[M-H-COOH]^-^ |
| 40 | 2.94 | Decaffeoyl-verbascoside isomer | 461.1664 | C_20_H_30_O_12_ | 1.637 | 461.1670 | 315.1086[M-H-Rha]^-^, 153.0559[M-H-Rha-Glc]^-^, 135.0452[M-H-Rha-Glc-H_2_O]^-^ |
| 41 | 3.01 | Vanillin | 151.0401 | C_8_H_8_O_3_ | 7.477 | 151.0401 | 123.0453[M-H-CO]^-^, 123.0453[M-H-CO_2_]^-^ |
| 42 | 3.08 | Mussarnosidic acid | 375.1297 | C_16_H_24_O_10_ | 3.403 | 375.1299 | 213.0771[M-H-Glc]^-^, 169.0870[M-H-Glc-CO_2_]^-^, 151.0764[M-H-Glc-CO_2_-H_2_O]^-^ |
| 43 | 3.15 | Decaffeoyl-acteoside | 461.1664 | C_20_H_30_O_12_ | 3.225 | 461.1668 | 315.1080[M-H-Rha]^-^, 153.0558[M-H-Rha-Glc]^-^, 135.0452[M-H-Rha-Glc-H_2_O]^-^ |
| 45 | 3.29 | 8-epiloganic acid | 375.1297 | C_16_H_24_O_10_ | 3.483 | 375.1299 | 213.0771[M-H-Glc]^-^, 169.0870[M-H-Glc-CO_2_]^-^, 151.0765[M-H-Glc-CO_2_-H_2_O]^-^ |
| 46 | 3.35 | Dihydroxy-benzoic acid or isomer | 153.0193 | C_7_H_6_O_4_ | 7.351 | 153.0194 | 125.0244[M-H-CO]^-^, 109.0295[M-H-CO_2_]^-^ |
| 47 | 3.39 | Gentisic acid | 153.0193 | C_7_H_6_O_4_ | 7.286 | 153.0193 | 109.0295[M-H-CO_2_]^-^, 108.0217[M-H-COOH]^-^, 91.0189[M-H-CO_2_-H_2_O]^-^ |
| 49 | 3.56 | Veratric acid | 181.0506 | C_9_H_10_O_4_ | 6.378 | 181.0507 | 137.0346[M-H-CO_2_]^-^, 121.0293[M-H-2CH_2_O]^-^ |
| 51 | 4.03 | Cryptochlorogenic acid isomer | 353.0878 | C_16_H_18_O_9_ | 4.139 | 353.0882 | 191.0562[M-H-caffeoyl]^-^, 179.0350[M-H-C_7_H_10_O_5_]^-^,135.0452[M-H-C_8_H_10_O_7_]^-^ |
| 53^*^ | 4.23 | Neochlorogenic acid | 353.0878 | C_16_H_18_O_9_ | 3.856 | 353.0881 | 191.0562[M-H-caffeoyl]^-^, 179.0350[M-H-C_7_H_10_O_5_]^-^, 173.0456[M-H-C_9_H_8_O_4_]^-^, 135.0452[M-H-C_7_H_10_O_5_-CO_2_]^-^, 93.0345[M-H-C_9_H_8_O_4_-2H_2_O-CO_2_]^-^ |
| 54 | 4.24 | Protocatechuic aldehyde | 137.0244 | C_7_H_6_O_3_ | 7.951 | 137.0244 | 119.0140[M-H-H_2_O]^-^, 108.0217[M-H-CHO]^-^,93.0346[M-H-CO_2_]^-^, 81.0346[M-H-2CO]^-^ |
| 55 | 4.27 | 3-O-(4'-O-Caffeoylglucosyl)quinic acid | 515.1406 | C_22_H_28_O_14_ | 3.064 | 515.1411 | 341.0882[M-H-C_7_H_10_O_5_]^-^, 353.0879[M-H-Glc]^-^, 191.0561[M-H-Glc-caffeoyl]^-^, 173.0455[M-H-Glc-caffeoyl-H_2_O]^-^ |
| 56 | 4.27 | Protocatechualdehyde or isomer | 137.0244 | C_7_H_6_O_3_ | 7.951 | 137.0244 | 93.0346[M-H-CO_2_]^-^, 65.0397[M-H-CO_2_-CO]^-^ |
| 57 | 4.37 | Lamiol | 377.1453 | C_16_H_26_O_10_ | 3.571 | 377.1456 | 341.1087[M-H-2H_2_O]^-^, 161.0458[M-H-C_10_H_16_O_5_]^-^ |
| 61 | 4.67 | Kanokside C | 637.2349 | C_27_H_42_O_17_ | 2.674 | 637.2355 | 491.1781[M-H-Glc]^-^, 473.1671[M-H-Glc-H_2_O]^-^, 161.0459[Glc-H]^-^ |
| 63 | 4.91 | 3‑O‑feruloylquinic acid glucoside | 529.1563 | C_23_H_30_O_14_ | 2.472 | 529.1565 | 191.0561[M-H-C_10_H_8_O_3_]^-^, 173.0455[M-C_10_H_10_O_4_]^-^, 134.0373[M-CH_3_-C_8_H_10_O_7_]^-^, 111.0452[M-H-C_10_H_9_O_4_-CO_2_-OH-2H]^-^, 109.0297[M-H-CO_2_-OH-C_10_H_13_O_4_]^-^ |
| 66 | 5.28 | Rehmannioside A | 523.1668 | C_21_H_32_O_15_ | 1.613 | 523.1674 | 523.1668[M-H]^-^ |
| 67 | 5.35 | 5-O-(3'-O-Caffeoylglucosyl)quinic acid | 515.1406 | C_22_H_28_O_14_ | 2.598 | 515.1409 | 353.0892[M-H-Glc]^-^, 341.0875[M-H-C_7_H_10_O_5_]^-^, 323.0774[M-H-C_7_H_10_O_5_-H_2_O]^-^, 191.0561[M-H-Glc-caffeoyl]^-^, 173.0457[M-H-Glc-caffeoyl-H_2_O]^-^ |
| 68^*^ | 5.41 | Phthalic acid | 165.0193 | C_8_H_6_O_4_ | 6.574 | 165.0193 | 121.0296[M-H-CO_2_]^-^ |
| 69 | 5.59 | P-Hydroxybenzoic Acid or isomer | 137.0244 | C_7_H_6_O_3_ | 7.951 | 137.0244 | 93.0346[M-H-CO_2_]^-^, 65.0396[M-H-CO_2_-CO]^-^ |
| 72 | 5.72 | Darendoside B | 475.1821 | C_21_H_32_O_12_ | 3.004 | 475.1824 | 329.1245[M-H-Rha]^-^, 311.1138[M-H-Rha-H_2_O]^-^, 167.0718[M-H-Rha-Glc]^-^, 152.0474[M-H-Rha-Glc-CH_3_]^-^ |
| 76 | 5.89 | Esculetin | 177.0193 | C_9_H_6_O_4_ | 6.354 | 177.0194 | 149.0246[M-H-CO]^-^, 133.0295[M-H-CO_2_]^-^, 105.0346[M-H-CO_2_-CO]^-^ |
| 77^*^ | 6.06 | Cianidanol | 289.0718 | C_15_H_14_O_6_ | 4.689 | 289.0720 | 245.0821[M-H-CO_2_]^-^, 203.0717[M-H-C_3_H_2_O_3_]^-^, 123.0452[M-H-C_8_H_6_O_4_]^-^, 109.0295[M-H-C_9_H_8_O_4_]^-^ |
| 79^*^ | 6.23 | Procyanidin B1 | 577.1351 | C_30_H_26_O_12_ | 3.132 | 577.1359 | 425.0894[M-H-C_8_H_8_O_3_]^-^, 407.0775[M-H-C_8_H_8_O_3_-H_2_O]^-^, 289.0721[C_15_H_13_O_6_]^-^, 125.0245[C_6_H_5_O_3_]^-^ |
| 81 | 6.27 | 3-O-feruloylquinic acid | 367.1035 | C_17_H_20_O_9_ | 3.245 | 367.1036 | 367.1037[M-H]^-^ |
| 82^*^ | 6.3 | Cryptochlorogenic acid | 353.0878 | C_16_H_18_O_9_ | 3.856 | 353.0880 | 191.0561[M-H-caffeoyl]^-^, 179.0350[M-H-C_7_H_10_O_5_]^-^, 173.0455[M-H-C_9_H_8_O_4_]^-^, 135.0452[M-H-C_7_H_10_O_5_-CO_2_]^-^, 93.0346[M-H-C_9_H_8_O_4_-2H_2_O-CO_2_]^-^ |
| 83 | 6.37 | Quercetin-3-o-galactoside-7-o-glucoside | 625.1410 | C_27_H_30_O_17_ | 2.870 | 625.1417 | 463.0892[M-H-Glc]^-^, 301.0354[M-H-Glc-C_6_H_10_O_5_]^-^ |
| 84 | 6.47 | Rhamnopyranosyl vanilloyl | 313.0929 | C_14_H_18_O_8_ | 1.316 | 313.0931 | 167.0350[M-H-Rha]^-^, 152.0115[M-H-Rha-CH_3_]^-^, 123.0452 [M-H-Rha-CO_2_]^-^ |
| 85^*^ | 6.5 | Chlorogenic acid | 353.0878 | C_16_H_18_O_9_ | 3.516 | 353.0880 | 191.0562[M-H-caffeoyl]^-^, 179.0350[M-H-C_7_H_10_O_5_]^-^, 173.0456[M-H-C_9_H_8_O_4_]^-^, 161.0244[M-H-C_7_H_10_O_5_- H_2_O]^-^,155.0347[M-H-C_9_H_8_O_4_-H_2_O]^-^, 135.0452[M-H-C_7_H_10_O_5_-CO_2_]^-^, 93.0345[M-H-C_9_H_8_O_4_-2H_2_O-CO_2_]^-^ |
| 88 | 6.63 | 3-O-Caffeoylqunic acid | 353.0878 | C_16_H_18_O_9_ | 3.601 | 353.0880 | 191.0562[M-H-caffeoyl]^-^, 179.0351[M-H-C_7_H_10_O_5_]^-^, 173.0455[M-H-caffeoyl- H_2_O]^-^ |
| 89 | 6.67 | Quercetin-3-O-caffeoylgalactoside | 625.1410 | C_27_H_30_O_17_ | 2.774 | 625.1417 | 463.0890[M-H-caffeoyl]^-^, 301.0354[M-H-caffeoyl-Glc]^-^, 151.003[M-H-C_8_H_6_O_3_]^-^ |
| 91 | 6.77 | Sec-hydroxyaeginetic acid | 283.1551 | C_15_H_24_O_5_ | 4.590 | 283.1553 | 238.1533[M-H-COOH]^-^, 183.1026[M-H-C_5_H_8_O_2_]^-^ |
| 92 | 6.84 | Syringic acid-4-O-α-L-rhamnoside | 343.1035 | C_15_H_20_O_9_ | 1.371 | 343.1037 | 197.0456[M-H-Rha]^-^, 182.0220[M-H-Rha-CH_3_]^-^, 166.9988[M-H-Rha-2CH_3_]^-^, 153.0557[M-H-Rha-CO_2_]^-^ |
| 93 | 6.91 | Cistanoside F or isomer | 487.1462 | C_21_H_28_O_13_ | 3.331 | 487.1462 | 341.1113[M-H-Rha]^-^, 325.0937[M-H-caffeoyl]^-^, 179.0347[M-H-Rha-Glu]^-^, |
| 94^*^ | 7.05 | Caffeic acid | 179.0350 | C_9_H_8_O_4_ | 6.171 | 179.0350 | 135.0452[M-H-CO_2_]^-^ |
| 96 | 7.18 | Rehmapicroside | 345.1555 | C_16_H_26_O_8_ | 4.044 | 345.1558 | 301.1675[M-H-CO_2_]^-^, 285.13461[M-H-CO_2_-CH_4_]^-^, 183.10272[M-H-Glc]^-^ |
| 97 | 7.19 | 4-O-Caffeoylqunic acid | 353.0878 | C_16_H_18_O_9_ | 4.479 | 353.0883 | 191.0562[M-H-caffeoyl]^-^, 179.0343[M-H-C_7_H_10_O_5_]^-^, 173.0459[M-H-caffeoyl- H_2_O]^-^ |
| 98^*^ | 7.38 | Epicatechin | 289.0718 | C_15_H_14_O_6_ | 4.274 | 289.0719 | 245.0822[M-H-CO_2_]^-^, 203.0714[M-H-C_3_H_2_O_3_]^-^,123.0452[M-H-C_8_H_6_O_4_]^-^, 109.0295[M-H-C_9_H_8_O_4_]^-^ |
| 99 | 7.41 | Cistantubuloside C1 or isomer | 801.2459 | C_35_H_46_O_21_ | 2.553 | 801.2468 | 783.2367[M-H-H_2_O]^-^, 639.2153[M-H-Glc]^-^, 621.2025[M-H-Glc-H_2_O]^-^ |
| 100 | 7.48 | Rumejaposide D or isomer | 449.1089 | C_21_H_22_O_11_ | 3.078 | 449.1092 | 431.0987[M-H-H2O]-, 287.0564[M-H-Glc]^-^, 269.0458[M-H-Glc-H_2_O]^-^, 151.0038[M-H-Glc-C_8_H_8_O_2_]-, 259.0613[M-H-Glc-CO]^-^ |
| 101 | 7.51 | Rehmapicrogenin | 183.1027 | C_10_H_16_O_3_ | 1.079 | 183.1027 | 139.1128[M-H-CO_2_]^-^, |
| 103 | 7.55 | Kaempferol-dihexoside | 609.1461 | C_27_H_30_O_16_ | 2.920 | 609.1468 | 447.0939[M-H-Glc]^-^, 285.0407[M-H-2Glc]^-^, 255.0301[M-H-2Glc-CH_2_O]^-^ |
| 105 | 7.62 | Psoralenoside | 365.0878 | C_17_H_18_O_9_ | 3.483 | 365.0880 | 203.0352[M-H-Glc]^-^, 159.0452[M-H-Glc-CO_2_]^-^ |
| 107 | 8.06 | Alaschanioside A | 537.1977 | C_26_H_34_O_12_ | 3.755 | 537.1987 | 327.1235[M-H-Glc-OH-OCH_3_]^-^ |
| 110 | 8.27 | Isopsoralenoside | 365.0878 | C_17_H_18_O_9_ | 3.811 | 365.0881 | 203.0351[M-H-Glc]^-^, 159.0451[M-H-Glc-CO_2_]^-^ |
| 111 | 8.3 | Purpureaside C | 785.2510 | C_35_H_46_O_20_ | 2.725 | 785.2520 | 623.2209[M-H-caffeoyl]^-^, 461.1672[M-H-caffeoyl-Glc]^-^, 315.1083[M-H-caffeoyl-Glc-Rha]^-^, 153.0554[M-H-caffeoyl-2Glc-Rha]^-^ |
| 113 | 8.5 | Acetyl-epicatechin-O-glucoside | 493.1351 | C_23_H_26_O_12_ | 3.361 | 493.1357 | 330.0745[M-H-C_6_H_11_O_5_]^-^, 255.0664[M-H-C_6_H_10_O_5_-C_2_H_2_O_2_-H_2_O]^-^, 227.0713[M-H-C_6_H_10_O_5_-C_2_H_2_O_2_-H_2_O-CO]^-^ |
| 115 | 8.61 | Polypodine B | 541.3007 | C_27_H_44_O_8_ | 3.373 | 541.3026 | 495.2969[M-H]^-^ |
| 116^*^ | 8.71 | Echinacoside | 785.2510 | C_35_H_46_O_20_ | 3.196 | 785.2524 | 623.2195[M-H-caffeoyl]^-^, 477.1642[M-H-caffeoyl-Rha]^-^, 461.1670[M-H-caffeoyl-Glc]^-^, 315.1101[M-H-caffeoyl-Rha-Glc]^-^, 179.0349[M-H-2Glc-Rha-C_8_H_8_O_2_]^-^, 161.0244[M-H-2Glc-Rha-C_8_H_8_O_2_-H_2_O]^-^ |
| 118 | 8.77 | 4-p-coumaroylquinicacid | 337.0929 | C_16_H_18_O_8_ | 4.527 | 337.0933 | 191.0562[M-H-C_9_H_6_O_2_]^-^, 173.0456[M-H-C_9_H_6_O_2_-H_2_O]^-^,163.0402[M-H-C_7_H_10_O_5_]^-^, 119.0502[M-H-C_7_H_10_O_5_-CO_2_]^-^ |
| 119 | 8.87 | 4-O-feruloylquinic acid | 367.1035 | C_17_H_20_O_9_ | 2.891 | 367.1034 | 191.0562[M-H-C_10_H_8_O_3_]^-^, 173.0456[M-H-C_10_H_8_O_3_-H_2_O]^-^, 134.0374[M-H-CH_3_-C_8_H_10_O_7_]^-^ |
| 121 | 8.93 | 5-O-feruloylquinic acid isomer | 367.1035 | C_17_H_20_O_9_ | 2.810 | 367.1034 | 191.0562[M-H-C_10_H_8_O_3_]^-^, 173.0455[M-H-C_10_H_8_O_3_-H_2_O]^-^,134.0373[M-H-CH_3_-C_8_H_10_O_7_]^-^, 111.0452[M-H-C_10_H_9_O_4_-CO_2_-OH]^-^ |
| 122 | 9 | 5-O-feruloylquinic acid | 367.1035 | C_17_H_20_O_9_ | 3.082 | 367.1035 | 191.0561[M-H-C_10_H_8_O_3_]^-^, 173.0456 [M-H-C_10_H_8_O_3_-H_2_O]^-^ |
| 123 | 9.03 | Rehmapicroside isomer | 345.1555 | C_16_H_26_O_8_ | 3.523 | 345.1556 | 301.1660[M-H-CO_2_]^-^, 183.1020[M-H-Glc]^-^, 161.0457[M-H-C_10_H_16_O_3_]^-^ |
| 126 | 9.06 | Β-hydroxyacteoside | 639.1931 | C_29_H_36_O_16_ | 3.143 | 639.1940 | 621.1832[M-H-H_2_O]^-^, 477.1610[M-H-caffeoyl]^-^, 459.1509[M-H-caffeoyl-H_2_O]^-^ |
| 127 | 9.43 | Jionoside A1/A2 | 799.2666 | C_36_H_48_O_20_ | 3.190 | 799.2681 | 623.2199[M-H-C_10_H_8_O_3_]^-^, 605.2115[M-H-C_10_H_8_O_3_-H_2_O]^-^, 461.1649[M-H-C_10_H_8_O_3_-Glc]^-^, 153.0558 [M-H-C_10_H_8_O_3_-2Glc-Rha]^-^ |
| 130 | 9.64 | Kankanoside A | 345.1555 | C_16_H_26_O_8_ | 3.870 | 345.1557 | 183.1021[M-H-Glc]^-^, 165.0921[M-H-Glc-H_2_O]^-^ |
| 133 | 9.77 | Cistanoside A or isomer | 799.2666 | C_36_H_48_O_20_ | 2.803 | 799.2678 | 637.2407[M-H-caffeoyl]^-^, 623.2198[M-H-OCH_3_-Rha]^-^ |
| 134 | 9.81 | Poliumoside | 769.2561 | C_35_H_46_O_19_ | 2.606 | 769.2570 | 623.2194[M-H-caffeoyl]^-^, 605.2090[M-H-caffeoyl- H_2_O]^-^, 461.1643[M-H-caffeoyl-Rha]^-^ |
| 135 | 9.87 | Lariciresinol glucoside or isomer | 521.2028 | C_26_H_34_O_11_ | 2.076 | 521.2028 | 359.1510[M-H-Glc]^-^, 329.1398[M-H-Glc-CH_2_O]^-^ |
| 136^*^ | 9.87 | Plantamajoside | 639.1931 | C_29_H_36_O_16_ | 3.236 | 639.1940 | 477.1618[M-H-caffeoyl]^-^, 315.1083[M-H-caffeoyl-Glc]^-^, 179.0349[Caffeic acid-H]^-^, 161.0244[Caffeicacid-H-H_2_O]^-^ ,153.0555[M-H-caffeoyl-2Glc]^-^,135.0451[M-H-C_9_H_6_O_3_-2Glc-H_2_O]^-^ |
| 138 | 9.94 | Campneoside II | 639.1931 | C_29_H_36_O_16_ | 2.955 | 639.1939 | 621.1711[M-H- H_2_O]^-^, 179.0349[M-H- H_2_O -Glc-Rha-Hydroxytyrosol]^-^, 161.0244[M-H- H_2_O -Glc-Rha-Hydroxytyrosol-H_2_O]^-^, 135.0451[M-H- H_2_O -Glc-Rha-Hydroxytyrosol-CO_2_]^-^ |
| 139 | 10.04 | 6-Methoxyl-2-Acetyl-3-methyljuglone-8-O-β-D-glucoside | 421.1140 | C_20_H_22_O_10_ | 3.459 | 421.1144 | 259.0612[M-H-Glc]^-^, 241.0505[M-H-Glc-H_2_O]^-^, 213.0559[M-H-Glc-H_2_O-CO]^-^ |
| 142^*^ | 10.21 | Ferulic acid | 193.0506 | C_10_H_10_O_4_ | 5.826 | 193.0505 | 178.0271[M-H-CH_3_]^-^, 149.0608[M-H-CO_2_]^-^, 134.0374[M-H-CO_2_-CH_3_]^-^ |
| 144 | 10.28 | Tetrahydroxystilbene-O-di-glucoside | 567.1719 | C_26_H_32_O_14_ | 3.135 | 567.1726 | 243.0662[M-H-2Glc]^-^, 225.0562[M-H-2Glc-H_2_O]^-^, 197.0606 [M-H-2Glc-H_2_O-CO]^-^ |
| 145 | 10.28 | Plantagoside | 465.1038 | C_21_H_22_O_12_ | 3.284 | 465.1043 | 303.0515[M-H-Glc]^-^, 151.0402[M-H-Glc-C_7_H_4_O_4_]^-^, |
| 147 | 10.31 | Conicaoside | 551.2134 | C_27_H_36_O_12_ | 2.952 | 551.2139 | 389.1615[M-H-Glc]^-^, 359.1504[M-H-Glc-OCH_3_]^-^, 344.1268[M-H-C_6_H_10_O_5_-OCH_3_-CH_3_]^-^, 195.0662[M-H-Glc-C_11_H_14_O_3_]^-^ |
| 149 | 10.53 | 25R-Achyranthes bidentata | 525.3058 | C_27_H_44_O_7_ | 2.971 | 525.3074 | 479.3016[M-H]^-^,319.1917[M-H-C_8_H_16_O_3_]^-^,159.1028[M-H-C_8_H_16_O_3_-C_11_H_12_O]^-^ |
| 152 | 10.59 | Tubuloside A | 827.2615 | C_37_H_48_O_21_ | 3.246 | 827.2631 | 665.2318[M-H-caffeoyl]^-^, 623.2224[M-H-caffeoyl-C_2_H_2_O]^-^, 477.1624[M-H-caffeoyl-C_2_H_2_O-Glc]^-^ |
| 153 | 10.73 | 25S-Achyranthes bidentata | 525.3058 | C_27_H_44_O_7_ | 2.628 | 525.3072 | 479.3016[M-H]^-^,319.1901[M-H-C_8_H_16_O_3_]^-^,159.1026[M-H-C_8_H_16_O_3_-C_11_H_12_O]^-^ |
| 157 | 10.93 | Jionoside B1/B2 | 813.2823 | C_37_H_50_O_20_ | 2.656 | 813.2833 | 637.2366[M-H-C_10_H_8_O_3_]^-^, 619.2274[M-H-C_10_H_8_O_3_-H_2_O]^-^, 175.0400[M-H-C_9_H_11_O_3_-2Glc-Rha]^-^, 167.0720 [M-H-C_10_H_8_O_3_-2Glc-Rha]^-^ |
| 158^*^ | 10.96 | Acteoside | 623.1981 | C_29_H_36_O_15_ | 3.776 | 623.1994 | 461.1646 (M-H-caffeoyl)^-^, 315.1086 (M-H-caffeoyl-Rha)^-^, 179.0348 (Caffeoyl-H)^-,^ 161.0244 (Caffeoyl-H-H_2_0)^-^, 135.0452 (M-H-caffeoyl-Rha-Glc-H_2_O)^-^ |
| 160 | 11.06 | Isoferulic acid | 193.0506 | C_10_H_10_O_4_ | 5.515 | 193.0506 | 178.0272[M-H-CH_3_]^-^, 149.0608[M-H-CO_2_]^-^, 134.0374[M-H-CO_2_-CH_3_]^-^ |
| 162 | 11.06 | Pinoresinol glucoside | 519.1872 | C_26_H_32_O_11_ | 2.604 | 519.1874 | 357.1346[M-H-Glc]^-^, 342.1105[M-H-Glc-CH_3_]^-^ |
| 163 | 11.2 | Syringaresinol glucoside | 579.2083 | C_28_H_36_O_13_ | 2.836 | 579.2089 | 417.1557[M-H-Glc]^-^, 402.1321[M-H-Glc-CH_3_]^-^, 387.1088[M-H-Glc-2CH_3_]^-^ |
| 164 | 11.23 | Cistanoside K or isomer | 679.2244 | C_32_H_40_O_16_ | 3.060 | 679.2253 | 517.1718[M-H-caffeoyl]^-^, 499.1613[M-H-caffeoyl-H_2_O]^-^, 355.1196[M-H-caffeoyl-Glc]^-^, |
| 166^*^ | 11.3 | Tetrahydroxystilbene glucoside | 405.1191 | C_20_H_22_O_9_ | 3.311 | 405.1194 | 243.0663[M-H-Glc]^-^, 225.0559[M-H-Glc-H_2_O]^-^, 215.0714[M-H-Glc-CO]^-^, 137.0244[M-H-Glc-C_7_H_5_O]^-^, 93.0346[M-H-Glc-C_7_H_5_O-CO]^-^ |
| 167 | 11.33 | 3,4,5,4’-tetrahydroxystilbene isomer | 243.0663 | C_14_H_12_O_4_ | 2.117 | 243.0657 | 225.0559[M-H-H_2_O]^-^, 215.0714[M-H-CO]^-^, 197.0607[M-H-H_2_O-CO]^-^ |
| 168 | 11.4 | Quercetin-3-O-apiosyl-(1→2)-galactoside | 595.1305 | C_26_H_28_O_16_ | 2.317 | 595.1307 | 300.0277[M-H-C_11_H_19_O_9_]^-^, 273.0413[M-H-C_11_H_19_O_9_-CO]^-^, 151.0036[M-H-C_11_H_19_O_9_-C_8_H_6_O_3_]^-^ |
| 169 | 11.4 | Polygonumosides C | 827.2404 | C_40_H_44_O_19_ | 1.045 | 827.2402 | 421.1192[M-H-C_20_H_22_O_9_]^-^, 259.0609[M-H-C_20_H_22_O_9_-Glc]^-^, 241.0506[M-H-C_20_H_22_O_9_-Glc- H_2_O]^-^ |
| 170 | 11.46 | Calceolarioside B | 477.1402 | C_23_H_26_O_11_ | 2.289 | 477.1402 | 315.1088[M-H-caffeoyl]^-^，179.03502[CA-H]^-^, 161.0244[CA-H-H_2_O]^-^，133.0294[CA-H-H_2_O-CO]^-^ |
| 171 | 11.46 | Azelaic acid | 187.0976 | C_9_H_16_O_4_ | 5.423 | 187.0975 | 169.0870[M-H-H_2_O]^-^, 143.1077[M-H-CO_2_]^-^, 125.0972[M-H-H_2_O-COOH]^-^ |
| 172 | 11.56 | Epicatechin-O-gallate | 441.0827 | C_22_H_18_O_10_ | 3.144 | 441.0830 | 289.0719[M-H-C_7_H_5_O_4_]^-^, 243.0662[M-H-C_7_H_5_O_4_- H_2_O -CO]^-^, 225.0564[M-H-C_7_H_5_O_4_-2 H_2_O -CO]^-^, 169.0142[M-H-C_15_H_12_O_5_]^-^ |
| 173 | 11.56 | Plantainoside D | 639.1931 | C_29_H_36_O_16_ | 3.143 | 639.1940 | 477.1626[M-H-caffeoyl]^-^, 315.1105[M-H-caffeoyl-Glc]^-^, 153.0556[M-H-caffeoyl-2Glc]^-^ |
| 174 | 11.63 | Eucommin A or isomer | 549.1977 | C_27_H_34_O_12_ | 2.890 | 549.1982 | 549.1982[M-H]^-^ |
| 175 | 11.67 | Cuscutoside D | 825.2459 | C_37_H_46_O_21_ | 2.842 | 825.2471 | 663.1934[M-H-Glc]^-^, 603.1715[M-H-Glc-C_2_H_4_O_2_]^-^ |
| 176 | 11.67 | 2′-acetylpoliumoside | 811.2666 | C_37_H_48_O_20_ | 4.413 | 811.2691 | 769.2640[M-H-C_2_H_2_O]^-^, 665.2308[M-H-C_9_H_7_O_3_]^-^, 623.2183[M-H-C_9_H_7_O_3_-C_2_H_2_O]^-^, 477.1628[M-H-C_9_H_7_O_3_-C_2_H_2_O-Rha]^-^ |
| 177 | 12.02 | Acteoside isomer | 623.1981 | C_29_H_36_O_15_ | 2.990 | 623.1989 | 461.1668[M-H-caffeoyl]^-^, 315.1096[M-H-caffeoyl-Rha]^-^, 179.0349[M-H-Rha-Glc-C_8_H_8_O_2_]^-^, 161.0244[M-H-Rha-Glc-C_8_H_8_O_2_-H_2_O]^-^, 135.0452[M-H-Rha-Glc-C_8_H_8_O_2_-CO_2_]^-^ |
| 178 | 12.11 | Kankanoside G or isomer | 607.2032 | C_29_H_36_O_14_ | 2.648 | 607.2037 | 445.1713[M-H-caffeoyl]^-^, 179.0349[M-H-Rha-Glc-C_8_H_8_O_2_]^-^ 161.0244[M-H-Rha-Glc-C_8_H_8_O_2_- H_2_O]^-^, 135.0450[M-H-Rha-Glc-C_8_H_8_O_2_-CO_2_]^-^ |
| 179 | 12.15 | 6-Hydroxy-luteolin-7-O-glucoside | 463.0882 | C_21_H_20_O_12_ | 3.407 | 463.0887 | 301.0356[M-H-Glc]^-^, 273.0407[M-H-Glc-CO]^-^ |
| 180^*^ | 12.18 | Hyperoside | 463.0882 | C_21_H_20_O_12_ | 3.752 | 463.0888 | 301.0356[M-H-Glc]^-^, 255.0299[M-H-Glc-CO- H_2_O]^-^, 151.0036[C_7_H_3_O_4_]^-^, 107.0140[C_6_H_3_O_2_]^-^ |
| 182 | 12.22 | Spiraeoside isomer | 463.0882 | C_21_H_20_O_12_ | 3.687 | 463.0888 | 301.0356[M-H-Glc]^-^, 283.0252 [M-H-Glc-H_2_O]^-^, 255.0299[M-H-Glc-H_2_O-CO]^-^, |
| 183 | 12.22 | Acteoside isomer | 623.1981 | C_29_H_36_O_15_ | 3.086 | 623.1990 | 461.1692[M-H-caffeoyl]^-^, 315.1096[M-H-caffeoyl-Rha]^-^, 179.0349[M-H-Rha-Glc-C_8_H_8_O_2_]^-^, 161.0244[M-H-Rha-Glc-C_8_H_8_O_2_-H_2_O]^-^, 135.0453[M-H-Rha-Glc-C_8_H_8_O_2_-CO_2_]^-^, |
| 184 | 12.28 | Tetrahydroxystilbene-O-(galloyl)-glucoside | 557.1301 | C_27_H_26_O_13_ | 2.195 | 557.1302 | 405.1192[M-H-C_7_H_4_O_4_]^-^, 243.0663[M-H-C_7_H_4_O_4_-Glc]^-^, 225.0559[M-H-C_7_H_4_O_4_-Glc-H_2_O]^-^ |
| 185 | 12.28 | Leucosceptoside A | 637.2138 | C_30_H_38_O_15_ | 2.893 | 637.2145 | 475.1821[M-H-caffeoyl]^-^, 179.0350[CA-H]^-^, 161.0245[CA-H-H_2_O]^-^，133.0294[CA-H-H_2_O-CO]^-^ |
| 186 | 12.32 | Jiocarotenoside A1/A2 | 429.2130 | C_21_H_34_O_9_ | 2.635 | 429.2130 | 267.1603[M-H-Glc]^-^ |
| 187 | 12.42 | Dunnisinoside | 549.1614 | C_26_H_30_O_13_ | 2.682 | 549.1617 | 341.1031[M-H-Glc-CH_2_O_2_]^-^ |
| 188 | 12.46 | Kaempferol 3-apiosyl-(1->2)-glucoside | 579.1355 | C_26_H_28_O_15_ | 3.183 | 579.1363 | 285.0404[M-H-C_11_H_18_O_9_]^-^, 255.0300[M-H-C_11_H_18_O_9_-CH_2_O]^-^ |
| 189 | 12.49 | Cistanoside C | 637.2138 | C_30_H_38_O_15_ | 2.704 | 637.2144 | 315.1129[M-H-caffeoyl-Rha-CH_3_]^-^, 179.0351[M-H-Rha-C_9_H_10_O_2_-Glc]^-^, 153.0559[M-H-caffeoyl-Rha-CH_3_-Glc]^-^, 135.0452 [M-H-Rha-C_9_H_10_O_2_-Glc-CO_2_]^-^ |
| 190^*^ | 12.63 | Rutin | 609.1461 | C_27_H_30_O_16_ | 3.117 | 609.1469 | 300.0273[M-H-Glc-Rha]^-^, 271.0250[M-H-Glc-Rha--H-CO]^-^, 257.0457[M-H-Glc-Rha-CO_2_]^-^, 243.0300[M-H-Glc-Rha--H-CO-CO]^-^, 151.0035[C_7_H_3_O_4_]^-^ |
| 192 | 12.8 | Rhoifolin | 577.1563 | C_27_H_30_O_14_ | 2.804 | 577.1568 | 413.0890[M-H-Rha-H_2_O]^-^, 269.0457[M-H-Glc-Rha]^-^, 225.0552[M-H-Glc-Rha-CO_2_]^-^, 183.0451[C_12_H_7_O_2_]^-^ |
| 193 | 12.95 | 2-acetylacteoside | 665.2087 | C_31_H_38_O_16_ | 3.080 | 665.2097 | 623.1987[M-H-acetyl]^-^，503.1784[M-H-caffeoyl]^-^, 461.1665[M-H-acetyl-caffeoyl]^-^, 179.0351[CA-H]^-^, 161.0245[CA-H-H_2_O]^-^，133.0294[CA-H-H_2_O-CO]^-^ |
| 194 | 12.98 | Astragalin isomer | 447.0933 | C_21_H_20_O_11_ | 3.092 | 447.0936 | 285.0404[M-H-Glc]^-^, 255.0300[M-H-Glc-CHO]^-^, 151.0037[M-H-Glc-C_8_H_5_O_2_]^-^ |
| 195 | 12.98 | 6-O-E-feruloyl ajugol | 523.1821 | C_25_H_32_O_12_ | 2.384 | 523.1823 | 361.1303[M-H-Glc]^-^, 193.0505[M-H-Glc-C_9_H_12_O_3_]^-^ |
| 197^*^ | 13.08 | Cosmosiin | 431.0984 | C_21_H_20_O_10_ | 3.426 | 431.0988 | 269.0457[M-H-Glc]^-^, 151.0040[M-H-C_8_H_6_O]^-^, 117.0345[M-H-C_7_H_4_O_4_]^-^, 107.0135[M-H-C_9_H_6_O_3_]^-^ |
| 198 | 13.22 | Polygonumoside A | 555.1144 | C_27_H_24_O_13_ | 2.581 | 555.1148 | 393.0620[M-H-Glc]^-^, 349.0714[M-H-Glc-CO_2_]^-^ |
| 199 | 13.29 | Isorhamnetin-7-glucoside | 477.1038 | C_22_H_22_O_12_ | 3.139 | 477.1043 | 315.0513[M-H-Glc]^-^, 300.0278[M-H-Glc-CH_3_]^-^, 285.0407[M-H-Glc-CH_2_O]^-^ |
| 200 | 13.33 | Piceatannol-3-O-β-D-(6”-O-galloyl)-glucoside | 557.1301 | C_27_H_26_O_13_ | 2.410 | 557.1303 | 405.1176[M-H-C_7_H_4_O_4_]^-^, 243.0662[M-H-C_7_H_4_O_4_-Glc]^-^, 225.0547[M-H-C_7_H_4_O_4_-Glc-H_2_O]^-^ |
| 201 | 13.33 | Cynaroside | 447.0933 | C_21_H_20_O_11_ | 3.226 | 447.0936 | 285.0404[M-H-Glc]^-^, 257.0456[M-H-Glc-CO]^-^, 241.0506[M-H-Glc-CO_2_]^-^, 151.0037[M-H-Glc-C_8_H_5_O_2_],107.0138[M-H-Glc-C_9_H_6_O_4_]^-^ |
| 202 | 13.36 | Notoginsenoside R1 or ismoer | 977.5316 | C_47_H_80_O_18_ | 2.485 | 977.5340 | 931.5283[M-H]^-^, 799.4859[M-H-Xyl]^-^, 637.4348[M-H-Xyl-Glc]^-^, 475.3789[M-H-Xyl-2Glc]^-^ |
| 204^*^ | 13.39 | 1,5-dicaffeoylquinic acid | 515.1195 | C_25_H_24_O_12_ | 2.034 | 515.1195 | 353.0881[M-H-caffeoyl]^-^, 191.0561[M-H-2caffeoyl-]^-^, 179.0348[M-H-caffeoyl-C_7_H_10_O_5_]^-^, 173.0457[M-H-2caffeoyl-H_2_O]^-^, 135.0452[M-H-caffeoyl-C_7_H_10_O_5_-CO_2_]^-^ |
| 205 | 13.39 | Syringalide A-3’-rhamnopyranoside | 607.2032 | C_29_H_36_O_14_ | 2.533 | 607.2037 | 445.1702[M-H-caffeoyl]^-^, 299.1153[M-H-caffeoyl-Rha]^-^, 179.0352[M-H-Rha-Glc-C_8_H_8_O_2_]^-^ 161.0245[M-H-Rha-Glc-C_8_H_8_O_2_- H_2_O]^-^, 135.0452[M-H-Rha-Glc-C_8_H_8_O_2_-CO_2_]^-^ |
| 206 | 13.43 | Nepetin-7-O-glucoside | 477.1038 | C_22_H_22_O_12_ | 2.426 | 477.1039 | 315.0513[M-H-Glc]^-^, 300.0274[M-H-Glc-CH_3_]^-^,286.0488[M-H-Glc-CO-H] 271.0248[M-H-Glc-CO_2_]^-^,151.0037[C_7_H_3_O_4_]^-^ |
| 207 | 13.5 | Isorhamnetin-7-glucoside isomer | 477.1038 | C_22_H_22_O_12_ | 2.615 | 477.1040 | 315.0513[M-H-Glc]^-^, 300.0274[M-H-Glc-CH_3_]^-^, 285.0403[M-H-Glc-CH_2_O]^-^ |
| 209 | 13.53 | Baicalein | 269.0455 | C_15_H_10_O_5_ | 4.349 | 269.0456 | 241.0508[M-H-CO]^-^, 225.0559[M-H-CO_2_]^-^,197.0607[M-H-CO-CO_2_]^-^ |
| 210 | 13.57 | Jionoside C or isomer | 591.2083 | C_29_H_36_O_13_ | 2.575 | 591.2087 | 445.1721[M-H-caffeoyl]^-^, 299.1144[M-H-caffeoyl-Rha]^-^, 137.0610[M-H-caffeoyl-Rha-Glc]^-^ |
| 211 | 13.7 | Salsaside A or salsaside B | 577.1927 | C_28_H_34_O_13_ | 2.794 | 577.1932 | 415.1614[M-H-caffeoyl]^-^, 269.1039[M-H-caffeoyl-Rha]^-^, 161.0244[M-H-Rha-Glc-C_7_H_8_O]^-^ |
| 212 | 13.7 | Nigroside Ⅰ/Ⅱ | 621.2189 | C_30_H_38_O_14_ | 2.926 | 621.2196 | 487.1448[M-H-C_9_H_10_O]^-^, 459.1533[M-H-Glc]^-^ |
| 213 | 13.77 | Jionoside D or isomer | 637.2138 | C_30_H_38_O_15_ | 2.704 | 637.2144 | 491.1594[M-H-Rha]^-^, 461.1665[M-H-caffeoyl]^-^ |
| 214 | 13.81 | Cynaroside isomer | 447.0933 | C_21_H_20_O_11_ | 3.494 | 447.0938 | 285.0405[M-H-Glc]^-^, 241.0497[M-H-Glc-CO_2_]^-^, 151.0036[M-H-Glc-C_8_H_6_O_2_]^-^ |
| 215^*^ | 13.84 | Quercitrin | 447.0933 | C_21_H_20_O_11_ | 3.628 | 447.0938 | 301.0354[M-H-C_6_H_10_O_4_]^-^, 300.0277[M-H-C_6_H_11_O_4_]^-^,271.0250[M-H-C_7_H_12_O_5_]^-^, 255.0297[M-H-C_7_H_12_O_6_]^-^ |
| 216^*^ | 14.02 | Ginsenoside Rg1 | 845.4893 | C_42_H_72_O_14_ | 3.179 | 845.4920 | 845.4920[M+COOH]^-^, 799.4860[M-H-]^-^, 637.4330[M-H-Glc]^-^, 475.3802[M-H-2Glc]^-^, 391.2874[M-H-2Glc-C_6_H_12_]^-^ |
| 217 | 14.12 | Eriodictyol isomer | 287.0561 | C_15_H_12_O_6_ | 4.617 | 287.0563 | 151.0037[M-H-C_8_H_6_O_2_]^-^, 125.0244[M-H-C_9_H_6_O_3_]^-^, 107.0139[M-H-C_9_H_8_O_4_]^-^ |
| 218^*^ | 14.19 | Ginsenoside Re | 945.5428 | C_48_H_82_O_18_ | 0.463 | 945.5422 | 799.4857[M-H-Rha]^-^, 783.4907[M-H-Glc]^-^, 637.4339[M-H-Glc-Rha]^-^, 475.3798[M-H-2Glc-Rha]^-^ |
| 219 | 14.22 | Martynoside | 651.2294 | C_31_H_40_O_15_ | 5.410 | 651.2319 | 475.1831[M-H-C_10_H_8_O_3_]^-^, 175.0401[C_10_H_8_O_3_-H]^-^, 160.0166[C_10_H_8_O_3_-H-CH_3_]^-^ |
| 220 | 14.26 | Martynoside isomer | 651.2294 | C_31_H_40_O_15_ | 2.984 | 651.2303 | 475.1831[M-H-C_10_H_8_O_3_]^-^, 329.1251[M-H-C_10_H_8_O_3_-Rha]^-^, 167.07132[M-H-C_10_H_8_O_3_-Rha-Glc]^-^ |
| 221 | 14.29 | 2,3,5,4’-Tetrahydroxystilbene-2-O-(2”-O-acetyl)-β-D-glucoside | 447.1297 | C_22_H_24_O_10_ | 3.124 | 447.1300 | 243.0663[M-H-Glc-C_2_H_2_O]^-^, 225.0556[M-H-Glc-C_2_H_2_O-H_2_O]^-^, |
| 222 | 14.33 | Isochlorogenic acid A or isomer | 515.1195 | C_25_H_24_O_12_ | 2.499 | 515.1197 | 353.0879[M-H-caffeoyl]^-^, 191.0560[M-H-2caffeoyl-]^-^, 179.0349[M-H-caffeoyl-C_7_H_10_O_5_]^-^, 173.0454[M-H-2caffeoyl-H_2_O]^-^, 135.0451[M-H-caffeoyl-C_7_H_10_O_5_-CO_2_]^-^ |
| 223 | 14.36 | Emodin-8-O-β-D-glucoside or isomer | 431.0984 | C_21_H_20_O_10_ | 2.521 | 431.0984 | 269.0457[M-H-Glc]^-^, 241.0504[M-H-Glc-CO]^-^, 225.0557[M-H-Glc-CO_2_]^-^ |
| 224 | 14.46 | Salsaside F or isomer | 649.2138 | C_31_H_38_O_15_ | 3.117 | 649.2147 | 607.2050[M-H-C_2_H_2_O]^-^ |
| 225 | 14.53 | Isochlorogenic Acid C or isomer | 515.1195 | C_25_H_24_O_12_ | 3.101 | 515.1200 | 353.0879[M-H-caffeoyl]^-^, 191.0560[M-H-2caffeoyl-]^-^, 179.0351[M-H-caffeoyl-C_7_H_10_O_5_]^-^, 173.0455[M-H-2caffeoyl-H_2_O]^-^, 135.0451[M-H-caffeoyl-C_7_H_10_O_5_-CO_2_]^-^ |
| 226 | 14.53 | Tubuloside B | 665.2087 | C_31_H_38_O_16_ | 3.621 | 665.2100 | 623.1985[M-H-C_2_H_2_O]^-^, 461.1670[M-H-caffeoyl-C_2_H_2_O]^-^, 315.1093[M-H-caffeoyl-Rha-C_2_H_2_O]^-^, 179.0350[M-H-C_2_H_2_O-Rha-Glc-C_8_H_8_O_2_]^-^ |
| 227 | 14.85 | Taxifolin | 303.0510 | C_15_H_12_O_7_ | 4.029 | 303.0512 | 151.0401[M-H-C_7_H_4_O_4_], 123.0452[M-H-C_7_H_4_O_4_-CO] |
| 228 | 14.88 | Eutigoside A or isomer | 445.1504 | C_23_H_26_O_9_ | 2.901 | 445.1506 | 163.0400[M-H-C_8_H_8_O-Glc]^-^ |
| 231 | 15.09 | Emodin-3-ethyl ether or isomer | 297.0768 | C_17_H_14_O_5_ | 3.568 | 297.0768 | 282.0546[M-H-CH_3_]^-^, 269.0821[M-H-CO]^-^ |
| 233 | 15.26 | Cistanoside D | 651.2294 | C_31_H_40_O_15_ | 2.876 | 651.2302 | 505.1728[M-H-Rha]^-^, 487.1597[M-H-Rha-H_2_O]^-^, 475.1825[M-H-C_10_H_8_O_3_]^-^ |
| 236 | 15.36 | Osmanthuside B or isomer | 591.2083 | C_29_H_36_O_13_ | 2.778 | 591.2089 | 445.1568[M-H-Rha]^-^, 427.1430[M-H-Rha-H_2_O]^-^ |
| 237 | 15.54 | Martynoside isomer | 651.2294 | C_31_H_40_O_15_ | 2.876 | 651.2302 | 505.1727[M-H-Rha]^-^, 475.1807[M-H-C_10_H_8_O_3_]^-^, 193.0506[M-H-C_9_H_10_O_2_-Glc-Rha]^-^ |
| 238 | 15.68 | Malonyl-ginsenoside Rg1 | 885.4853 | C_45_H_74_O_17_ | 3.267 | 885.4871 | 841.4963[M-H-CO_2_]^-^, 799.4851[M-H-Mal]^-^, 781.4753[M-H-Mal-H_2_O]^-^, 637.4328[M-H-Mal-Glc]^-^, 475.3794[M-H-Mal-2Glc]^-^ |
| 239 | 15.68 | Malonyl ginsenoside rd | 1031.5432 | C_51_H_84_O_21_ | 2.699 | 1031.5449 | 987.5557[M-H-CO_2_]^-^, 945.5433[M-H-Mal]^-^, 783.4875[M-H-Mal-Glc]^-^, 637.4321[M-H-Mal-Glc-Rha]^-^, 475.3804[M-H-Mal-2Glc-Rha]^-^ |
| 241 | 15.88 | Yesanchinoside D (6’-O-acetyl- ginsenoside Rg1) | 887.4999 | C_44_H_74_O_15_ | 2.482 | 887.5021 | 841.4978[M-H]^-^, 781.4756[M-H-CH_3_COOH]^-^ |
| 242 | 16.02 | Cuscutoside A | 663.1931 | C_31_H_36_O_16_ | 2.848 | 663.1939 | 369.0982[M-H-Glc-C_5_H_8_O_4_]^-^, 219.0663[M-H-Glc-C_5_H_8_O_4_-C_8_H_6_O_3_]^-^ |
| 243^*^ | 16.12 | Eriodictyol | 287.0561 | C_15_H_12_O_6_ | 4.722 | 287.0564 | 151.0037[M-H-C_8_H_6_O_2_]^-^, 135.0452[M-H-C_7_H_4_O_4_]^-^, 125.0242[M-H-C_9_H_6_O_3_]^-^, 107.0138[M-H-C_9_H_8_O_4_]^-^ |
| 245 | 16.26 | Corchorifatty acid f | 327.2177 | C_18_H_32_O_5_ | 4.155 | 327.2180 | 309.2069[M-H-H_2_O]^-^, 291.1969[M-H-2H_2_O]^-^, 283.1918[M-H-CO_2_]^-^ |
| 249 | 16.7 | Rutaevine isomer | 485.1817 | C_26_H_30_O_9_ | 3.383 | 485.1823 | 345.1709[M-H-C_6_H_4_O_4_]^-^, 317.1758[M-H-C_7_H_4_O_5_]^-^, 205.0869[M-H-C_14_H_16_O_6_]^-^, 161.0970[M-H-C_15_H_16_O_8_]^-^,129.0556[M-H-C_20_H_20_O_6_]^-^ |
| 250 | 16.73 | Kaempferol-3-O-p-hydroxybenzoylglucoside | 567.1144 | C_28_H_24_O_13_ | 2.844 | 567.1149 | 447.0930[M-H-C_7_H_5_O_2_]^-^, 285.0406[M-H-C_13_H_14_O_7_]^-^, 255.0300[M-H-C_13_H_14_O_7_-CH_2_O]^-^, 151.0036[M-H-Glc-C_8_H_6_O_2_]^-^ |
| 252 | 16.94 | Rutaevine | 485.1817 | C_26_H_30_O_9_ | 2.950 | 485.1820 | 345.1709[M-H-C_6_H_4_O_4_]^-^, 317.1759[M-H-C_7_H_4_O_5_]^-^, 205.0870[M-H-C_14_H_16_O_6_]^-^, 161.0972[M-H-C_15_H_16_O_8_]^-^,129.0557[M-H-C_20_H_20_O_6_]^-^ |
| 253 | 17.36 | Pseudoginsenoside F11 | 845.4893 | C_42_H_72_O_14_ | 2.753 | 845.4916 | 799.4859[M-H]^-^, 653.4277[M-H-Rha]^-^, |
| 255 | 17.39 | 25R-Achyranthes Dioscin | 913.4791 | C_45_H_72_O_16_ | -0.352 | 913.4788 | 867.4593[M-H]^-^ |
| 256 | 17.49 | Physcion or isomer | 283.0612 | C_16_H_12_O_5_ | 4.169 | 283.0613 | 268.0376[M-H-CH_3_]^-^, 240.0249[M-H-CH_3_-CO]^-^, 212.0477[M-H-CH_3_-2CO] |
| 257 | 17.53 | Physcion-8-O-β-D-glucoside | 445.1140 | C_22_H_22_O_10_ | 3.003 | 445.1143 | 283.0613[M-H-Glc]^-^, 253.0511[M-H-Glc-CO-2H]^-^ |
| 258 | 17.53 | Quercetin-3-O-coumaroylgalactoside | 609.1250 | C_30_H_26_O_14_ | 2.739 | 609.1256 | 463.0887[M-H-C_9_H_7_O_2_]^-^, 301.0354[M-H-C_9_H_7_O_2_-Glc]^-^, 273.0400[M-H-C_9_H_7_O_2_-Glc-CO]^-^ |
| 259 | 17.56 | Tilianin isomer | 445.1140 | C_22_H_22_O_10_ | 2.868 | 445.1142 | 283.0613[M-H-Glc]^-^, 268.0386[M-H-Glc-CH_3_]^-^ |
| 260 | 17.84 | Rhein or isomer | 283.0248 | C_15_H_8_O_6_ | 4.295 | 283.0249 | 239.0720[M-H-CO_2_]^-^, 211.0755[M-H-CO_2_-CO]^-^ |
| 261 | 17.94 | Ginsenoside rf | 799.4849 | C_42_H_72_O_14_ | 0.984 | 799.4846 | 799.4859[M-H]^-^, 637.4356[M-H-Glc]^-^, 475.3789 [M-H-2Glc]^-^ |
| 262 | 17.98 | Corylifol B | 339.1238 | C_20_H_20_O_5_ | 3.007 | 339.1237 | 321.1133[M-H-H_2_O]^-^, 281.0818[M-H-H_2_O-C_3_H_6_]^-^ |
| 267 | 18.29 | Kaempferol-3-O-coumaroylglucoside | 593.1301 | C_30_H_26_O_13_ | 3.191 | 593.1309 | 447.0940[M-H-C_9_H_7_O_2_]^-^, 285.0865[M-H-C_9_H_7_O_2_-Glc]^-^, 255.0302[M-H-C_9_H_7_O_2_-Glc-CH_2_O]^-^, 151.0037[M-H-C_9_H_7_O_2_-Glc-C_8_H_6_O_2_]^-^ |
| 268^*^ | 18.36 | Luteolin | 285.0405 | C_15_H_10_O_6_ | 4.299 | 285.0406 | 241.0508[M-H-CO_2_]^-^, 133.0298[M-H-C_7_H_4_O_4_]^-^, 107.0138[M-H-C_9_H_6_O_4_]^-^ |
| 269 | 18.36 | Ginsenoside Ra3 or isomer | 1239.6379 | C_59_H_100_O_27_ | 1.489 | 1239.6387 | 1107.5999[M-H-C_5_H_8_O_4_]^-^ |
| 270^*^ | 18.39 | Ginsenoside Rb1 | 1107.5957 | C_54_H_92_O_23_ | 1.467 | 1107.5962 | 945.5427[M-H-Glc]^-^, 783.4886[M-H-2Glc]^-^, 621.4368[M-H-3Glc]^-^,459.3844[M-H-4Glc]^-^ |
| 271^*^ | 18.42 | Quercetin | 301.0354 | C_15_H_10_O_7_ | 3.524 | 301.0353 | 273.0417[M-H-CO]^-^, 257.0448[M-H-CO_2_]^-^, 229.0503[M-H-C_2_O_3_]^-^, 151.0037[M-H-C_8_H_6_O_3_]^-^ |
| 272 | 18.45 | Notoginsenoside R2 or ismoer | 769.4744 | C_41_H_70_O_13_ | 2.860 | 769.4745 | 815.4811[M+COOH]^-^, 769.4753[M-H]^-^, 637.4343[M-H-Xyl]^-^, 475.3792[M-H-Xyl-Glc]^-^ |
| 273 | 18.49 | Floralginsenoside B | 815.4798 | C_42_H_72_O_15_ | 3.154 | 815.4813 | 815.4813[M-H]^-^, 769.4753[M-H-CH_2_O_2_]^-^, 637.4344[M-H-Glc]^-^, 475.3792[M-H-2Glc]^-^ |
| 274 | 18.62 | 2-Acetylemodin-8-O-β-D-glucoside or isomer | 473.1089 | C_23_H_22_O_11_ | 2.795 | 473.1092 | 269.0457[M-H-Glc-C_2_H_2_O]^-^, 241.0510[M-H-Glc-C_2_H_2_O-CO]^-^, 225.0558 [M-H-Glc-C_2_H_2_O-CO_2_]^-^ |
| 275 | 18.62 | Ginsenoside Ra1 or isomer | 1209.6274 | C_58_H_98_O_26_ | 1.687 | 1209.6283 | 1077.5856[M-H-Xyl]^-^, 1047.5764[M-H-Glc]^-^, 945.5418[M-H-2Xyl]^-^, 915.5382[M-H-Xyl-Glc]^-^ |
| 276 | 18.69 | Ginsenoside Rb2 or isomer | 1123.5895 | C_53_H_90_O_22_ | 1.949 | 1123.5917 | 1077.5859[M-H]^-^, 915.5314[M-H-Gla]^-^, 783.4954[M-H-Gla-C_5_H_8_O_4_]^-^, 621.4409[M-H-2Gla-C_5_H_8_O_4_]^-^ |
| 277 | 18.69 | Quinquenoside R1 | 1149.6062 | C_56_H_94_O_24_ | 1.035 | 1149.6063 | 1107.5963[M-H-C_2_H_2_O]^-^, 1089.5863[M-H-C_2_H_4_O_2_]^-^, 945.5444[M-H-C_2_H_2_O-Glc]^-^, 783.4895[M-H-C_2_H_2_O-2Glc]^-^ |
| 280 | 18.82 | Ginsenoside rg2 | 829.4944 | C_42_H_72_O_13_ | 2.341 | 829.4963 | 783.4912[M-H]^-^, 637.4324[M-H-Rha]^-^, 475.3794[M-H-Rha-Glc]^-^, 391.2859[M-H-Rha-Glc-C_6_H_12_]^-^ |
| 281 | 18.85 | Kaempferol-3-O-α-rhamnoside | 431.0984 | C_21_H_20_O_10_ | 2.869 | 431.0985 | 269.0458[M-H-Glc]^-^, 241.0505[M-H-Glc-CO]^-^, |
| 282^*^ | 18.86 | Naringenin | 271.0612 | C_15_H_12_O_5_ | 4.243 | 271.0613 | 177.0193[M-H-C_6_H_6_O]^-^, 151.0037[M-H-C_8_H_8_O]^-^, 119.0502[M-H-C_7_H_4_O_4_]^-^, 107.0139[M-H-C_9_H_8_O_3_]^-^ |
| 283 | 18.86 | Malonyl-ginsenoside Rb1 | 1193.5961 | C_57_H_94_O_26_ | 1.341 | 1193.5966 | 1149.6057[M-H-CO_2_]^-^, 1107.5951[M-H-Mal]^-^, 945.5430[M-H-Mal-Glc]^-^, 783.47626[M-H-Mal-2Glc]^-^ |
| 284 | 18.89 | Ginsenoside rc | 1123.5895 | C_53_H_90_O_22_ | 1.843 | 1123.5916 | 1077.5858[M-H]^-^, 945.5447[M-H-Xyl]^-^, 783.4911[M-H-Xyl-Glc]^-^, 621.4399 [M-H-Xyl-2Glc]^-^, 459.3808 [M-H-Xyl-3Glc]^-^ |
| 285 | 18.95 | Malonyl-ginsenoside Rb2 or isomer | 1163.5855 | C_56_H_92_O_25_ | 1.638 | 1163.5863 | 1119.5972[M-H-CO_2_]^-^, 1077.5844[M-H-Mal]^-^, 945.5380[M-H-Mal-Xyl]^-^, 783.4866[M-H-Mal-Xyl-Glc]^-^, 621.4399[M-H-Mal-Xyl-2Glc]^-^,459.3854[M-H-Mal-Xyl-3Glc]^-^ |
| 286 | 18.99 | Ginsenoside rs1 | 1119.5957 | C_55_H_92_O_23_ | 1.451 | 1119.5962 | 1077.5862[M-H-C_2_H_2_O]^-^, 1059.5757[M-H-CC_2_H_4_O_2_]^-^, 945.5460[M-H-C_2_H_2_O-Xyl]^-^ |
| 287 | 19.19 | Ginsenoside ro | 955.4908 | C_48_H_76_O_19_ | 1.835 | 955.4915 | 793.4383[M-H-Glc]^-^, 569.3852[M-H-2Glc-CO_2_-H_2_O]^-^, 455.3529[M-H-2Glc-Glu]^-^ |
| 289 | 19.42 | Malonyl-ginsenoside Rb3 or isomer | 1163.5855 | C_56_H_92_O_25_ | 2.059 | 1163.5868 | 1119.5952[M-H-CO_2_]^-^, 1077.5858[M-H-Mal]^-^, 945.5467[M-H-Mal-Xyl]^-^, 783.4857[M-H-Mal-Xyl-Glc]^-^, 459.3859[M-H-Mal-Xyl-3Glc]^-^ |
| 290 | 19.56 | Ginsenoside rd | 945.5428 | C_48_H_82_O_18_ | 1.754 | 945.5434 | 783.4861[M-H-Glc]^-^,161.0459[Glc-H]^-^ |
| 291 | 19.63 | Pseudoginsenoside rt1 | 925.4802 | C_47_H_74_O_18_ | 2.559 | 925.4815 | 763.4322[M-H-Glc]^-^, 613.3760[M-H-Glc-Xyl-H_2_O]^-^, 569.3879[M-H-Glc-Xyl-H_2_O-CO_2_]^-^ |
| 292 | 19.63 | Malonyl-ginsenoside Rc | 1163.5855 | C_56_H_92_O_25_ | 1.956 | 1163.5867 | 1119.5972[M-H-CO_2_]^-^, 1077.5844[M-H-Mal]^-^, 1059.5753[M-H-Mal-H_2_O]^-^ |
| 294 | 19.8 | Malonyl-floralginsenoside Rd5 | 1031.5432 | C_51_H_84_O_21_ | 1.991 | 1031.5442 | 1031.5441[M-H]^-^ |
| 296 | 19.83 | Chikusetsusaponin IV | 925.4802 | C_47_H_74_O_18_ | 2.624 | 925.4816 | 763.4269[M-H-Glc]^-^, 701.4291[M-H-Glc-H_2_O-CO_2_]^-^, 595.3568[M-H-Glc-H_2_O-Ara-H_2_O]^-^, 551.3733[M-H-Glc-H_2_O-Ara-H_2_O-CO_2_]^-^,455.3524[M-H-Glc-Ara-Glua]^-^ |
| 297^*^ | 19.87 | Genistein | 269.0455 | C_15_H_10_O_5_ | 4.238 | 269.0456 | 225.0556[M-H-CO_2_]^-^, 201.0558[M-H-2CO-C],181.0660[M-H-2CO-2O], 151.0034[C_7_H_3_O_4_]^-^ |
| 299 | 20.07 | Chikusetsusaponin iva | 793.4380 | C_42_H_66_O_14_ | 2.857 |  | 631.3862[M-H-Glc]^-^,613.3765[M-H-Glc-H_2_O]^-^,587.4016[M-H-Glc-CO_2_]^-^,569.3834[M-H-Glc-H_2_O-CO_2_]^-^, 497.3641[M-H-Glc-CO_2_-C_3_H_6_O_3_]^-^, 455.3523[M-H-Glc-Glua]^-^ |
| 301 | 20.27 | Malonyl-ginsenoside XDⅡ | 1031.5432 | C_51_H_84_O_21_ | 2.108 | 1031.5443 | 987.5548[M-H-CO_2_]^-^ |
| 302^*^ | 20.31 | Apigenin | 269.0455 | C_15_H_10_O_5_ | 4.832 | 269.0458 | 225.0557[M-H-CO_2_]^-^, 151.0038[M-H-C_8_H_6_O]^-^, 117.0346[M-H-C_7_H_4_O_4_]^-^, 107.0139[M-H-C_9_H_6_O_3_]^-^ |
| 303 | 20.38 | Achyranthoside D | 1117.5072 | C_53_H_82_O_25_ | 0.838 | 1117.5071 | 997.5019[M-H-COOH-CH_2_OH-CO_2_]^-^, 955.4911[M-H-C_5_H_6_O_6_]^-^, 793.4394[M-H-C_5_H_6_O_6_-Glc]^-^, 731.4387[M-H-C_5_H_6_O_6_-Glc-CO_2_-H_2_O], 455.3542[M-H-C_5_H_6_O_6_-2Glc-Gla]^-^ |
| 304 | 20.41 | Nepetin or isomer | 315.0510 | C_16_H_12_O_7_ | 4.256 | 315.0513 | 300.0278[M-H-CH_3_]^-^, 271.0246[M-H-CH_3_-CHO]^-^, 255.0299[M-H-CH_3_-CHO_2_]^-^ |
| 306 | 20.68 | Citreorosein | 285.0405 | C_15_H_10_O_6_ | 3.879 | 285.0405 | 257.0456[M-H-CO]^-^, 241.0504[M-H-CO_2_]^-^, 227.0346[M-H-CO-CH_2_O]^-^ |
| 308 | 20.81 | Imperatorin or isomer | 269.0819 | C_16_H_14_O_4_ | 4.105 | 269.0819 | 254.0585[M-H-CH_3_]^-^, 239.0350[M-H-2CH_3_]^-^, 211.0402[M-H-2CH_3_-CO]^-^ |
| 310 | 21.15 | 3-O-β-D-glua-β-D-glua-28-O-β-D-Glc oleanolic acid | 969.4701 | C_48_H_74_O_20_ | 2.733 | 969.4716 | 835.4495[M-H-C_4_H_6_O_5_]^-^, 793.4411[M-H-Glua]^-^, 673.3964[M-H-Glua-C_4_H_8_O_4_]^-^, 611.3953[M-H-Glua-C_4_H_8_O_4_-CO_2_-H_2_O]^-^, 569.3856[M-H-Glua-Glc-CO_2_-H_2_O]^-^, 455.3534[M-H-2Glua-Glc]^-^ |
| 311 | 21.18 | Ginsenoside F2 or isomer | 829.4944 | C_42_H_72_O_13_ | 2.775 | 829.4967 | 783.4912[M-H-Glc]^-^, 621.4379[M-H-Glc]^-^ |
| 312 | 21.29 | Achyranthoside C | 955.4544 | C_47_H_72_O_20_ | 2.218 | 955.4554 | 835.4496[M-H-CO_2_-CH_2_OHCOOH]^-^, 793.4387[M-H-C_5_H_6_O_6_]^-^, 673.3943[M-H-CO_2_-CH_2_OHCOOH-Glc]^-^, 569.3839[M-H-C_5_H_6_O_6_-Glc-H_2_O-CO_2_]^-^, 455.3539[M-H-C_5_H_6_O_6_-Glc-Glua]^-^ |
| 320 | 21.93 | 9-hpode | 311.2228 | C_18_H_32_O_4_ | 3.997 | 311.2229 | 293.2117[M-H-H_2_O]^-^, 275.2015[M-H-2H_2_O]^-^, 171.1023[M-H-C_9_H_16_O]^-^ |
| 321 | 21.99 | Achyranthoside B or isomer | 953.4388 | C_47_H_70_O_20_ | 2.003 | 953.4396 | 909.4507[M-H-CO_2_]^-^, 851.4442[M-H-C_2_H_2_O_2_]^-^, 793.4391[M-H-C_4_H_4_O_4_]^-^ |
| 327 | 22.56 | Decanoic acid | 171.1391 | C_10_H_20_O_2_ | 6.566 | 171.1391 | 171.1391[M-H]^-^ |
| 336 | 22.96 | Zingibroside R1 | 793.4380 | C_42_H_66_O_14_ | 2.618 | 793.4390 | 631.3857[M-H-Glc]^-^, 569.3850[M-H-C_7_H_12_O_8_]^-^,455.3526[M-H-C_12_H_18_O_11_]^-^ |
| 347 | 24.59 | Pseudo-ginsenoside Rp1 | 763.4274 | C_41_H_64_O_13_ | 2.334 | 763.4281 | 455.3557[M-H-Xyl-Glu]^-^ |
| 351^*^ | 24.86 | Aloe-emodin | 269.0455 | C_15_H_10_O_5_ | 4.349 | 269.0456 | 225.0559[M-H-CO-O]^-^, 240.0426[M-H-CHO]^-^ |
| 352^*^ | 25.06 | Emodin | 269.0455 | C_15_H_10_O_5_ | 4.238 | 269.0456 | 241.0507[M-H-CO]^-^,225.0559[M-H-CO_2_]^-^,210.0320[M-H-CO_2_-CH_3_]^-^,197.0612[M-H-CO-CO_2_]^-^, 181.0656[M-H-C_2_H_3_O_3_]^-^, 240.0431[M-H-CHO]^-^, 213.0557[M-H-CO-CO]^-^ 182.0363[M-H-CO_2_-CH_3_-CO]^-^ |
| 354 | 25.29 | 28-desglucosylchikusetsusaponin IVa | 631.3852 | C_36_H_56_O_9_ | 2.756 | 631.3858 | 555.3691[M-H-C_2_H_4_O_3_]^-^, 509.3672[M-H-C_2_H_4_O_3_-HCOOH]^-^, 455.3536[M-H-Glua]^-^ |
| 359 | 25.96 | Linoleic acid isomer | 279.2319 | C_18_H_30_O_2_ | 3.557 | 279.2329 | 279.2329[M-H]^-^ |
| 365 | 27.88 | Bakuchiol | 255.1754 | C_18_H_24_O | 4.460 | 255.1755 | 172.0895[M-H-C_6_H_11_]^-^ |

Note: t_R_: retention time; *: compound identified by compared with the reference standard

**Supplementary Table S3. Identification of compounds from different samples by UPLC-Q Exactive-Orbitrap HRMS**

| Proposed compounds | Gastric sample | Intestinal sample | Hepatic sample | Systemic sample | Urine sample | Fece sample |
| --- | --- | --- | --- | --- | --- | --- |
| Arginine | + | - | + | - | + | - |
| Raffinose | - | - | - | - | - | - |
| Betaine | + | + | + | + | + | + |
| Palatinose | - | - | - | - | - | - |
| Stachyose | - | - | - | - | - | - |
| Mannitol | + | - | - | - | - | - |
| Sucrose | - | - | - | - | - | - |
| Gluconic acid | + | - | - | - | + | - |
| Adenine | + | - | - | - | + | + |
| Quinic acid | + | - | - | + | - | - |
| Guanosine | + | - | - | - | - | - |
| Achyranthine | - | - | - | - | - | - |
| Phenylacetaldehyde | + | + | - | - | + | + |
| Allantoin | - | - | - | - | - | - |
| D-glucaric acid | + | - | - | - | + | - |
| Proline | + | + | + | + | + | - |
| Nicotinic acid | + | + | - | - | + | + |
| Candicine | + | + | - | - | + | + |
| Adenosine | - | - | + | + | - | - |
| L-Pyroglutamic acid | + | + | + | + | - | - |
| Leucine | + | + | + | - | + | - |
| Tyrosine | + | + | + | - | - | - |
| Citric acid | + | - | - | - | - | - |
| Guanine | + | - | - | - | - | + |
| Catalpol | + | - | - | - | - | - |
| Rehmannioside D | + | - | - | - | - | - |
| Phenylalanine | + | + | + | + | - | + |
| Gallic acid | - | - | - | - | - | - |
| Ajugol | + | + | - | - | - | - |
| 3-Hydroxymethyl-2-furfural | + | - | - | - | + | - |
| Maltol or isomer | + | + | + | + | + | + |
| 5-hydroxymethylfurfural | + | + | + | + | + | + |
| Tryptophan | + | + | + | + | + | - |
| Geniposidic acid | + | + | - | - | + | - |
| Higenamine | - | - | - | - | - | - |
| N-caffeoyltryptophan | + | + | + | + | + | - |
| Protocatechuic acid-O-glucoside | - | - | - | - | - | - |
| Aucubin | + | - | - | - | - | - |
| Vanillic acid or isomer | - | - | - | - | - | - |
| Decaffeoyl-verbascoside isomer | - | - | - | - | - | - |
| Vanillin | + | - | - | + | + | + |
| Mussarnosidic acid | + | - | - | - | - | - |
| Decaffeoyl-acteoside | + | - | - | - | - | - |
| Lotusine | + | + | - | - | + | + |
| 8-epiloganic acid | - | - | - | - | - | - |
| Dihydroxy-benzoic acid or isomer | + | - | - | - | + | - |
| Gentisic acid | + | - | - | - | + | - |
| Magnocurarine | + | + | - | - | - | + |
| Veratric acid | + | - | + | - | + | + |
| 3,4-Dihydro-1-[(4-hydroxyphenyl)methyl]-7-methoxy-2-methyl-6-isoquinolinol. | - | - | - | - | - | - |
| Cryptochlorogenic acid isomer | - | - | - | - | - | - |
| 7-hydroxycoumarin | + | - | - | - | - | - |
| Neochlorogenic acid | + | + | - | - | - | - |
| Protocatechuic aldehyde | + | + | + | + | + | + |
| 3-O-(4'-O-Caffeoylglucosyl)quinic acid | - | - | - | - | - | - |
| Protocatechualdehyde or isomer | + | + | - | + | + | + |
| Lamiol | - | - | - | - | - | - |
| Phellodendrine | + | + | + | + | - | + |
| Phellodendrine derivative | + | - | - | - | - | - |
| Abscisic acid | + | - | - | - | - | - |
| Kanokside C | + | - | - | - | - | - |
| Sanjoinine K | - | - | - | - | + | + |
| 3‑O‑feruloylquinic acid glucoside | - | - | - | - | - | - |
| Magnoflorine | + | + | + | + | + | + |
| N-methylhigenamine7-glucopyranoside | - | - | - | - | - | - |
| Rehmannioside A | + | - | - | - | - | - |
| 5-O-(3'-O-Caffeoylglucosyl)quinic acid | - | - | - | - | - | - |
| Phthalic acid | + | - | - | - | - | + |
| P-Hydroxybenzoic Acid or isomer | + | - | - | - | + | + |
| Laudanosine | + | - | - | - | - | - |
| Plantagoguanidinic acid A | + | + | + | - | - | + |
| Darendoside B | + | - | - | - | - | - |
| Plantagoguanidinic acid A or isomer | + | + | + | + | - | - |
| Cassythidine | + | + | - | - | + | - |
| Oblongine | + | + | - | - | - | + |
| Esculetin | + | - | - | - | - | + |
| Cianidanol | - | - | - | - | - | - |
| Dauricine | + | + | - | - | - | + |
| Procyanidin B1 | - | - | - | - | - | - |
| N‑methyltetrahydrocolumbamine | + | - | - | - | - | + |
| 3-O-feruloylquinic acid | + | + | + | - | + | - |
| Cryptochlorogenic acid | + | + | - | - | - | - |
| Quercetin-3-o-galactoside-7-o-glucoside | + | - | - | - | - | - |
| Rhamnopyranosyl vanilloyl | + | - | - | - | - | - |
| Chlorogenic acid | + | + | - | - | - | - |
| Dasycarpamin | - | - | - | - | - | + |
| Phellodendrine isomer | + | - | - | - | - | + |
| 3-O-Caffeoylqunic acid | - | - | - | - | - | - |
| Quercetin-3-O-caffeoylgalactoside | + | - | - | - | - | - |
| Tembetarine | + | + | - | - | - | + |
| Sec-hydroxyaeginetic acid | - | - | - | - | - | + |
| Syringic acid-4-O-α-L-rhamnoside | - | - | - | - | - | - |
| Cistanoside F or isomer | - | - | - | - | - | - |
| Caffeic acid | - | - | - | - | - | + |
| Senkyunolide J or isomer | - | - | - | - | + | + |
| Rehmapicroside or isomer | + | + | - | - | - | - |
| 4-O-Caffeoylqunic acid | - | - | - | - | - | - |
| Epicatechin | - | - | - | - | - | - |
| Cistantubuloside C1 or isomer | - | - | - | - | - | - |
| Rumejaposide D or isomer | - | - | - | - | - | - |
| Rehmapicrogenin | + | + | + | - | + | + |
| Tetrahydropalmatine | + | + | - | - | + | + |
| Kaempferol-dihexoside | - | - | - | - | - | - |
| Yuanhunine or isomer | + | + | - | - | + | + |
| Psoralenoside | + | + | + | - | - | - |
| Hydroxyberberine | + | + | - | - | + | + |
| Alaschanioside A | - | - | - | - | - | - |
| Berberastine isomer | + | + | - | - | + | + |
| Demethyleneberberine | - | - | - | - | + | - |
| Isopsoralenoside | + | - | - | - | - | - |
| Purpureaside C | - | - | - | - | - | - |
| Noroxyhydrastinine | - | - | - | - | - | - |
| Acetyl-epicatechin-O-glucoside | - | - | - | - | - | - |
| Demethyleneberberine isomer | + | + | - | - | + | - |
| Polypodine B | + | - | + | - | - | - |
| Echinacoside | - | - | - | - | - | - |
| Apioside or isomer | + | + | - | - | + | - |
| 4-p-coumaroylquinicacid | + | + | - | - | - | - |
| 4-O-feruloylquinic acid | + | + | + | - | + | - |
| Cinnamic acid | - | - | - | - | - | - |
| 5-O-feruloylquinic acid isomer | + | + | + | - | + | - |
| 5-O-feruloylquinic acid | + | + | - | - | + | - |
| Rehmapicroside or isomer | - | - | - | - | - | - |
| Isoplatydesmine or isomer | + | - | - | + | + | - |
| Daidzin | - | - | - | - | - | - |
| Β-hydroxyacteoside | - | - | - | - | - | - |
| Jionoside A1/A2 | - | - | - | - | + | - |
| Columbamine | + | + | - | - | + | + |
| N-methyl canadine | + | - | - | - | - | + |
| Kankanoside A | + | + | + | + | - | - |
| Thalifendine or isomer | - | + | - | + | + | + |
| Jateorhizine | + | + | - | - | + | + |
| Cistanoside A or isomer | + | + | - | - | + | - |
| Poliumoside | - | - | - | - | - | - |
| Lariciresinol glucoside or isomer | - | - | - | - | - | - |
| Plantamajoside | - | - | - | - | - | - |
| Anisic acid | - | - | - | - | - | - |
| Campneoside II | - | - | - | - | - | - |
| 6-Methoxyl-2-Acetyl-3-methyljuglone-8-O-β-D-glucoside | - | - | - | - | - | - |
| Stachysterone C | + | + | - | - | - | - |
| β-ecdysterone | - | - | - | - | - | - |
| Ferulic acid | + | + | - | - | + | + |
| 4,7-Dihydroxy-3-butylphthalide or isomer | - | - | - | - | - | - |
| Tetrahydroxystilbene-O-di-glucoside | - | - | - | - | - | - |
| Plantagoside | - | - | - | - | - | - |
| Berberrubine | + | + | - | + | + | + |
| Conicaoside | + | - | - | - | - | - |
| Dehydrocorybulbine | - | + | - | - | - | + |
| 25R-Achyranthes bidentata | + | + | + | - | - | + |
| Epiberberine | - | - | - | + | - | + |
| Coptisine | + | - | - | - | + | + |
| Tubuloside A | - | - | - | - | - | - |
| 25S-Achyranthes bidentata | + | + | - | - | - | + |
| Palmatine | - | + | - | - | - | + |
| Berberine | - | - | - | - | - | - |
| Isoacteoside isomer | - | - | - | - | - | - |
| Jionoside B1/B2 | + | + | - | - | - | - |
| Acteoside | - | - | - | - | - | - |
| (Z)-4',6-dihydroxyaurone6-glucoside or isomer | - | - | - | - | - | - |
| Isoferulic acid | + | - | - | - | + | - |
| Senkyunolide F or isomer | + | + | - | - | + | - |
| Pinoresinol glucoside | - | - | - | - | - | - |
| Syringaresinol glucoside | + | - | - | - | - | - |
| Cistanoside K or isomer | - | - | - | - | - | - |
| Phellamurin | - | - | - | - | - | - |
| Tetrahydroxystilbene glucoside | - | + | - | - | + | + |
| 3,4,5,4’-tetrahydroxystilbene isomer | - | - | - | - | - | + |
| Quercetin-3-O-apiosyl-(1→2)-galactoside | - | - | - | - | - | - |
| Polygonumosides C | - | - | - | - | - | - |
| Calceolarioside B | - | - | - | - | - | - |
| Azelaic acid | + | + | + | + | + | + |
| Epicatechin-O-gallate | - | + | - | - | - | - |
| Plantainoside D | - | - | - | - | - | - |
| Eucommin A or isomer | - | - | - | - | - | - |
| Cuscutoside D | - | - | - | - | - | - |
| 2′-acetylpoliumoside | - | - | - | - | - | - |
| Acteoside isomer | - | - | - | - | - | - |
| Kankanoside G or isomer | - | - | - | - | - | - |
| 6-Hydroxy-luteolin-7-O-glucoside | - | - | - | - | - | - |
| Hyperoside | + | + | - | - | - | - |
| Isoquercetin | + | + | - | - | - | - |
| Spiraeoside isomer | - | + | - | - | - | - |
| Acteoside isomer | - | - | - | - | - | - |
| Tetrahydroxystilbene-O-(galloyl)-glucoside | - | - | - | - | - | - |
| Leucosceptoside A | - | - | - | - | - | - |
| Jiocarotenoside A1/A2 | - | - | - | - | - | - |
| Dunnisinoside | - | - | - | - | - | - |
| Kaempferol 3-apiosyl-(1->2)-glucoside | - | - | - | - | - | - |
| Cistanoside C | - | - | - | - | - | - |
| Rutin | - | - | - | - | - | - |
| Oxyberberine | + | - | - | - | - | + |
| Rhoifolin | + | + | - | - | - | - |
| 2-acetylacteoside | - | - | - | - | - | - |
| Astragalin isomer | - | - | - | - | - | - |
| 6-O-E-feruloyl ajugol | + | - | - | - | - | - |
| Kaempferol-3-O-β-D-glucoside | - | - | - | - | - | - |
| Cosmosiin | + | - | - | - | - | - |
| Polygonumoside A | + | - | - | - | - | - |
| Isorhamnetin-7-glucoside | - | - | - | - | - | - |
| Piceatannol-3-O-β-D-(6”-O-galloyl)-glucoside | - | - | - | - | - | - |
| Cynaroside | + | + | - | - | - | - |
| Notoginsenoside R1 or ismoer | + | + | - | - | - | - |
| Astragalin | + | + | - | - | - | - |
| 1,5-dicaffeoylquinic acid | - | + | - | - | - | - |
| Syringalide A-3’-rhamnopyranoside | - | + | - | - | - | - |
| Nepetin-7-O-glucoside | + | - | - | - | - | - |
| Isorhamnetin-7-glucoside isomer | + | - | - | - | - | - |
| Isorhamnetin isomer | - | - | - | - | - | - |
| Baicalein | - | - | - | - | + | + |
| Jionoside C or isomer | + | - | - | - | - | - |
| Salsaside A or salsaside B | - | - | - | - | - | - |
| Nigroside Ⅰ/Ⅱ | - | - | - | - | - | - |
| Jionoside D or isomer | - | - | - | - | - | - |
| Cynaroside isomer | - | - | - | - | - | - |
| Quercitrin | + | - | - | - | - | - |
| Ginsenoside Rg1 | + | + | + | - | - | - |
| Eriodictyol isomer | - | - | - | - | + | - |
| Ginsenoside Re | - | - | - | - | - | - |
| Martynoside | - | - | - | - | - | - |
| Martynoside isomer | + | + | - | - | + | - |
| 2,3,5,4’-Tetrahydroxystilbene-2-O-(2”-O-acetyl)-β-D-glucoside | - | - | - | - | - | - |
| Isochlorogenic acid A or isomer | - | - | - | - | - | - |
| Emodin-8-O-β-D-glucoside or isomer | - | - | - | - | - | - |
| Salsaside F or isomer | - | - | - | - | - | - |
| Isochlorogenic Acid C or isomer | - | - | - | - | - | - |
| Tubuloside B | - | - | - | - | - | - |
| Taxifolin | - | - | - | - | - | - |
| Eutigoside A or isomer | - | - | - | - | - | - |
| Tomentosanol D or isomer | + | + | - | - | + | + |
| N-feruloyl-3-methoxytyramine | + | + | - | - | + | - |
| Emodin-3-ethyl ether or isomer | - | - | - | - | - | - |
| Butylidene phthalide or isomer | - | + | - | - | + | + |
| Cistanoside D | + | + | - | - | + | - |
| Astrapterocarpan | + | + | - | - | + | + |
| Senkyunolide | - | - | - | - | + | + |
| Osmanthuside B or isomer | - | - | - | - | - | - |
| Martynoside isomer | - | - | - | - | - | - |
| Malonyl-ginsenoside Rg1 | - | - | - | - | - | - |
| Malonyl ginsenoside rd | - | - | - | - | - | - |
| Psoralen | + | + | + | + | + | + |
| Yesanchinoside D (6’-O-acetyl- ginsenoside Rg1) | - | - | - | - | - | - |
| Cuscutoside A | - | - | - | - | - | - |
| Eriodictyol | - | - | - | - | + | + |
| Hinokinin | - | - | - | - | + | + |
| Corchorifatty acid f | + | - | - | - | - | + |
| Angelicin | + | + | - | + | + | + |
| Daidzein | + | + | - | - | - | + |
| Calycosin | + | + | - | - | + | + |
| Rutaevin isomer | - | + | - | - | - | - |
| Kaempferol-3-O-p-hydroxybenzoylglucoside | - | - | - | - | - | - |
| Pepper alkaloid | + | + | - | + | - | - |
| Rutaevine | - | - | - | - | - | - |
| Pseudoginsenoside F11 | + | + | - | - | - | - |
| Oleanolic acid or isomer | - | - | - | - | - | - |
| 25R-Achyranthes Dioscin | - | - | - | - | - | - |
| Physcion or isomer | + | - | - | - | - | - |
| Physcion-8-O-β-D-glucoside | + | - | - | - | - | - |
| Quercetin-3-O-coumaroylgalactoside | - | - | - | - | - | - |
| Tilianin isomer | - | - | - | - | - | - |
| Rhein or isomer | - | - | - | - | - | - |
| Ginsenoside rf | - | - | - | - | - | - |
| Corylifol B | + | - | - | - | - | + |
| Palmitic acid | - | - | - | - | - | - |
| Dehydrocorybulbine or isomer | - | - | - | - | - | - |
| Bavachromanol | + | + | - | - | + | + |
| Butylphthalide | + | - | - | + | + | - |
| Kaempferol-3-O-coumaroylglucoside | - | - | - | - | - | - |
| Luteolin | - | - | - | - | - | + |
| Ginsenoside Ra3 or isomer | - | - | - | - | - | - |
| Ginsenoside Rb1 | - | - | - | - | - | - |
| Quercetin | - | - | + | - | - | + |
| Notoginsenoside R2 or ismoer | + | - | - | - | - | - |
| Floralginsenoside B | - | - | - | - | - | - |
| 2-Acetylemodin-8-O-β-D-glucoside or isomer | - | - | - | - | - | - |
| Ginsenoside Ra1 or isomer | - | - | - | - | - | - |
| Ginsenoside Rb2 or isomer | - | - | - | - | - | - |
| Quinquenoside R1 | - | - | - | - | - | - |
| Euchrenone a7 or isomer | + | + | - | - | + | + |
| 10,12-Octadecanedioic acid | - | - | + | - | + | + |
| Ginsenoside Rg2 | - | + | - | - | - | + |
| Kaempferol-3-O-α-rhamnoside | - | - | - | - | - | - |
| Naringenin | - | - | - | - | + | - |
| Malonyl-ginsenoside Rb1 | - | + | - | - | - | - |
| Ginsenoside rc | + | + | + | - | - | - |
| Malonyl-ginsenoside Rb2 or isomer | - | - | - | - | - | - |
| Ginsenoside rs1 | + | - | - | - | - | - |
| Ginsenoside ro | + | + | + | - | - | - |
| Corylidin or isomer | - | - | - | - | - | - |
| Malonyl-ginsenoside Rb3 or isomer | - | - | - | - | - | - |
| Ginsenoside rd | - | - | - | - | - | - |
| Pseudoginsenoside rt1 | + | - | - | - | - | - |
| Malonyl-ginsenoside Rc | - | - | - | - | - | - |
| Limonin | - | - | - | - | - | - |
| Malonyl-floralginsenoside Rd5 | - | - | - | - | - | - |
| 3-Hydroxybakuchiol or isomer | + | + | - | - | + | + |
| Chikusetsusaponin IV | + | + | - | - | - | - |
| Genistein | + | + | - | - | + | + |
| Cyasterone or isomer | - | - | - | - | - | - |
| Chikusetsusaponin iva | + | + | - | - | - | - |
| Psoralenol or isomer | + | + | - | - | + | + |
| Malonyl-ginsenoside XDⅡ | - | - | - | - | - | - |
| Apigenin | - | + | - | - | + | + |
| Achyranthoside D | + | - | - | - | - | - |
| Nepetin or isomer | - | - | - | - | - | - |
| Schisandrol A | + | + | + | + | - | - |
| Citreorosein | - | + | - | - | + | + |
| Kaempferol | - | - | - | - | + | + |
| Imperatorin or isomer | - | - | - | - | - | - |
| Senkyunolide A or isomer | + | + | - | + | - | + |
| 3-O-β-D-glua-β-D-glua-28-O-β-D-Glc oleanolic acid | - | - | - | - | - | - |
| Ginsenoside F2 or isomer | - | - | - | - | - | + |
| Achyranthoside C | + | - | - | - | - | - |
| Gomisin O or isomer | - | - | - | + | - | - |
| Gomisin D | - | + | - | + | - | - |
| Gomisin R | - | + | - | + | - | - |
| Wogonin | - | - | - | - | - | - |
| Schisandrol B | + | - | + | - | - | - |
| Bavachin or isomer | + | + | - | + | + | + |
| Obacunone | - | - | - | + | - | - |
| 9-hpode | + | + | + | + | + | + |
| Achyranthoside B or isomer | + | - | - | - | - | - |
| Panaxydol | - | + | - | - | - | + |
| Xambioona or isomer | + | - | - | - | - | - |
| Gomisin J | + | - | - | - | - | + |
| Tigloylgomisin H | + | - | - | - | - | - |
| Schisantherin B | + | + | - | - | - | - |
| Decanoic acid | - | - | - | - | - | - |
| Corylifolinin isomer | - | - | - | - | - | - |
| Hydroxylonchocarpin isomer | + | + | - | + | + | + |
| Coryfolin | + | + | - | - | + | + |
| Neobavaisoflavone isomer | - | - | - | - | - | - |
| Benzoylgomisin H | + | + | - | + | - | - |
| Ligustilide | + | + | - | + | + | + |
| Angeloyl gomisin H | + | + | - | + | - | - |
| Linolenic acid | + | + | + | + | + | + |
| Zingibroside R1 | + | - | - | - | - | - |
| Chrysophanol or isomer | + | - | - | - | - | + |
| Psoralidin | - | - | - | - | - | - |
| Schisandrin C | - | - | - | - | - | - |
| Erythrinin A isomer | + | + | - | + | + | + |
| Schisantherin A | + | - | - | - | - | - |
| Corylin | + | + | - | - | + | + |
| Alloimperatorin | - | - | - | - | - | - |
| Gomisin G | + | - | - | + | - | - |
| Gomisin K1 or isomer | + | - | - | - | - | + |
| Gomisin L1 or isomer | - | - | - | - | - | + |
| Pseudo-ginsenoside Rp1 | + | - | - | - | - | - |
| Bavachinin A | + | - | - | + | - | + |
| Licarin A | + | - | - | - | + | + |
| 8-geranyloxypsoralen | - | - | - | - | - | - |
| Aloe-emodin | + | + | + | + | + | + |
| Emodin | + | + | + | + | + | + |
| Senkyunolide P or isomer | - | - | - | - | - | - |
| 28-desglucosylchikusetsusaponin IVa | + | - | - | - | - | - |
| Schizandrin A | + | + | - | + | - | + |
| Gemmazone | - | - | - | - | - | - |
| Calarene | - | - | - | - | - | - |
| Corylifolinin | + | + | - | - | + | + |
| Linoleic acid isomer | - | - | - | - | - | - |
| Angelicide or isomer | + | - | - | - | - | + |
| Corylifol A | - | - | - | - | - | - |
| Levistilide A | + | - | - | - | - | + |
| Schisandrin B | + | + | - | + | - | + |
| Benzoylgomisin O or isomer | + | - | - | - | - | - |
| Bakuchiol | - | - | - | - | - | + |
| 13-docosenamide | - | - | + | - | + | - |

Note: +: components detected in the sample. -: components not detected in the sample.

**Supplementary Table S4. Top 40 compounds of deep learning model**

| **Compounds** | **Q8N6T7** | **Q13131** | **Q9Y478** |
| --- | --- | --- | --- |
| Achyranthoside D | 1 | 1 | 1 |
| Achyranthoside C | 1 | 1 | 1 |
| Pseudoginsenoside F11 | 1 | 1 | 1 |
| jionoside B1/B2 | 1 | 1 | 1 |
| Ginsenoside RG1 | 1 | 1 | 1 |
| Ginsenoside rb1 | 1 | 1 | 1 |
| Ginsenoside Re | 1 | 1 | 1 |
| Ginsenoside Rg2 | 1 | 1 | 1 |
| Ginsenoside Rc | 1 | 1 | 1 |
| Ginsenoside Rd | 1 | 1 | 1 |
| Ginsenoside F2 | 1 | 1 | 1 |
| Malonyl Ginsenoside Rd | 1 | 1 | 1 |
| Ginsenoside Rf | 1 | 1 | 1 |
| Floralginsenoside B | 1 | 1 | 1 |
| Ginsenoside Ra3 or isomer | 1 | 1 | 1 |
| Ginsenoside Ra1 or isomer | 1 | 1 | 1 |
| Quinquenoside R1 | 1 | 1 | 1 |
| Ginsenoside Rs1 | 1 | 1 | 1 |
| Notoginsenoside R1 | 1 | 1 | 1 |
| Notoginsenoside R2 | 1 | 1 | 1 |
| Ginsenoside Rb2 | 1 | 1 | 1 |
| Malonyl-ginsenoside Rb2 | 1 | 1 | 1 |
| Cistantubuloside C1 | 1 | 1 | 1 |
| Corchorifatty Acid F | 1 | 1 | 0.999963 |
| Darendoside B | 1 | 1 | 1 |
| Decaffeoylacteoside | 1 | 1 | 1 |
| Decaffeoyl-verbascoside | 1 | 1 | 1 |
| Jiocarotenoside A1/A2 | 1 | 1 | 1 |
| Jionoside A1/A2 | 1 | 1 | 1 |
| Nigroside Ⅰ/Ⅱ | 1 | 1 | 1 |
| Ginsenoside Ro | 0.999999 | 1 | 1 |
| Pseudoginsenoside Rt1 | 0.999999 | 1 | 1 |
| N-methylhigenamine7-glucopyranoside | 0.999999 | 1 | 1 |
| Chikusetsusaponin IV | 0.999998 | 1 | 1 |
| Malonyl-ginsenoside Rb1 | 0.999998 | 1 | 1 |
| Jionoside C | 0.999998 | 1 | 1 |
| Limonin | 0.999997 | 1 | 1 |
| Chikusetsusaponin IVa | 0.999997 | 1 | 1 |
| Achyranthoside B or isomer | 0.999997 | 1 | 1 |
| Zingibroside R1 | 0.999995 | 1 | 1 |
